# Cultivation of a Cu/HMPC catalyst from a hyperaccumulating mustard plant for highly efficient and selective coupling reactions under mild conditions†

**DOI:** 10.1039/c7ra12470h

**Published:** 2018-01-24

**Authors:** Mayakrishnan Gopiraman, Kai Wei, Ke-Qin Zhang, Ill-Min Chung, Ick Soo Kim

**Affiliations:** Nano Fusion Technology Research Group, Division of Frontier Fibers, Institute for Fiber Engineering (IFES), Interdisciplinary Cluster for Cutting Edge Research (ICCER), Shinshu University Tokida 3-15-1 Ueda Nagano Prefecture 386-8567 Japan kim@shinshu-u.ac.jp; Department of Applied Bioscience, College of Life & Environment Science, Konkuk University 120 Neungdong-ro, Gwangjin-gu Seoul 05029 South Korea imchung@konkuk.ac.kr; National Engineering Laboratory for Modern Silk (NESLab), College of Textile and Clothing Engineering, Soochow University Suzhou China

## Abstract

Cu-containing activated carbon (eco-catalyst, Cu/HMPC, where ‘C’ defines ‘carbon’) was derived from a metal-hyperaccumulating mustard plant (HMP) by a simple chemical activation method. Transmission electron microscopy/selected area diffraction (HRTEM/SAED) results revealed that the Cu/HMPC has mainly three types of morphology [sheet-like morphology (2D), hollow-spheres (3D) and needle-like structures (1D)] which are interconnected. HRTEM-SAED, Raman and X-ray photoelectron spectroscopy (XPS) results confirmed the existence of Cu oxide species in Cu/HMPC. Content of Cu in Cu/HMPC was determined to be 1.03 wt%. The quality of graphitization in Cu/HMPC was discussed by using Raman and XRD results. The BET surface area of Cu/HMPC was determined to be 620.8 m^2^ g^−1^. The Cu/HMPC actively transformed a wide range of amines to imines under very mild reaction conditions. The catalyst Cu/HMPC gave products in excellent yields (98–61%) with very high TON/TOF values (1512/339–833/35 h^−1^). To the best of our knowledge, this is the most efficient Cu-based heterogeneous eco-catalyst for the synthesis of imines among those reported to date. The Cu can be recovered from used Cu/HMPC by a simple HCl treatment. Versatility, heterogeneity and reusability of Cu/HMPC were tested. A possible mechanism has been proposed.

## Abstract 2

Cu/HMPC eco-catalyst with unique interconnected 1D/2D/3D morphology was derived from metal-hyperaccumulating mustard plant for highly efficient and selective coupling reactions.

## Graphical abstract

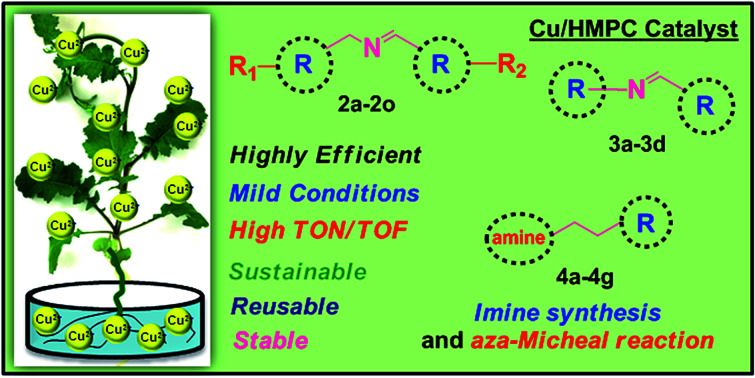

## 1. Introduction

Readily accessible C=N double bonds in imines are fundamentally important functional groups in organic synthesis which make them a highly versatile intermediate in the synthesis of various biologically active N-heterocyclic compounds.^1,2^ Catalytic aerobic oxidation of amines is one of the prime routes for the synthesis of imines.^3^ Several catalytic systems have been reported and, among them, transition metal-based catalysts (AuNPs/SBA-NH_2_,^4^ MOF-253,^5^ Cs/MnO_*x*_,^6^ Cu(0),^7^ CuCl,^8^ TiO_2_-degussa P25,^9^ α-MnO_2_,^10^ NHPI/Fe(BTC),^11^ CuO–CeO_2_,^12^ Pc-Ludox-8,^13^ AuONT-THAP,^14^ CeO_2_–MoO_3_/SiO_2_,^15^ and V^16^) exhibited a superior activity. Particularly, Cu-based heterogeneous catalytic systems are often found to be efficient and cost-effective.^7,8,13^ Recently, Al-Hmoud *et al.*,^12^ prepared a CuO–CeO_2_ catalyst and employed it for self coupling of amines including benzylamine. They found that the CuO–CeO_2_ catalyst is highly efficient and reusable. Similarly, Cu(0) nanoparticles were used for aerobic oxidative synthesis of imines from amines under solvent-free conditions.^7^ Our group has recently reported various carbon nanocomposites including CuO/MWCNT^17^ and GNS/CuO^18^ for catalytic organic transformations. Later, metal-free catalysts such as graphite oxide and biomass derived activated carbons (ACs) were also proposed for the imine synthesis.^19,20^ In spite of the results, most of the catalytic systems including Cu-based systems have often suffered from harsh reaction conditions such as high temperature, long reaction time, strong base and high loading of catalysts. Similarly, the photocatalytic systems require high energy UV-light sources.^9^ Hence, the development of a highly efficient Cu-based catalytic system under mild reaction conditions constitutes a demanding goal.

Recently, mining operations in various chemical industries have also led the accumulation of trace elements in the environment, particularly, in soil. Approximately ∼22 000 tons of Cd, ∼939 000 tons of Cu, ∼783 000 tons of Pb and ∼1 350 000 tons of Zn has been released to the environment in the year 1950–2000.^21^ Restoration of the polluted sites has become a challenging task. Various techniques have been developed to remediate the contaminated soils.^10,22^ Among them phytoremediation is an efficient and cost-effective solution. In general, phytoremediation is a mining-like process in which the metal ion can be absorbed by particular metal-hyper accumulating plants and stored into their tissues and cells.^23^ However, the metal-polluted hyper accumulating plants are difficult to be disposed. Moreover, we cannot even feed these plants to the animals too. Recently, Grison's group^24–28^ has developed a new method to extract metal species from the metal-hyperaccumulating plants. Unfortunately, the method requires a huge volume of acid solution which may cause secondary environmental pollution. Moreover, the extracted metal species needed further workout to purify it. Very recently, various biomass materials derived from plants and animals have been transformed into activated carbon (ACs) *via* simple chemical activation.^29,30^ In fact the porous texture can be readily tailored to provide high surface area. Chen and co-workers^20^ have prepared mesoporous carbon derived from vitamin B_12_ and employed as bifunctional eco-catalyst for imine formation. However, the catalytic system requires high temperature and O_2_ atmosphere. We presume that transformation of Cu-polluted plants into ACs would be a better choice as an eco-catalyst for the imine synthesis.

*Brassica juncea* L. is known as accumulator for heavy metals, and has already been used successfully for the phytoextraction of Cu from abandoned mine soils.^31^ In the present work, Cu-polluted *Brassica juncea* L. was used to prepare eco-catalyst (Cu/HMPC) by simple chemical activation. The physical and chemical properties of Cu/HMPC were characterized by HRTEM, EDX, SEM, Raman, BET, XRD and XPS techniques. The Cu/HMPC was employed for the synthesis of imines from amines. Heterogeneity, reusability, stability and versatility of Cu/HMPC were tested. Finally, Cu was extracted from used Cu/HMPC by using mild HCl.

## 2. Materials and methods

### 2.1. Materials and characterization

Seeds of *Brassica juncea* L. were collected from South India. CuCl_2_·2H_2_O was purchased from Wako Pure Chemicals and used as Cu source. All chemicals were purchased from Sigma-Aldrich or Wako Pure Chemicals and used without further purification.

Scanning electron micrographs (SEM) and corresponding energy dispersive spectrum (EDS) of eco-catalyst (Cu/HMPC) were obtained using Hitachi 3000H SEM. High resolution transmission electron microscopy (HRTEM) was performed on a JEOL JEM-2100F with accelerating voltage of 200 kV. HRTEM equipped with selected area electron diffraction (SAED, Tecnai G2 F20 S-TWIN) was operated at 300 kV. X-ray photoelectron spectrum (XPS, Kratos Axis-Ultra DLD, Kratos Analytical Ltd., Japan) was recorded to confirm the chemical state of elements presented in Cu/HMPC. Raman spectra were recorded on a Hololab 5000 (Kaiser Optical Systems Inc., USA) with a laser wavelength of 514 nm. Wide angle X-ray diffraction patterns (XRD) were obtained using Rotaflex RTP300 Diffractometer (Rigaku Co., Japan) with nickel-filtered Cu Kα radiation. The accelerating voltage and applied current were 40 kV and 200 mA, respectively. Nitrogen adsorption–desorption analysis was conducted by using TriStar 3000 (Micromeritics, USA) at 77 K. The specific surface area of Cu/HMPC was calculated by the Brunauer–Emmett–Teller (BET) method. Catalytic products were determined by gas chromatograph (GC, Shimadzu GC-2014). The GC instrument was equipped with 5% diphenyl and 95% dimethylsiloxane, a Restek-5 capillary column (0.32 mm dia, 60 m length) and a flame ionization detector (FID). N_2_ was used as a carrier gas. Both reactants and products (dissolved in ethyl acetate) were analyzed by GC. The initial column temperature was increased from 60 to 150 °C at the rate of 10 °C min^−1^ and then to 220 °C at the rate of 40 °C min^−1^. The temperatures of the FID and injection port were kept constant at 150 and 250 °C, respectively. ^13^C NMR and ^1^H NMR spectra were recorded on Bruker spectrometer operating at 100 MHz and 400 MHz, respectively. Dimethyl sulfoxide (DMSO) was used as a solvent and tetramethylsilane (TMS) was employed as an internal reference. Thermo Finnigan (Austin, Texas, USA) FOCUS DSQ (dual stage quadrupole) mass spectrometer interfaced with Finnigan FOCUS gas chromatograph (GC-MS) was performed for catalytic products. Inductively coupled plasma-mass spectrometer (ICP-MS, 7500CS, Agilent) was used to determine the factual metal loading in Cu/HMPC before and after use.

### 2.2. Cultivation of *Brassica juncea* L. (Cu/HMP)

Instead of collecting the *Brassica juncea* L. from Cu-polluted area, the plant [with Cu (Cu/HMP) and without Cu (HMP)] was cultivated in our lab. Cu is one of the catalysts for the growth of particular enzymes in HMPs which is beneficial for the growth of plants as well.^32^ Initially, the *Brassica juncea* L. seeds were sterilized with 0.5% KMnO_4_ for 15 min, then washed several times with distilled water and germinated on culture dish for 3 days at 20 °C. After germination, the young seedlings were incubated at 25 °C under white fluorescent light for a photoperiod of 12 h. A 1/4-strength modified Hoagland nutrient solution was used (see ESI† for more details about the ingredients of Hoagland nutrient solution). After few days, the nutrient solution was replaced with 1/4-strength modified Hoagland solution contained 10 μM Cu^2+^ (as CuCl_2_·2H_2_O) (we have named the plant as Cu/HMP). On other hand, some of the plants were cultured using Cu-free 1/4-strength modified Hoagland solution (we have named the plant as HMP). Growth-culture solutions were changed every three days. After thirty days, the plants were harvested and washed with deionized water to remove adhered solution and dried at 60 °C (see Fig. S1 and S2 in ESI†).

### 2.3. Preparation of HMPC and Cu/HMPC

The cultivated HMP and Cu/HMP plants were dried at 60 °C for 24 h. Then, pre-carbonization was carried out for both Cu/HMP and HMP under air atmosphere at 300 °C (heating rate of 1 °C min^−1^) for 1 h and milled separately into fine powders. Subsequently, the pre-carbonized samples were mixed with NaOH (C/NaOH = 1 : 2 ratio) using mortar and pestle until the homogeneous mixture was obtained. The mixture was further calcinated under N_2_ atmosphere at 600 °C at a heating rate of 5 °C min^−1^ for 2 h. Finally, the resultant powders were washed well with mild HCl to obtain HMPC and Cu/HMPC. Similarly, *Brassica juncea* L. plants collected from different places (Cu-mining area and agricultural area) in India were also utilized as sources materials for the preparation of catalysts (Cu/HMPC-1 and HMPC-1) (see ESI† for more details).

### 2.4. Procedure for self coupling of amines

In a typical procedure, 10 mg of Cu/HMPC (Cu 0.162 mol%) and 5 mmol of benzylamine (1a) were introduced into a glass tube and magnetically stirred under open air atmosphere at 80 °C for 4.5 h. The reaction was monitored by TLC and GC. After completion of the reaction, the Cu/HMPC was separated by centrifugation. The coupled products and unconverted reactants were analyzed by GC. The separated Cu/HMPC was washed well with diethyl ether, dried at 60 °C for 24 h and reused. Yield of the catalytic product, conversion and selectivity were calculated by using the eqn (1)–(3), respectively.

1 GC yield (%) = % of product formed

2 GC conversion (%) = 100 − % of reactant remains

3 Selectivity (%) = 100 − (conversion − yield)

After removing Cu/HMPC, the reaction mixture was diluted with ethyl acetate (∼5 mL) and dried over anhydrous MgSO_4_. The ethyl acetate was evaporated using rotary evaporator to obtain the catalytic products. Finally, the catalytic products were isolated using silica column using the mixture of ethyl acetate and hexane as an eluent (Tables 2–4). ^1^H and ^13^C NMR spectra and MS (GC) *m*/*z* spectrum were taken for the catalytic products.

#### *N*-Benzylidene-1-phenylmethanamine (Table 2, entry 2a)

Yellow liquid; ^1^H NMR (DMSO-d_6_, 400 MHz): *δ* (ppm) 8.48 (s, 1H), 7.65–7.84 (m, 2H), 7.40–7.56 (m, 3H), 7.22–7.40 (m, 5H), 4.80 (s, 2H); ^13^C NMR (DMSO-d_6_, 100 MHz): *δ* (ppm) 160.9, 140.0, 136.5, 131.1, 129.0, 128.7, 128.4, 128.3, 127.1, 64.4; GC-MS (*m*/*z*): 195.6.

#### *N*-(4-Methylbenzylidene)-1-(4-tolyl)methanamine (Table 2, entry 2b)

White solid, ^1^H NMR (DMSO-d_6_, 400 MHz): *δ* (ppm) 8.41 (s, 1H), 7.66 (d, 2H), 7.20–7.14 (m, 6H), 4.70 (s, 2H), 2.33 (s, 3H), 2.28 (s, 3H); ^13^C NMR (DMSO-d_6_, 101 MHz): *δ* (ppm) 161.6, 140.9, 136.1, 133.9, 129.6, 129.3, 128.3, 64.1, 21.4, 21.0; GC-MS (*m*/*z*): 223.1.

#### *N*-(3-Methylbenzyl)-1-(3-tolyl)methanimine (Table 2, entry 2c)

Yellowish oil; ^1^H NMR (DMSO-d_6_, 400 MHz): *δ* (ppm) 8.42 (s, 1H), 7.57–7.61 (m, 2H), 7.05–7.33 (m, 6H), 4.72 (s, 2H), 2.33 (s, 3H), 2.29 (s, 3H); ^13^C NMR (DMSO-d_6_, 101 MHz): *δ* (ppm) 162.0, 139.9, 138.2, 137.7, 136.5, 131.7, 128.9, 128.9, 128.7, 128.6, 127.7, 125.8, 125.4, 64.5, 30.9, 21.4, 21.2; GC-MS (*m*/*z*): 223.1.

#### *N*-(2-Methylbenzylidene)-2-methylbenzylamine (Table 2, entry 2d)

Yellowish oil, ^1^H NMR (DMSO-d_6_, 400 MHz): *δ* (ppm) 8.76 (s, 1H), 7.83–7.85 (d, 2H), 7.14–7.36 (m, 6H), 4.79 (s, 2H), 2.36 (s, 3H), 2.34 (s, 3H). ^13^C NMR (DMSO-d_6_, 101 MHz): *δ* (ppm) 160.9, 138.4, 137.9, 136.2, 134.3, 131.2, 130.5, 130.2, 128.4, 127.7, 127.1, 126.4, 126.2, 62.9, 21.2, 19.4, 19.2; GC-MS (*m*/*z*): 223.1.

#### *N*-(4-Methoxybenzylidene)-1-(4-methoxyphenyl)methanamine (Table 2, entry 2e)

Yellow oil, ^1^H NMR (DMSO-d_6_, 400 MHz): *δ* (ppm) 8.35 (s, 1H), 7.75–7.72 (m, 2H), 7.26–6.89 (m, 6H), 4.66 (s, 2H), 3.78 (s, 6H); ^13^C NMR (DMSO-d_6_, 100 MHz): *δ* (ppm) 191.5, 161.6, 160.8, 158.6, 145.5, 132.2, 132.1, 129.9, 129.4, 129.0, 114.8, 114.4, 114.1, 113.7, 63.9, 55.9, 55.5, 55.3.

#### *N*-(4-Fluorobenzylidene)-1-(4-fluorophenyl)methanamine (Table 2, entry 2f)

Yellow oil; ^1^H NMR (DMSO-d_6_, 400 MHz): *δ* (ppm) 8.43 (s, 1H), 7.86–7.80 (m, 2H), 7.37–7.35 (m, 2H), 7.24–7.11 (m, 4H), 4.72 (s, 2H); ^13^C NMR (DMSO-d_6_, 100 MHz): *δ* (ppm) 165.3, 162.8, 162.8, 160.8, 160.4, 136.1, 136.0, 133.0, 132.9, 130.5, 130.5, 130.0, 129.9, 116.0, 115.8, 115.4, 115.2, 63.4; GC-MS (*m*/*z*): 231.1.

#### *N*-(4-Chlorobenzylidene)-1-(4-chlorophenyl)methanamine (Table 2, entry 2g)

White solid, ^1^H NMR (DMSO-d_6_, 400 MHz): *δ* (ppm) 8.43 (s, 1H), 7.75–7.78 (d, 2H), 7.47–7.51 (d, 2H), 7.31–7.35 (m, 4H), 4.73 (m, 2H); ^13^C NMR (DMSO-d_6_, 100 MHz): *δ* (ppm) 161.2, 138.8, 135.9, 135.1, 131.9, 129.9, 129.9, 129.1, 128.6, 63.3; GC-MS (*m*/*z*): 263.0.

#### *N*-(Thiophen-2-yl)-1-(thiophen-2-yl)methanamine (Table 2, entry 2i)

Reddish brown oil; ^1^H NMR (DMSO-d_6_, 400 MHz): *δ* (ppm) 8.55 (s, 1H), 7.67–7.65 (d, 2H), 7.50–7.40 (m, 2H), 7.15–7.00 (m, 2H), 4.91 (s, 2H); ^13^C NMR (DMSO-d_6_, 100 MHz): *δ* (ppm) 156.1, 142.7, 142.3, 132.2, 130.2, 128.2, 127.3, 127.1, 125.4, 58.3; GC-MS (*m*/*z*): 207.0.

#### *N*-Pentylidenepentan-1-amine (Table 2, entry 2l)

Reddish brown oil; ^1^H NMR (DMSO-d_6_, 400 MHz): *δ* (ppm) 7.60–7.79 (m, 1H), 1.77–1.24 (m, 14H), 0.88–0.84 (m, 6H); ^13^C NMR (DMSO-d_6_, 100 MHz): *δ* (ppm) 162.76, 62.4, 31.61, 29.45, 28.79, 26.78, 22.40, 22.31, 14.27, 14.01.

#### *N*-Hexylidenehexan-1-amine (Table 2, entry 2m)

Pale yellow oil; ^1^H NMR (DMSO-d_6_, 400 MHz): *δ* (ppm) 7.79 (m, 1H), 1.52–1.28 (m, 16H), 0.91–0.87 (m, 6H); ^13^C NMR (DMSO-d_6_, 100 MHz): *δ* (ppm) 161.14, 62.34, 31.37, 31.32, 31.30, 29.34, 26.54, 25.40, 22.42, 22.24, 14.18.

#### 3,4-Dihydroisoquinoline (Table 2, entry 2n)

Pale orange oil; ^1^H NMR (DMSO-d_6_, 400 MHz): *δ* (ppm) 8.33 (s, 1H), 7.40–7.19 (m, 4H), 3.66–3.61 (m, 2H), 2.68–2.64 (m, 2H); ^13^C NMR (DMSO-d_6_, 100 MHz): *δ* (ppm) 159.77, 141.3, 131.29, 130.89, 127.38, 126.39, 47.15, 24.68.

### 2.5. Procedure for cross coupling of amines with aniline

A 10 mg of Cu/HMPC (Cu 0.162 mol%), 1 mmol of benzylamine and 4 mmol of aniline were magnetically stirred under open air atmosphere at 80 °C for 4.5 h. The Cu/HMPC was separated by centrifugation and the centrifugate was analyzed by GC. Yield of the product, conversion and selectivity were calculated by using the eqn (1)–(3), respectively. ^1^H and ^13^C NMR spectra and MS (GC) *m*/*z* spectrum were recorded for the catalytic products.

#### *N*-Benzylideneaniline (Table 3, entry 3a)

Yellow solids, ^1^H NMR (DMSO-d_6_, 400 MHz): *δ* (ppm) 8.61 (s, 1H), 7.97–7.94 (m, 2H), 7.54–7.23 (m, 8H); ^13^C NMR (DMSO-d_6_, 101 MHz): *δ* (ppm) 161.0, 151.8, 136.4, 131.8, 129.6, 129.2, 129.0, 126.3, 121.3; GC-MS (*m*/*z*): 180.8.

#### *N*-(4-Methylbenzylidene)aniline (Table 3, entry 3b)

Yellow oil, ^1^H NMR (CDCl_3_, 400 MHz): *δ* (ppm) 8.32 (s, 1H), 7.66–7.64 (m, 2H), 7.23–7.11 (m, 7H), 2.36 (s, 3H); ^13^C NMR (CDCl_3_, 101 MHz): *δ* (ppm) 162.2, 152.66, 141.43, 133.99, 130.10, 130.00, 129.73, 129.58, 128.69, 128.39, 126.18, 121.31, 21.91.

#### *N*-(3-Methylbenzylidene)aniline (Table 3, entry 3c)

Yellow oil, ^1^H NMR (CDCl_3_, 400 MHz): *δ* (ppm) 8.39 (s, 1H), 7.74–7.72 (m, 2H), 7.39–7.18 (m, 7H), 2.39 (s, 3H); ^13^C NMR (CDCl_3_, 101 MHz): *δ* (ppm) 161.13, 152.60, 138.97, 132.71, 129.90, 129.53, 129.45, 126.91, 126.34, 121.34, 21.75.

#### *N*-(Thiophen-2-ylmethylene)aniline (Table 3, entry 3d)

Yellow oil, ^1^H NMR (DMSO-d_6_, 400 MHz): *δ* (ppm) 8.78 (s, 1H), 7.82–7.69 (m, 2H), 7.43–7.21 (m, 6H); ^13^C NMR (DMSO-d_6_, 101 MHz): *δ* (ppm) 154.19, 151.18, 142.84, 133.97, 131.49, 129.59, 128.57, 126.37, 121.40.

### 2.6. Procedure for aza-Michael reaction

A mixture of amines (1 or 2 mmol), α,β-unsaturated compounds (2 mmol), and Cu/HMPC (5–10 mg) was stirred under open air atmosphere at 27 °C for 15 min. Ethyl acetate was used to extract the aza-adducts. From GC analysis, yield, conversion and selectivity were calculated.

#### Methyl 3-(piperidin-1-yl)propanoate (Table 5, entry 4a)

Yellow liquid: ^1^H NMR (DMSO-d_6_, 400 MHz): *δ* (ppm) 3.52 (s, 3H), 2.48–2.46 (m, 2H), 2.37–2.24 (m, 6H), 1.45–1.29 (m, 6H); ^13^C NMR (DMSO-d_6_, 101 MHz): *δ* (ppm) 172.2, 54.2, 54.1, 51.0, 32.0, 26.0, 24.4; GC-MS *m*/*z*: 171.1.

#### 3-(Piperidin-1-yl)propanenitrile (Table 5, entry 4b)

Yellow liquid: ^1^H NMR (DMSO-d_6_, 400 MHz): *δ* (ppm) 2.56–2.60 (m, 4H), 2.38–2.40 (m, 4H), 1.56–1.38 (m, 6H); ^13^C NMR (DMSO-d_6_, 101 MHz): *δ* (ppm) 119.6, 54.0, 53.8, 25.9, 24.3, 15.5; GC-MS *m*/*z*: 138.1.

#### Methyl 3-morpholinopropanoate (Table 5, entry 4c)

Yellow liquid: ^1^H NMR (DMSO-d_6_, 400 MHz): *δ* (ppm) 3.61–3.54 (m, 2H), 2.59–2.57 (m, 7H), 2.49–2.35 (m, 6H); ^13^C NMR (DMSO-d_6_, 101 MHz): *δ* (ppm) 172.4, 66.5, 53.9, 53.4, 51.3, 31.7; GC-MS *m*/*z*: 175.1.

#### 3,3′-(*N*-Benzylimino)dipropionic acid dimethyl ester (Table 5, entry 4d)

Yellow solid: ^1^H NMR (DMSO-d_6_, 400 MHz): *δ* (ppm) 7.32–7.22 (m, 5H), 3.69 (s, 9H), 3.59–3.55 (m, 4H), 2.75–2.67 (m, 2H), 2.46–2.43 (m, 4H); ^13^C NMR (DMSO-d_6_, 101 MHz): *δ* (ppm) 172.9, 172.7, 141.1, 128.8, 128.4, 128.2, 127.1, 126.8, 57.7, 53.0, 51.5, 48.9, 44.6, 34.7, 32.2, 31.0; GC-MS *m*/*z*: 281.0.

#### 3,3′-(Benzylazanediyl)dipropanenitrile (Table 5, entry 4e)

^1^H NMR (DMSO-d_6_, 400 MHz): *δ* (ppm) 7.35–7.23 (m, 5H), 3.72 (s, 2H), 2.74–2.72 (m, 4H), 2.59–2.58 (m, 4H); ^13^C NMR (DMSO-d_6_, 101 MHz): *δ* (ppm) 140.8, 128.5, 128.2, 127.0, 120.4, 52.5, 44.4, 31.0, 18.1.

#### 3-(Pyrrolidin-1-yl)propanenitrile (Table 5, entry 4f)

^1^H NMR (DMSO-d_6_, 400 MHz): *δ* (ppm) 2.64 (s, 2H), 2.48–2.46 (m, 6H), 1.72–169 (m, 4H); ^13^C NMR (DMSO-d_6_, 101 MHz): *δ* (ppm) 120.2, 53.5, 51.0, 23.5, 17.2. GC-MS *m*/*z*: 124.0.

#### 3-(4-Phenylpiperazin-1-yl)propanenitrile (Table 5, entry 4g)

^1^H NMR (DMSO-d_6_, 400 MHz): *δ* (ppm) 7.26–7.22 (m, 2H), 6.94–6.92 (m, 1H), 6.82–6.80 (m, 2H), 3.14–3.11 (m, 8H), 2.65–2.55 (m, 4H); ^13^C NMR (DMSO-d_6_, 101 MHz): *δ* (ppm) 151.3, 129.3, 120.2, 119.2, 115.8, 115.6, 53.1, 52.5, 49.8, 48.5, 46.1, 15.4. GC-MS *m*/*z*: 215.1.

### 2.7. Recovery of HMPC and Cu from Cu/HMPC

The Cu/HMPC was separated out from mixture by simple centrifugation and washed with diethyl ether followed by drying under air atmosphere at 60 °C. A 100 mg of used Cu/HMPC was dispersed in 10 mL of 1.0 M HCl and magnetically stirred under open air atmosphere at 60 °C for 5 h. The reaction mixture was then vacuum-filtered through filter paper and the filtrate was concentrated at 100 °C. The obtained solid (recovered Cu) was washed several times with deionized water and analyzed.

## 3. Results and discussion

### 3.1. Physicochemical properties of Cu/HMPC

**Fig. 1:**
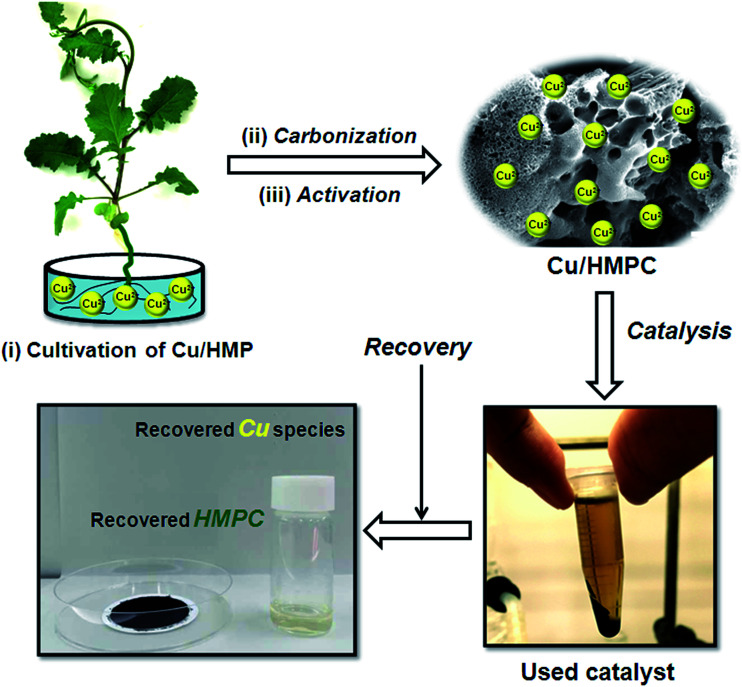
Schematic diagram for the preparation and catalytic process of Cu/HMPC.

**Fig. 2:**
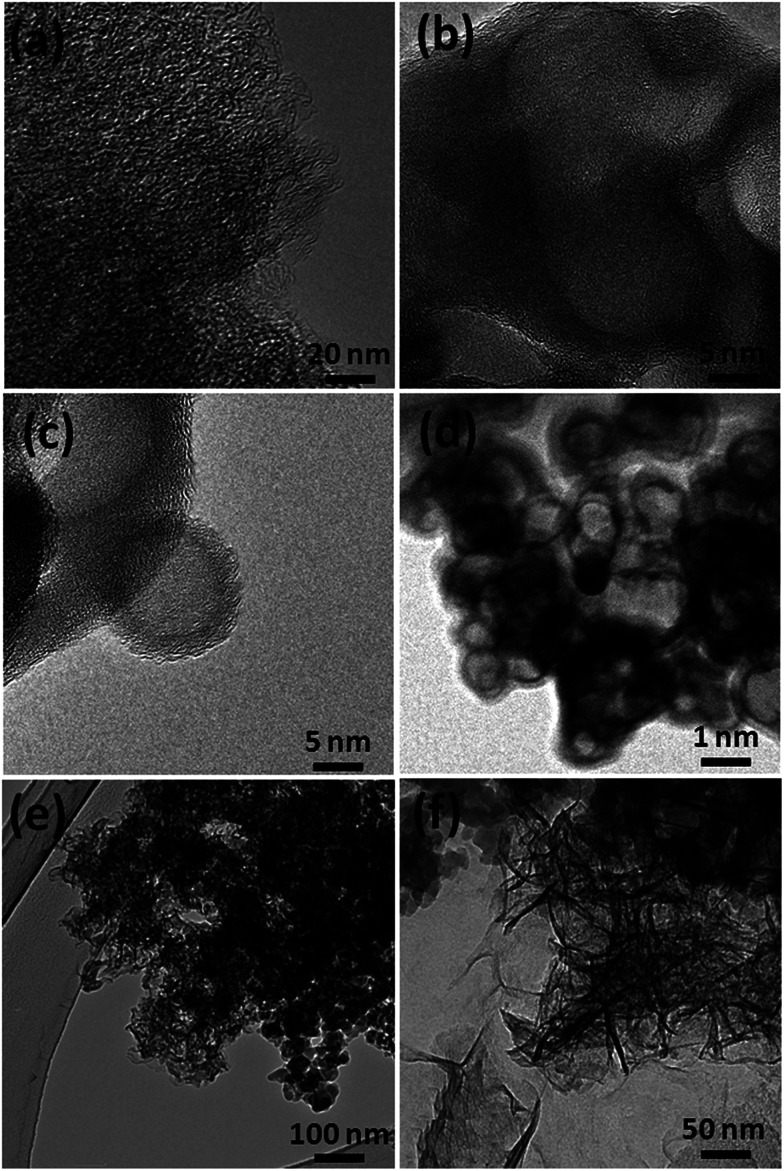
HRTEM images of Cu/HMPC.

**Fig. 3:**
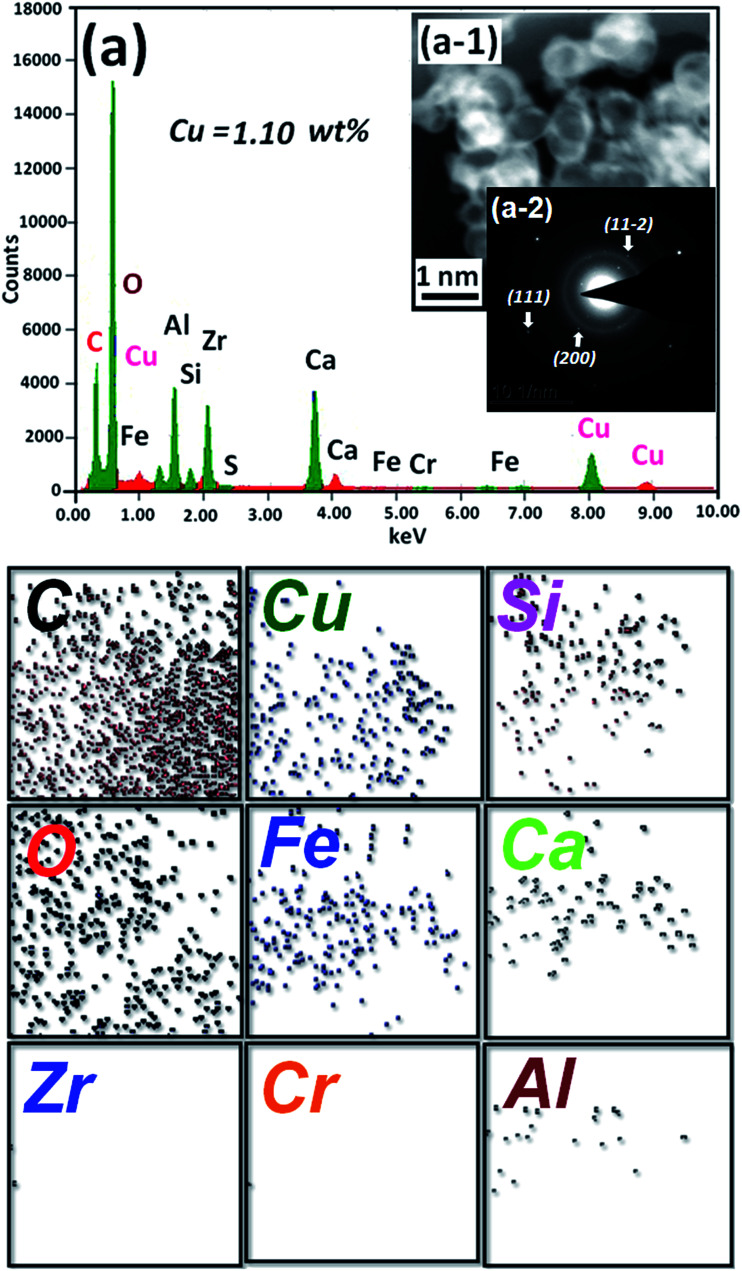
(a) EDX spectrum, (a-1) HRTEM image, (a-2) SAED pattern of Cu/HMPC and representative elemental mapping of C, Cu, Si, O, Fe, Ca, Zr, Cr and Al.

**Fig. 4:**
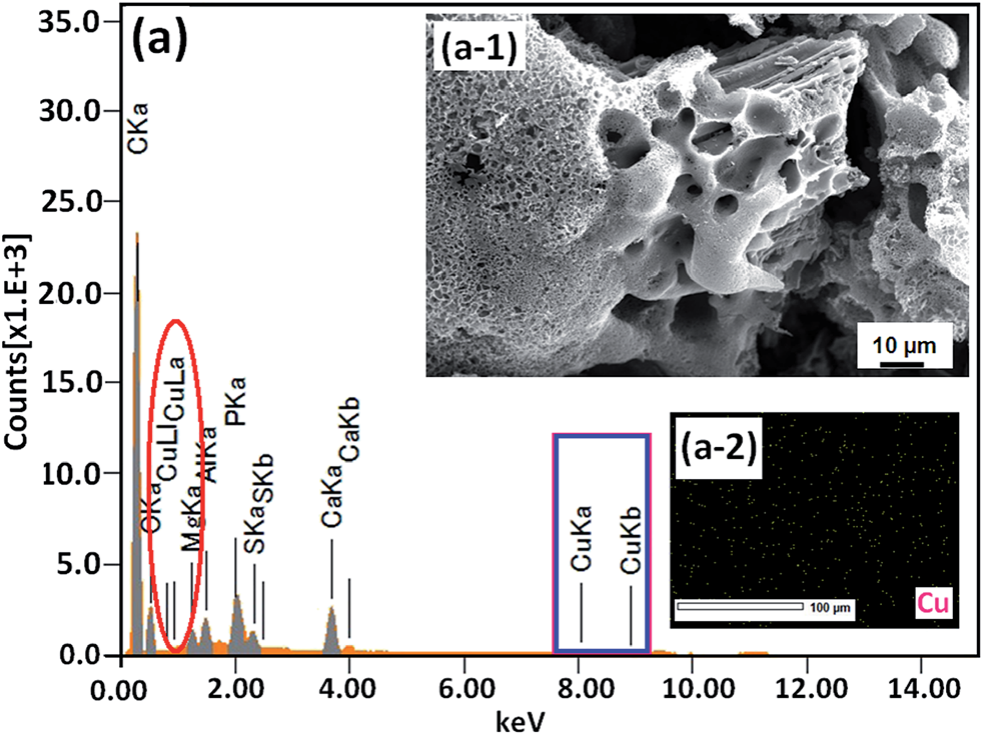
(a) EDX spectrum, (a-1) SEM image and (a-2) Cu mapping of Cu/HMPC.

Recently, carbon materials such as graphene, carbon nanotubes and fullerenes are found to be excellent carbocatalysts for oxidation reactions.^33^ Although the materials are expensive and difficult to be prepared, still they have attracted considerable attention due to their unique properties such as high surface area (BET surface area of ≤500–1500 m^2^ g^−1^), chemical stability, unique dimensions (1D, 2D and 3D), and higher order of dispersion and so on.^34,35^ The present Cu/HMPC has similar type of the properties compare to carbon materials. Fig. 1 shows the schematic diagram for the preparation of Cu/HMPC. In order to investigate the structural morphology in detail, HR-TEM images were taken for HMPC and Cu/HMPC; the images are shown in Fig. 2 and S3–S8 in ESI.† Typically ten different areas were chosen to capture the HR-TEM images. We found that the Cu/HMPC catalyst has certainly accommodated three types of morphology which are interconnected each other; (a) sheets-like morphology (2D), (b) hollow-spheres (3D) and (c) needle-like structure (1D). In addition, carbon nanoparticle having 15–25 nm in size could also be seen in the Cu/HMPC (Fig. S4b in ESI†). The HRTEM images [Fig. 2a and b] confirmed the sheet-like morphology of the catalyst. The sheets were continuous, wrinkled, transparent and irregular ultra thin having size ranging from 5 nm to 20 nm. Also, the pores channels can be clearly seen on the surface of the Cu/HMPC. The existence of carbon hollow-spheres with diameter of about ∼20 nm was confirmed from the magnified HRTEM images of Cu/HMPC [Fig. 2c and d]. In its carbon hollow-spheres arrangement, the graphitic flasks are not closed shells and the unclosed interlayer carbon flasks offer reactive ‘dangling bonds’ that are proposed to enhance surface reactions, suggesting Cu/HMPC as good material for catalytic applications. In addition, we could see needle-like morphology of Cu/HMPC (1D). The mean diameter and length of the needles are found to be ∼6 nm and ∼110 nm, respectively. More interestingly, all the three structures (1D/2D/3D) are interconnected each other (Fig. S4a in ESI†). Refer Fig. S3–S5 in ESI† for more details. Similar to Cu/HMPC, the pure HMPC was also demonstrated the unique morphology which includes sheets-like morphology (2D), needle-like structure (1D) and spherical carbon nanoparticles. However, we could not notice any hollow-spheres arrangement in the HMPC morphology. Fig. 3 shows HR-TEM images, SAED pattern, EDS and corresponding elemental mappings of Cu/HMPC. As expected, the results confirmed the existence of Cu including C, Si, O, Fe, Ca, and Al. The source of this trace amount of Mg, Ca and Al is nutrition solution. The content of Cu was determined to be 1.07. ICP-MS analysis was also carried out for Cu/HMPC. The result showed that the content of Cu in Cu/HMPC is 1.03 which is very close to the EDS results. The SAED pattern [Fig. 3(a-2)] depicted three major crystalline planes *d*(1 1 1) = 0.252 nm, *d*(2 0 0) = 0.231 nm, and *d*(11−2) = 0.196 nm which confirmed the existence of CuO phase (PDF card #48-1548).^36^ The homogenous dispersion of Cu and other elements was acknowledged by the mapping result (Fig. 3). The presence of more elements can contribute in synergetic effect and therefore enhance the activity of the catalysts. The SEM-EDS and corresponding elemental mapping were also carried out to cross check the Cu metal loading in Cu/HMPC (Fig. 4). As anticipated, the content of Cu was 1.10% which is almost similar to the result of TEM-EDX (1.10 wt% of Cu). The Cu mapping shows that the Cu species dispersed uniformly without any aggregation.

The formation of interconnected 1D/2D/3D morphology of Cu/HMPC is very difficult to explain. However, we assume that the nature and elemental composition of plant have a significant role on the 1D/2D/3D morphology of Cu/HMPC. Generally, the plant consists of various hard and soft parts such as leaves, stems, nerves, roots and *etc.* During chemical activation process, the way of peeling off of graphite flacks is one of the key factors for the unique morphology of ACs composites. The carbon layers in graphite stacked by very weak van der Waals forces having interaction energy of ∼2 eV nm^−2^.^37^ Typically ∼300 nN l^−1^ m^−2^ magnitude of force is obviously required to break this energy.^37^ However, precarbonized carbon derived from the hard part of the plants may be required more energy. The arrangement of carbon stacks in the pre-carbonized Cu/HMPC also a main factor. In addition, heteroatom, trace amount of metals and alkali metals presented in the precarbonized Cu/HMPC might have played significant role in the formation of this unique interconnected 1D/2D/3D morphology of Cu/HMPC. Particularly, the presence of Cu might also be the reason for the formation of hollow-spheres arrangement in the Cu/HMPC morphology which was not observed in HMPC.

**Fig. 5:**
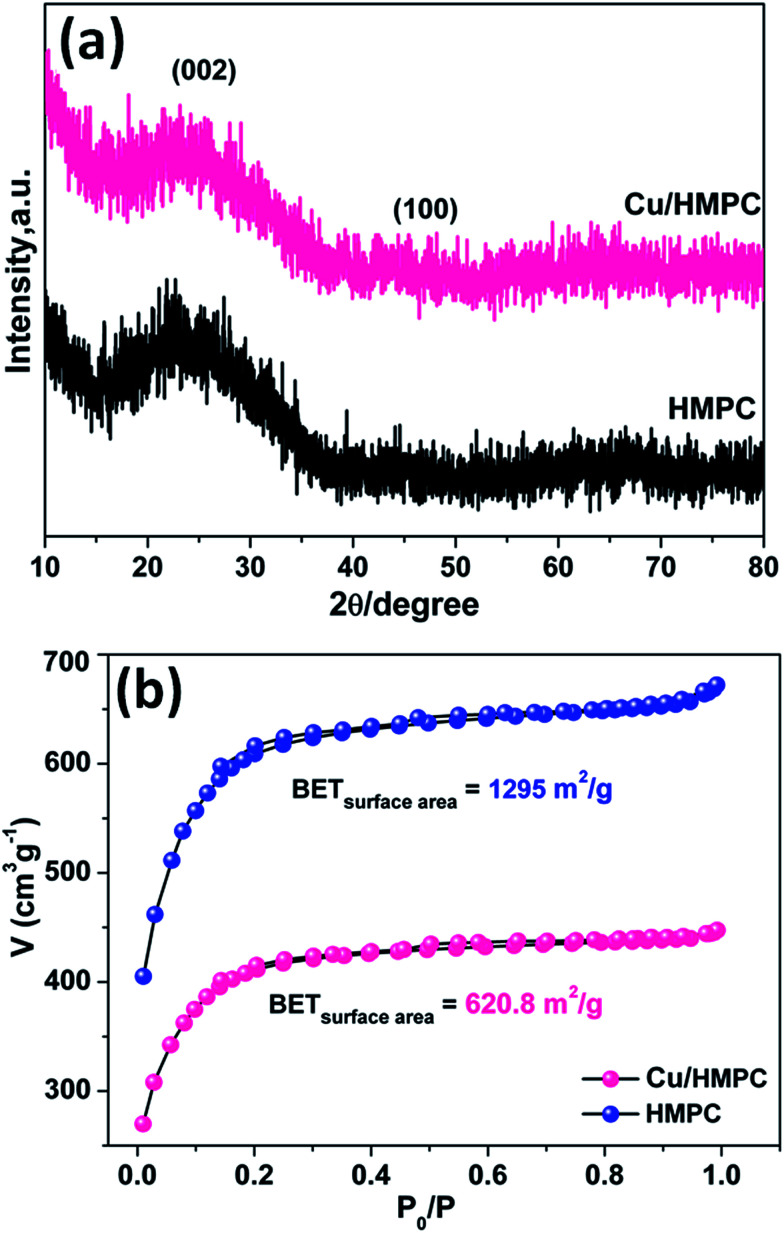
(a) XRD and (b) BET isotherms of HMPC and Cu/HMPC.

The XRD patterns and BET isotherms of Cu/HMPC and HMPC are shown in Fig. 5. It was observed that both Cu/HMPC and HMPC exhibited nearly same diffraction features with a broad diffraction peak at 2*θ* = 22.3° corresponding to (002) plane and a weak peak at 2*θ* = 43.8° corresponding to (101) plane.^38^ The peaks attributed to a well-defined graphitic stacking with higher degree of interlayer condensation of carbon. We did not see any peaks to reveal the presence of Cu and other elements such as Si, O, Fe, Ca, and Al, which were detected by TEM-EDS, HRTEM-SAED and SEM-EDS. This is probably due to the lower amount and atomic level dispersion of elements including Cu into the carbon matrix. Nitrogen adsorption–desorption isotherms and surface area of Cu/HMPC and HMPC were performed. According to IUPAC classification, both Cu/HMPC and HMPC showed typical type IV isothermal plots with hysteresis loops that indicate the existence of mesopores. The specific surface area of Cu/HMPC and HMPC was determined to be 620.8 m^2^ g^−1^ and 1295.0 m^2^ g^−1^, respectively. The data suggest that the BET surface area obviously affected by the presence of Cu in Cu/HMPC.

**Fig. 6:**
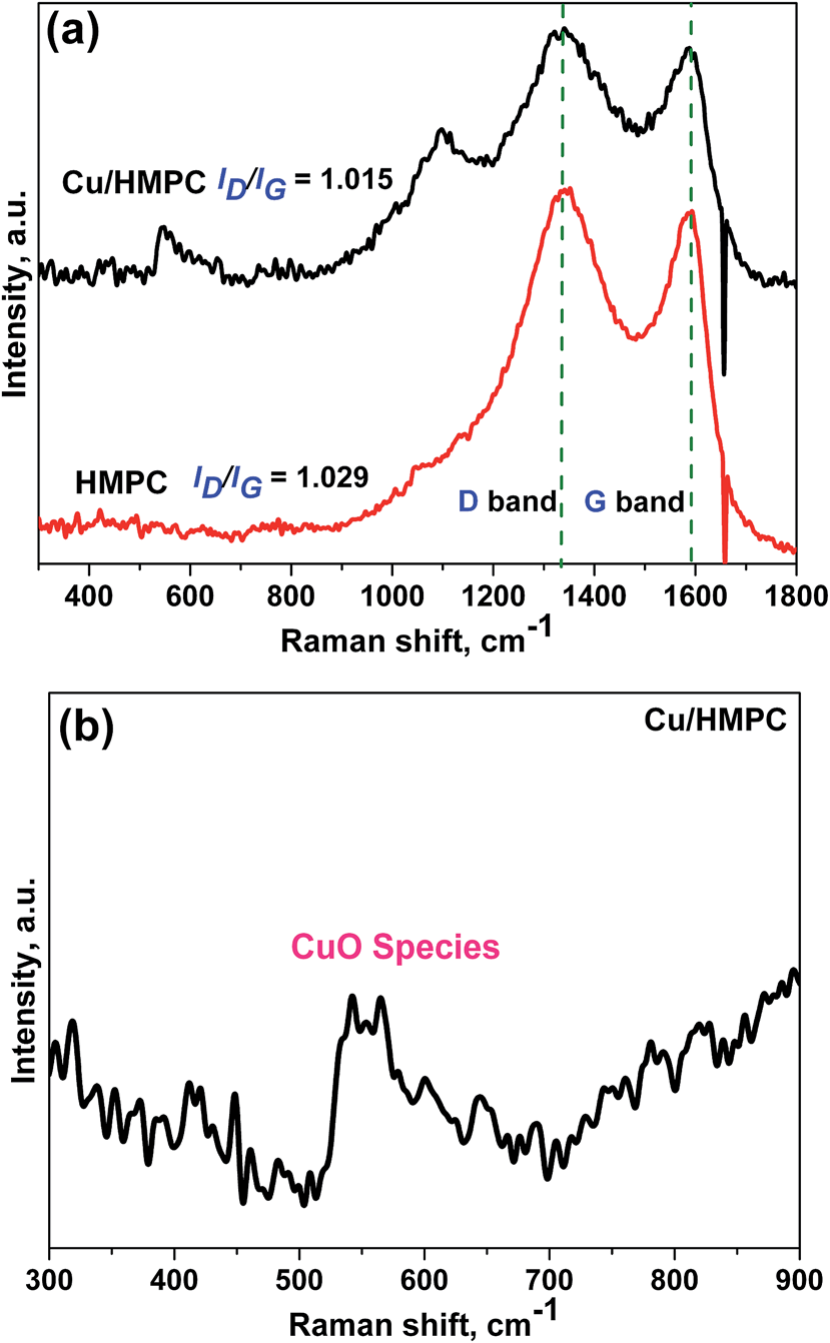
(a) Raman spectra of HMPC and Cu/HMPC. (b) Magnified Raman spectrum of Cu/HMPC.

Raman spectra were taken for Cu/HMPC and HMPC; the results are provided in Fig. 6. Two intense Raman peaks at around 1350 cm^−1^ (D band) and 1590 cm^−1^ (G band) were noticed for both Cu/HMPC and HMPC.^39^ The G band is a characteristic feature of graphitic layers, while the D band corresponds to disordered carbon or defective graphitic structures.^40^ The *I*_D_/*I*_G_ ratio of Cu/HMPC and HMPC was calculated to be 1.015 and 1.029 respectively. The high *I*_D_/*I*_G_ values indicate the existence of large amount of defects sites in the carbon matrix. The defect sites might have caused by the presence of Si, O, Fe, Ca, and Al including Cu elements in carbon matrix. Interestingly, a new and broad peak at around 550 cm^−1^ was observed which further confirmed the presence of CuO species in Cu/HMPC.^41^

**Fig. 7:**
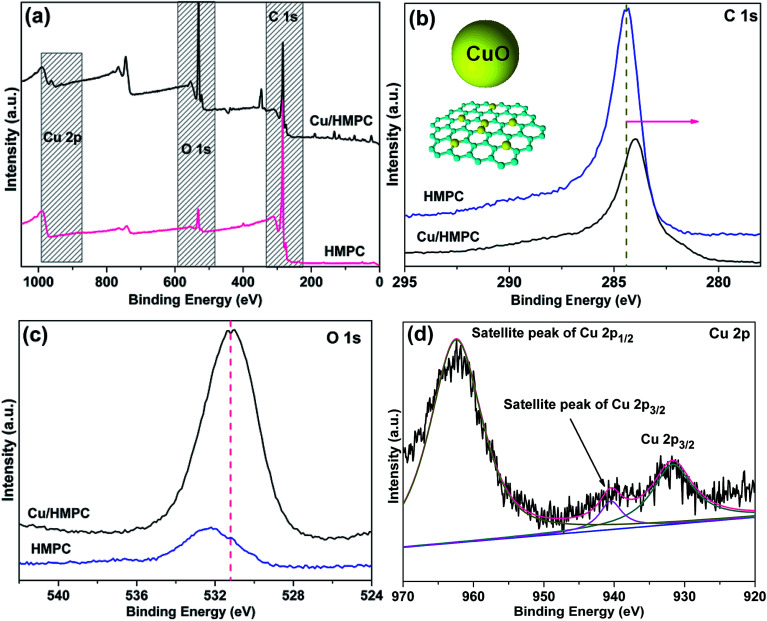
(a) XPS spectrum, (b) C 1s peaks, and (c) O 1s peaks of HMPC and Cu/HMPC. (d) Cu 2p peak of Cu/HMPC.

Chemical property of Cu/HMPC and HMPC were investigated in detail by XPS analysis. Fig. 7 shows the XPS spectrum of Cu/HMPC and HMPC. Both Cu/HMPC and HMPC displayed strong C 1s and O 1s peaks at ∼285 and ∼533 eV, respectively.^42^ It was found that presence of Cu in carbon matrix has big impact on the physicochemical property of Cu/HMPC. In comparison to HMPC, the intensity of C 1s peak at 285 eV dramatically decreased and the position of C 1s and O 1s peaks also significantly shifted toward lower binding energy (Fig. 7b and c). This is maybe due to the atomic level distribution of Cu species into the carbon matrix. Fig. 7d depicts deconvoluted Cu 2p spectrum of Cu/HMPC. Three deconvoluted peaks were noticed at 962 eV, 941 eV and 932 eV for Cu/HMPC.^43^ The obtained spectrum is unusual neither from the spectrum of Cu^+^ nor Cu^2+^, and there is no similar report can be found in the literature. However, without any doubt, the peak at ∼932 eV is a characteristic peak for Cu 2p_3/2_.^17^ We assumed that the lack of Cu 2p_1/2_ might have caused due to the existences of Cu species as chelate compound inside the carbon framework. Based on the results, we presume that the Cu species were chemically interacted with the HMPC carbon matrix. It also helps to explain why the satellite peak at ∼962 eV has stronger intensity than the main peak. The presence of two satellite peaks confirmed the +2 oxidation state of Cu species. In fact the satellite peaks attributed to 3d^9^ shell of Cu^2+^ ions.

**Fig. 8:**
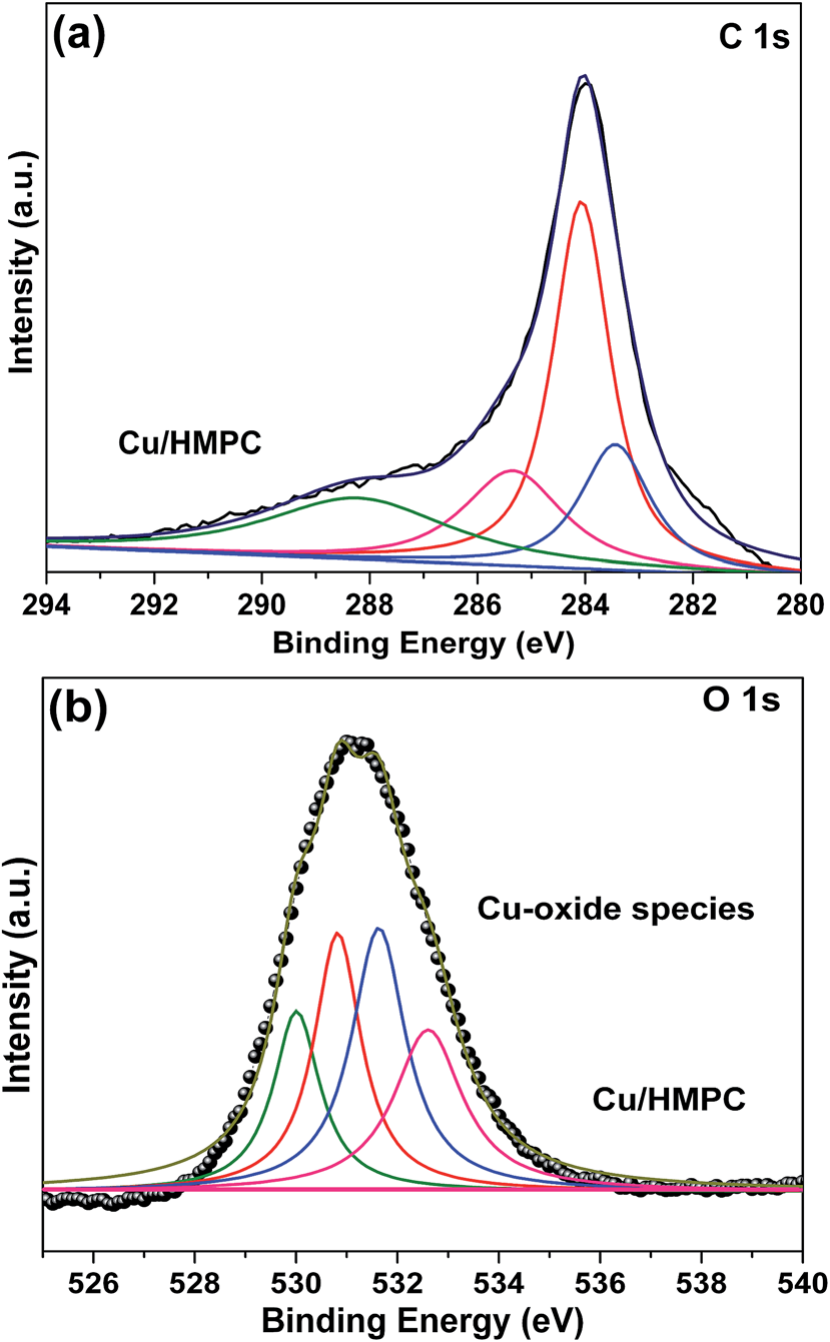
(a) C 1s and (b) O 1s XPS peaks of Cu/HMPC.

The presence of oxygen functional groups such as C–C/C=C, C–OH, C–O–C, C=O and COOH and H_2_O molecules in Cu/HMPC was investigated by fitting C 1s and O 1s XPS peaks of Cu/HMPC.^44^ The intensity of C 1s peak at 285 eV significantly improved which is due to the oxidation of activated carbon during activation process (Fig. 7c). In fact, the functional groups can greatly assist for higher dispersion of Cu/HMPC in the reaction medium. In addition, trace amount of H_2_O presented in Cu/HMPC would be very helpful in imines synthesis. The deconvoluted C 1s and O 1s XPS spectra of Cu/HMPC are presented in Fig. 8. The peak at 285 eV can be fit to peaks at 283.9, 284.7, 286.4 and 287.1 eV and thus attributed to C–C/C=C, C–OH, C–O–C and C=O groups, respectively.^45^ At O 1s XPS peak, we could fit four obvious peaks at 530.2, 530.8, 531.9, 532.3 and 533.6 eV correspond to C=O, –COOH, C–OH, –C–O–C– and H_2_O respectively.^46^ Based on the results obtained by HRTEM-EDS, SEM-EDS, Raman, XRD, XPS and BET, we found that the Cu/HMPC is a suitable catalyst for imine synthesis.

### 3.2. Optimization for reaction conditions

Self-coupling of benzylamine (1a) to *N*-benzylidene-1-phenylmethanamine (2a) was chosen as the model reaction to find out the most suitable reaction conditions. Various reaction parameters such as temperature, time, amount of Cu/HMPC, amount of reactant and atmosphere were optimized in detail and the results are summarized in Table 1. Since economical and environmentally benign conditions are highly desirable, the present Cu/HMPC system is developed under mild reaction conditions such as solvent free and open air atmosphere. No product was detected in the absence of Cu/HMPC (Table 1, entry 1). The reaction was performed with Cu/HMP before activation [Cu/HMP dried at 60 °C (Cu/HMP-60) and Cu/HMP after pre-carbonization at 300 °C] (Table 1, entries 2–4). However, the Cu/HMP showed poor catalytic activity even after long reaction time which indicates the necessity of chemical activation to achieve higher specific surface area of catalysts. Similarly, Cu-free HMPC was also tested as catalyst for this reaction. Although the surface area of HMPC (1295.0 m^2^ g^−1^) was higher compare to Cu/HMPC (620.8 m^2^ g^−1^), a very low amount of the targeted product (2a) was obtained by HMPC at different temperatures (70, 80 and 100 °C) (Table 1, entries 5–7); reveals the needfulness of Cu. To our delight, Cu/HMPC afforded the desired product (2a) in excellent yield of 98% (Table 1, entry 8). In fact, Cu/HMPC, as polymetallic catalysts, is thus highly efficient with low catalytic loadings due to the synergy phenomena between their constitutive metallic elements. Instead of Cu/HMPC, equivalent amount of CuCl_2_ was used as catalyst for the self-coupling of 1a, however, the system obtained a very low 3% of 2a (Table 1, entry 9). The amount of Cu/HMPC also played crucial role in the present catalytic system (Table 1, entries 10 and 11). It was found that the optimum amount of Cu/HMPC is 10 mg (Table 1, entry 8). The yield of desired product is decreased as the amount of Cu/HMPC is increased (Table 1, entry 11). Alike, catalyst amount, the reaction temperature has also played significant role in the present catalytic reaction (Table 1, entries 12–18). An excellent yield of 98% was obtained when the reaction was stirred at 80 °C (Table 1, entry 8). We later found that this is the mild reaction conditions developed to date for Cu-mediated self-coupling of amines. Decreasing the reaction temperature from 80 °C to 70 °C or 60 °C resulted in poor yield of the desired product (2a) even after extended reaction time (Table 1, entries 16–18). Interestingly, the Cu/HMPC gave poor yields when the reaction was performed at high temperatures (85 °C and 100 °C) (Table 1, entries 12–15). However, we noticed that the yield of 2a was slightly improved with extended reaction time (4.5 h to 11 h or 15 h) but still lower than 98% (Table 1, entries 13, and 15). In fact, the dissolved oxygen in the reaction mixture is very important. However, at high temperatures the dissolved oxygen in the reaction mixture maybe low and resulting in poor yields. Subsequently, reaction time was screened (Table 1, entries 8, 19 and 20) and found that 4.5 h is enough to obtain the maximum yield (Table 1, entry 8). In addition, low amount of 2a (15%) was obtained when the reaction was performed with 1.0 mmol of benzylamine under optimized reaction conditions (Table 1, entry 14). Under N_2_ atmosphere, the present Cu/HMPC system gave only 55% of 2a which shows the importance of O_2_ from air atmosphere (Table 1, entry 15). The Cu/HMPC was not efficient when water was used as solvent (Table 1, entry 23). For comparison, Cu/HMPC prepared by using a simple mix and heat method was also employed as catalysts for the self-coupling of 1a to 2a (Table 1, entry 24). In addition, HMPC-1 and Cu/HMPC-1 were also performed as catalyst (Table 1, entries 25–28). However, under the present mild conditions, the HMPC-1 and Cu/HMPC-1 respectively afford a low 41% and 66% yield of the desired product (Table 1, entries 27 and 28). Alike other catalytic systems,^1–8,10,11,13–16^ the HMPC-1 and Cu/HMPC-1 required high temperature as well as O_2_ atmosphere to achieve the better yield (Table 1, entries 27 and 28). Overall, under the optimized reaction conditions, the present catalytic system showed an excellent yield of 98% with 100% selectivity and very high TON/TOF values of 1512/336 h^−1^.

**Table 1:** Optimization of reaction conditions for self-coupling of benzylamine^a^

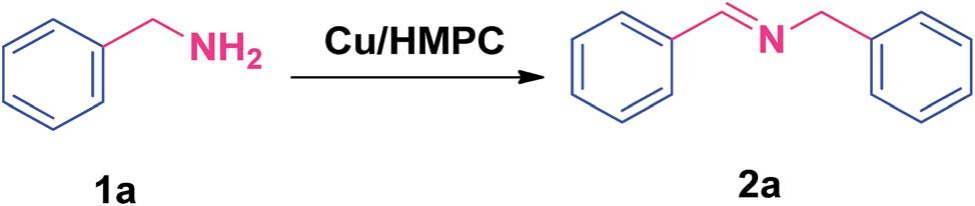						
Entry	Catalyst	Amount of catalyst (mg/Cu mol%)	Temperature (°C)	Time (h)	GC yield^b^ (%)	(TON/TOF^c^ h^−1^)
1	—	—	80	4.5	Trace	—
2	Cu/HMP-60	10/0.162	80	4.5	5	—
3	Cu/HMP-60	10/0.162	80	12	15	—
4	Cu/HMP-300	10/0.162	80	4.5	11	—
5	HMPC	10/—	70	4.5	39	—
6	HMPC	10/—	80	4.5	57	—
7	HMPC	10/—	100	4.5	55	—
**8**	**Cu/HMPC**	**10/0.162**	**80**	**4.5**	**98**	**1512/336**
9	CuCl_2_	—/0.162	80	4.5	3	—
10	Cu/HMPC	5/0.081	80	4.5	36	278/62
11	Cu/HMPC	15/0.243	80	4.5	79	823/183
12	Cu/HMPC	10/0.162	100	4.5	68	1049/233
13	Cu/HMPC	10/0.162	100	11	89	1373/125
14	Cu/HMPC	10/0.162	85	4.5	89	1373/305
15	Cu/HMPC	10/0.162	85	15	90	1389/93
16	Cu/HMPC	10/0.162	70	4.5	85	1312/291
17	Cu/HMPC	10/0.162	60	4.5	34	525/117
18	Cu/HMPC	10/0.162	60	15	67	1034/69
19	Cu/HMPC	10/0.162	80	2	53	818/409
20	Cu/HMPC	10/0.162	80	6	99	1528/255
21^d^	Cu/HMPC	10/0.162	80	12	15	92/8
22^e^	Cu/HMPC	10/0.162	80	4.5	55	849/189
23^f^	Cu/HMPC	10/0.162	80	4.5	29	448/100
24^g^	Cu/HMPC	10/0.162	80	4.5	78	1203/268
25	HMPC-1	10/—	80	4.5	41	—
26	Cu/HMPC-1	14.7/0.162	80	4.5	66	1019/226
27^h^	HMPC-1	10/—	100	14	83	—
28^h^	Cu/HMPC-1	14.7/0.162	100	11	95	1466/140

a Reaction condition: benzylamine (5 mmol), solvent-free, air atmosphere.

b GC yield.

c TON/TOF [TON = the amount of product (mol)/the amount of active sites; TOF = TON/time (h)].

d 1 mmol of benzylamine was used.

e N_2_ atmosphere.

f In H_2_O (5 mL).

g Cu/HMPC was prepared by mix and heat method.

h Reaction was carried out under O_2_ atmosphere.

### 3.3. Scope of Cu/HMPC system

Under the optimized reaction conditions, a series of amines such as aliphatic, cyclic secondary, heteroaromatic, benzylic and their derivatives was transformed into corresponding imines in excellent to moderate yields (98–61%) with high selectivity and TON/TOF values (1512/339–833/35 h^−1^) (Table 2, entries 2a–2o). The self-coupling of benzylamine (1a) gave the desired product (2a) in excellent yield of 98% (100% selectivity) with very high TON/TOF values of 1512/336 h^−1^ (Table 2, entry 2a). It was found that the present Cu/HMPC system efficiently transformed substituted benzylamines [benzylamine containing either electron–donating groups (–CH_3_ and –OCH_3_)] or electron-withdrawing (F and Cl) groups] into corresponding imines (Table 2, entries 2b–2g). A better yield of 89% and 92% was obtained from the self-coupling of 3-methyl benzylamine (1b) and 4-methyl benzylamine (1c) respectively (Table 2, entries 2b and 2c), whereas 2-methyl benzylamine (1d) gave 78% of 2d even after 8.5 h (Table 2, entry 2d). The steric effect might have played a crucial role in affecting the reaction rate. Alike, the Cu/HMPC system afforded 92% of 2e with an excellent selectivity (100%) from the self-coupling of 4-methoxy benzylamine (1e) (Table 2, entry 2e). Electron-withdrawing groups (F and Cl) substituted benzylamines, 4-flurobenzylamine (1f) and 4-chlorobenzylamine (1g), homocoupled to offer corresponding imines (2f and 2g) in excellent yields (92% and 78%) with good selectivity (99% and 100%) and TON/TOF values (1404/134 h^−1^ and 1204/89 h^−1^) (Table 2, entries 2f and 2g). Interestingly, the present catalytic system converted the less reactive 2-phenylethanamine (1h) into corresponding imines (2h) in 61% (Table 2, entry 2h).

**Table 2:** Aerobic oxidation of amines to imines^a^

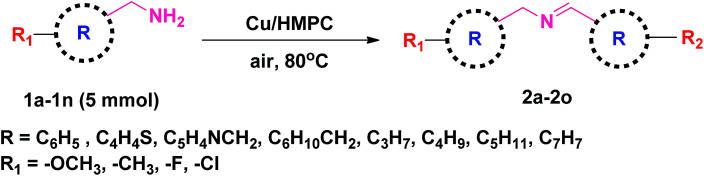
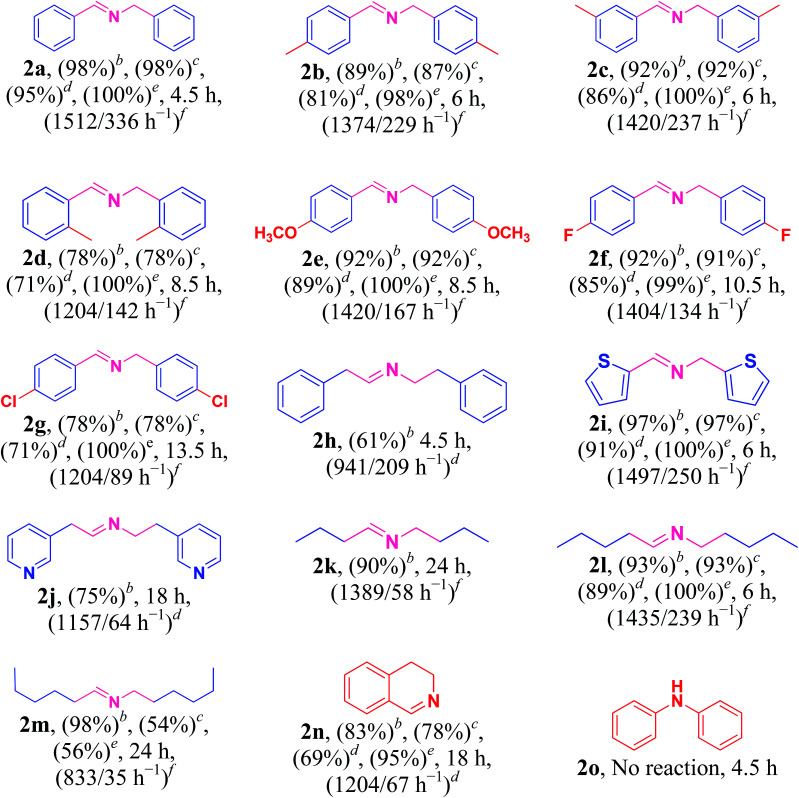

a Reaction conditions: amine (5 mmol), Cu/HMPC (10 mg, Cu 0.162 mol%), air atmosphere, 80 °C.

b GC conversion.

c GC yield.

d Isolated yield.

e Selectivity.

f TON/TOF.

Heterocyclic amines containing N and S atom (which are usually poison most of the metal catalysts) could also be efficiently converted into corresponding imines (Table 2, entries 2i and 2j). For example, thiophen-2-ylmethanamine (1i) catalyzed by Cu/HMPC to obtain its corresponding imine (2i) in excellent yield of 97% with 100% selectivity and high 1497/250 h^−1^ values (Table 2, entry 2i). A moderate conversion of 75% (2j) was achieved from the self-coupling of pyridin-3-ylmethanamine (1j) (Table 2, entry 2j).

The present Cu/HMPC system was further extended with aliphatic amines. Long-chain aliphatic amines are often considered as inactive due to the presence of α-hydrogen, and most of the previously reported catalytic systems are largely failed in the transformation of long chain aliphatic amines. Interestingly, the Cu/HMPC is highly active towards the long chain aliphatic amines (Table 2, entries 2k–2m). For instance, pentylamine (1l) was self-coupled to obtain the corresponding imine, (*E*)-*N*-pentylidenepentan-1-amine (2l), in good 93% yield with 100% selectivity (Table 2, entry 2l). The TON/TOF values were calculated to be 1435/239 h^−1^. Alike, 90% of the butylamine (1k) was converted by the Cu/HMPC under the optimized reaction conditions (Table 2, entry 2k). A moderate yield of 2m (54%) was offered from the coupling of hexylamine (1m) (Table 2, entry 2m). In addition, the Cu/HMPC is capable of oxidizing 1,2,3,4-tetrahydroisoquinoline (1n) to 3,4-dihydroisoquinoline (2n) in a good yield of 77% with 95% selectivity (Table 2, entry 2n). Attempts were made to oxidize piperidine and aniline (1o) also but the reactions were not successful.

Scale reaction was performed to recognize the effectiveness of the Cu/HMPC system. Generally, the activated carbon based catalysts are often limited due to poor yields in scale reactions. In order to achieve better yields, the catalytic systems require harsh reaction conditions such as high temperature (over 100 °C). For instance, mesoporous carbon derived from vitamin B12 (dm-VB_12_-6) achieved 95% of 2a from the self-coupling of 1a under O_2_ atmosphere at 120 °C for 48 h.^29^ In the present case, 91% of the desired product (2a) was achieved when the mixture of Cu/HMPC (100 mg) and 1a (100 mmol) was stirred at 80 °C for 68 h. The TON/TOF values are calculated to be very high as 2809/41 h^−1^.

To further extend the range of usefulness, we examined the Cu/HMPC catalyst for the cross coupling of amines with aniline (1o) under optimized reaction conditions (Table 3, 3a–3d). Initially, the reaction conditions such as substrate concentration and temperature were optimized. We found that the best ratio of benzylamine (1a) to aniline (1o) is 1 : 4. At high temperatures (90 °C), the catalytic system showed poor selectivity, whereas, at low temperature (70 °C), yield of the product was low. Most of the catalytic systems suffer from poor selectivity of the cross-coupled products. To our delight, in the presence of Cu/HMPC, benzylamine was selectively coupled with aniline to offer (*E*)-*N*-benzylideneaniline (3a) in an excellent yield of 98%, whereas only 2% of homocoupled product (2a) was determined. Similarly, 4-methylbenzylamine (1b) and 3-methylbenzylamine (1c) were selectively coupled with aniline (1o) to obtain (*E*)-*N*-(4-methylbenzylidene)aniline (3b) and (*E*)-*N*-(3-methylbenzylidene)aniline (3c) respectively. Heterocyclic amine, thiophen-2-ylmethanamine (1i) also coupled efficiently with aniline to offer cross-coupled product (*E*)-*N*-(thiophen-2-ylmethylene)aniline (3d) in an excellent 98% yield and selectivity. The results confirmed that the Cu/HMPC is efficient for cross-coupling of amines as well.

**Table 3:** Aerobic oxidative cross coupling of amines with aniline^a^

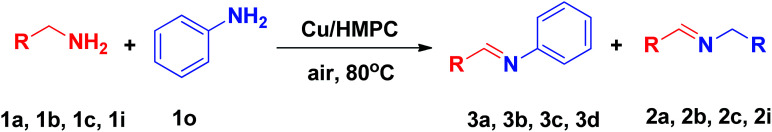
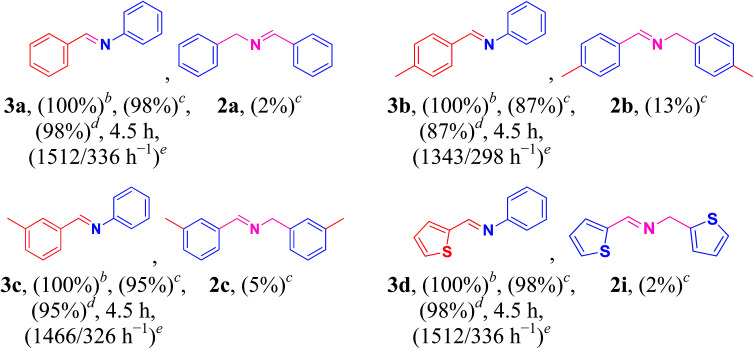

a Reaction conditions: amines (1 mmol) and aniline (4 mmol), Cu/HMPC (10 mg), 80 °C, air atmosphere.

b GC conversion based on amines (1a, 1d, 1e, and 1h).

c GC yield.

d GC selectivity.

e TON/TOF.

### 3.4. Comparison of Cu/HMPC activity over previously reported catalysts

The catalytic performance of the present Cu/HMPC is better to many other heterogeneous catalysts (see Table 4) including AuNPs/SBA-NH_2_,^4^ MOF-253,^5^ Cs/MnO_*x*_,^6^ Cu(0),^7^ CuCl,^8^ graphite oxide,^19^ Pc-Ludox-8,^13^ Degussa P25-TiO_2_,^9^ mpg-C_3_N_4_,^43^ m-VB_12_-8,^29^ α-MnO_2_,^10^ NHPI/Fe(BTC),^11^ CuO–CeO_2_,^12^ Ce–Sm/SiO_2_,^45^ AuONT-THAP,^14^ CeO_2_–MoO_3_/SiO_2_,^15^ Ir PCP pincer complex^48^ and V.^16^ Among them, most of the catalysts are expensive and difficult to prepare when compare to eco-catalysts. As noted in Table 1, the activity of Cu/HMPC is better than the catalysts (Cu-HMPC-1 and HMPC-1) prepared from Cu-polluted plants obtained in mining operation area or agricultural area. The complete characterizations of HMPC-1 and Cu/HMPC-1 (TEM, Raman, XRD and SEM-EDS) are provided in the ESI (Fig. S10–S15 in ESI†). Unlike Cu/HMPC, the morphology of HMPC-1 and Cu/HMPC-1 is different with the absence of interconnected 1D/2D/3D form. The elemental composition of Cu/HMPC is one of the key factors for the interconnected 1D/2D/3D morphology of Cu/HMPC. Therefore, we concluded that the well-balanced elemental composition in Cu/HMPC could also be a reason for the better activity. In fact, it is nearly impossible to avoid the plant uptake of unwanted elements that present in the mining area soil. Moreover, the main drawback of utilizing Cu-polluted plants obtained in mining operation area is the poor reproducibility. Since the present method is highly effective, reliable and reproducible, we believe that the present method could be highly helpful to the mining industries. Based on the experimental results obtained, we found that the superior activity of the Cu/HMPC under mild reaction condition is maybe due to seven reasons; (a) higher surface area of the Cu/HMPC, (b) synergetic effect between Cu and other elements presented in the Cu/HMPC, (c) higher order of CuO species dispersed in Cu/HMPC, (d) chemical bonding between the Cu-species and carbon matrix (HMPC), (e) very fine dispersion of catalyst even under solvent free conditions, (f) presence of defect sites, and (g) pores presented in the Cu/HMPC. Particularly, the Cu-species in Cu/HMPC is the main factor for the efficient improvement in the catalytic activity among the above mentioned reasons. In fact, although the BET surface area of HMPC (without Cu-species, 1295.0 m^2^ g^−1^) is found to be twofold higher than the Cu/HMPC (620.8 m^2^ g^−1^), the HMPC showed just 57% of the catalytic product (Table 1, entry 6) whereas Cu/HMPC gave 98% of 2a (Table 1, entry 8). Moreover, we believe that the existence of the Cu-species in the HMPC matrix is one of the key factors in forming the interconnected 1D/2D/3D form of the HMPC carbon matrix. In fact, HMPC without Cu-species showed no 1D/2D/3D form in its carbon matrix. In order to understand the effect of structure on the catalytic activity, the Cu-species were removed from Cu/HMPC and HMPC with interconnected 1D/2D/3D morphology (HMPC-2) was obtained (see Section ‘3.7. sustainability of Cu/HMPC’ for more details). Under the optimized reaction conditions, the HMPC-2 gave 67% of 2a with 100% selectivity which is slightly higher when compared to HMPC-1 (HMPC with no interconnected 1D/2D/3D morphology). In addition, other materials such as graphite oxide (GO),^19^ m-VB_12_-8 (ref. 25) and hollow carbon spheres (HCSs)^49^ with similar 1D or 2D or 3D structures were also tested under the present reaction conditions. A poor yield of 33, 37 and 41% with 100% selectivity was afford by GO, m-VB_12_-8 and HCSs respectively. The results showed that the superior catalytic activity of the Cu/HMPC is mainly due to the presence of Cu-species.

**Table 4:** Comparison of present Cu/HMPC catalyst over other heterogeneous catalysts

S. no	Catalyst	Amount of catalyst (mg mol^−1^%)	Solvent (mL)	Temp. (°C)	Atmosphere	Time (h)	Yield (%)	TON/TOF h^−1^
1 (ref. 4)	AuNPs/SBA-NH_2_	30/0.76	Toluene (3)	100	O_2_ (1 atm)	24	90	4.91/0.25
2 (ref. 5)	MOF-253	—/1.5	b	100	O_2_ (1 bar)	6	>99	66/11
**3** a	**Cu/HMPC**	**10/0.162**	b	**80**	**air**	**4.5**	**98**	1512/36
4 (ref. 6)	Cs/MnO_*x*_	25/—	Toluene (5)	110	Air balloon	3	82	3.33/1.11
5 (ref. 7)	Copper(0)	—/0.05	b	90	Air	20	88	1760/88
6 (ref. 8)	CuCl	—/0.05	b	100	Air	18	88	1760/98
7 (ref. 19)	Graphite oxide	50/—	b	100	O_2_ (5 atm)	4	98	—
8 (ref. 13)	Pc-Ludox-8	20/—	b	100	O_2_ balloon	5.5	94.2	—
9 (ref. 9)	TiO_2_-degussa P25	10/—	Water (2)	Hg lamp	Air	9	63	—
10 (ref. 47)	mpg-C_3_N_4_	50/—	CH_3_CN (10)	80	O_2_ (0.5 MPa)	3.5	99	—
11 (ref. 20)^c^	m-VB_12_-8	10/—	Heptane (1)	100	O_2_ balloon	12	97	—
12 (ref. 10)^d^	α-MnO_2_	—/10	MeCN (2)	RT	Air	4	95	—
13 (ref. 11)	NHPI/Fe(BTC)	75/—	b	100	O_2_ balloon	24	89	—
14 (ref. 12)	CuO–CeO_2_	—/5	DMSO (3)	110	Air	22	90	—
15 (ref. 46)	Ce–Sm/SiO_2_	200/—	b	120	O_2_ (20 mL min^−1^)	4	69	—
16 (ref. 14)^e^	AuONT-THAP	—/0.1–3	H_2_O (1)	27 °C	Air	24	95	—
17 (ref. 15)	CeO_2_–MoO_3_/SiO_2_	100/—	b	120	O_2_ (20 mL min^−1^)	4	98	—
18 (ref. 16)	V	21/5	CH_3_CN (2)	60	Air (0.1 MPa)	12–24	96	—

a Present work.

b Solvent free condition.

c Base was used.

d *tert*-Butyl hydroperoxide (TBHP) used.

e co-catalyst was used.

### 3.5. Proposed mechanism

**Fig. 9:**
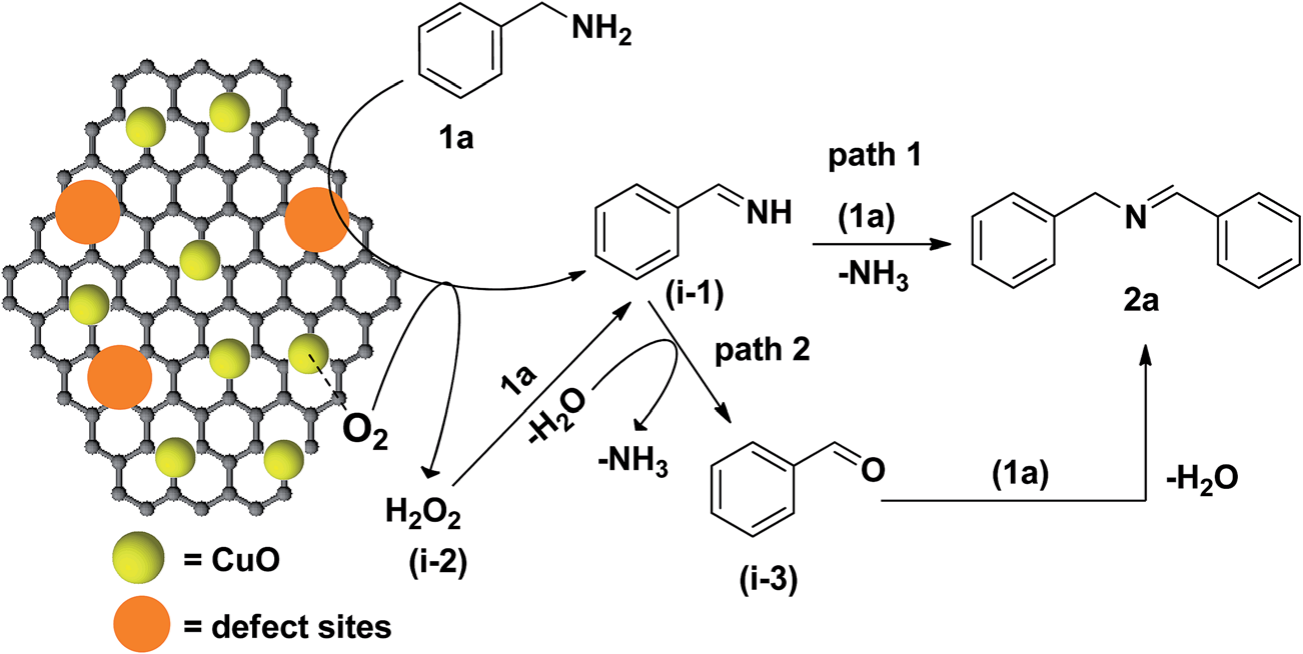
Proposed mechanism for Cu/HMPC catalyzed self-coupling of benzylamine (1a) to 2a.

Based on the optimization results and earlier reports,^25,50–52^ two different routes (path 1 and path 2) are proposed for the Cu/HMPC catalyzed self-coupling of amines (Fig. 9). At first, both benzylamine (1a) and oxygen molecules (accessed from air atmosphere) were activated respectively by defect sites and CuO species of Cu/HMPC to form benzylimine (i-1) and H_2_O_2_ (i-2) intermediates. Unlike the monometallic catalysts, in the present system, the polymetallic nature of the Cu/HMPC [synergistic effect between metals (including Cu) present in the Cu/HMPC] would have also assisted for the better and faster activation of both 1a and oxygen molecules.^51^ In path 1, the benzylimine (i-1) intermediate reacts with another nucleophilic benzylamine molecule to form the targeted dibenzylamine product (1b). In path 2, the H_2_O_2_ intermediate undergoes oxidation reaction with 1a to form i-1 which further hydrolyzes to form benzaldehyde (i-3). Finally, i-3 reacts with another 1a to offer 2a. The formation of benzaldehyde (i-3) was confirmed by the GC analysis. Although, two paths are possible, under solvent-free conditions, it is likely that a reasonable amount of water occluded in the Cu/HMPC could play a key role in facilitating the targeted product (see Fig. 8). Therefore we suspect that the present Cu/HMPC catalytic system mainly follows path 2. Similarly, in case of cross-coupling reaction, the intermediates formed from benzylamine [benzylimine (i-1) and benzaldehyde (i-3)] further react with aniline to obtain (*E*)-*N*-benzylideneaniline (3a).

### 3.6. Heterogeneity, stability, versatility and reusability of Cu/HMPC

**Fig. 10:**
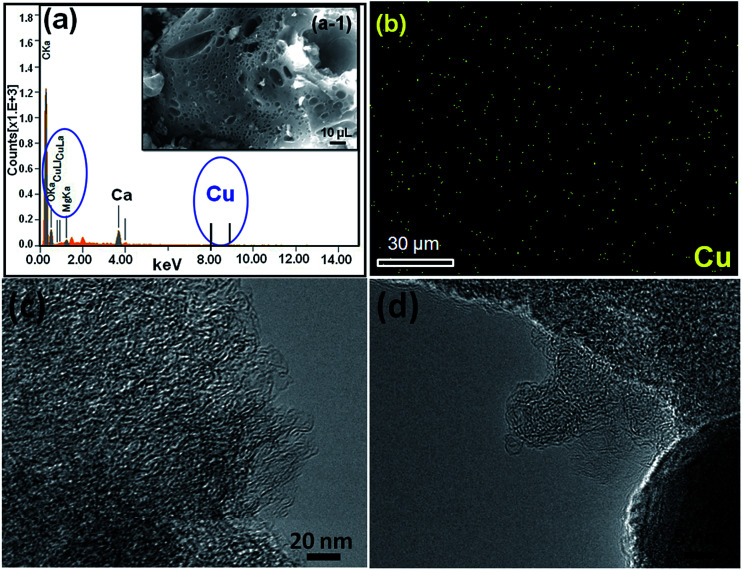
(a) EDS spectrum, (a-1) SEM image, (b) Cu elemental mapping, (c and d) TEM images of used Cu/HMPC.

Heterogeneity and stability of the Cu/HMPC were confirmed by investigating the Cu/HMPC after use in coupling reactions. Fig. 10 shows the HR-TEM images, SEM image, EDS spectrum and corresponding elemental mappings (Cu, O, Mg, C, Al, Ca, P, Al) of used Cu/HMPC. Unlike other supported catalysts, the present Cu/HMPC eco-catalyst is very stable. Compare to fresh Cu/HMPC, no obvious changes were observed in the morphology of used Cu/HMPC (Fig. 10). The reaction conditions did not cause any significant change in morphology of Cu/HMPC. The interconnected 1D/2D/3D structure (needle/sheets/hallow-spheres) of Cu/HMPC was well-maintained. The content of Cu in used Cu/HMPC (0.99%) was determined to be almost similar to that of fresh Cu/HMPC (1.03). The result proves that there is no leaching of Cu from Cu/HMPC during the course of reaction and true heterogeneous nature of the Cu/HMPC. The elemental mapping of Cu, O, Mg, C, Ca, P and Al demonstrates a fine dispersion of elemental species similar to the fresh one (Fig. 10, 3 and 4).

Inspired by the results obtained, Cu/HMPC was further applied in aza-Michael reaction to offer β-aminocarbonyl compounds. The β-aminocarbonyl compounds are biologically important products and are commonly used as key intermediates for the synthesis of N-containing compounds.^18^ We performed aza-Michael reaction under solvent-free reaction conditions at 25 °C. Initially, reaction time, catalyst amount and temperature were optimized. As shown in Table 5, wide range of amines and acrylamide proceeded smoothly to obtain aza-adduct in excellent to good yields with very high TON/TOF values (Table 5, entries 4a–4g). A less amount of catalyst is also sufficient for the reaction. Piperidine was readily coupled with methyl but-3-enoate and but-3-enenitrile to give methyl 3-(piperidin-1-yl)propanoate (4a) and 3-(piperidin-1-yl)propanenitrile (4b) respectively, in excellent yields with 100% selectivity (Table 5, 4a and 4b). Similarly, 89% of methyl 3-morpholinopropanoate (4c) was isolated from the coupling of morpholine with methyl but-3-enoate (Table 5, entry 4c). The TON and TOF values were calculate to be 2069 and 12 389 h^−1^ respectively. Interestingly, bis-aza adducts were obtained by using benzylamine as Michael donors. The catalytic system offered dimethyl 3,3′-(benzylazanediyl)dipropanoate (4d) and 3,3′-(benzylazanediyl)dipropanenitrile (4e) in moderated yields of 77% and 71% respectively, but 100% selectivity was maintained (Table 5, entries 4d and 4e). The moderate yields may be obtained due to the presence of sterically hindered phenyl group. These results suggest that Cu/HMPC is highly efficient for the aza-Michael reaction.

**Table 5:** Cu/HMPC catalyzed aza-Micheal reaction^a^^,^^b^^,^^c^^,^^d^

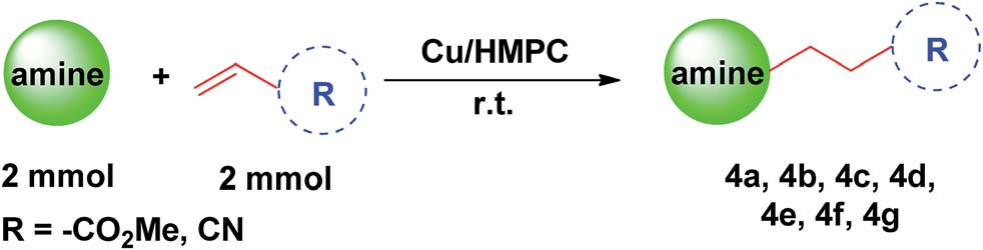
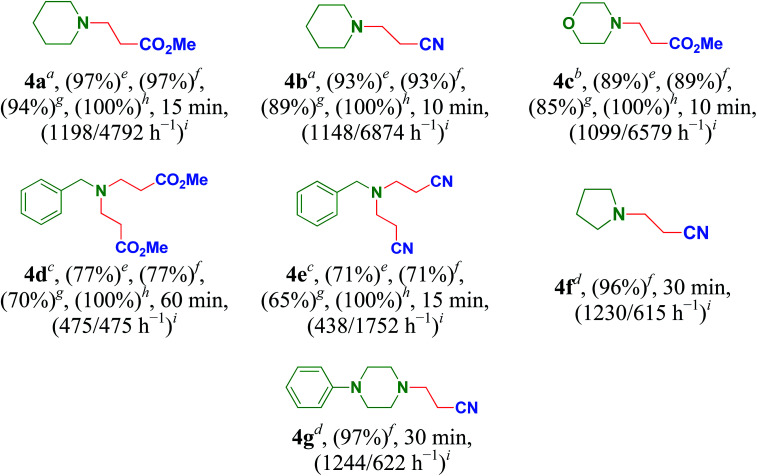

a Reaction condition: amines (2 mmol), α,β-unsaturated compounds (2 mmol), Cu/HMPC (10 mg), 25 °C.

b Reaction condition: amines (2 mmol), α,β-unsaturated compounds (2 mmol), Cu/HMPC (10 mg), 25 °C.

c Reaction condition: amines (1 mmol), α,β-unsaturated compounds (2 mmol), Cu/HMPC (10 mg), 25 °C.

d Reaction condition: amines (2 mmol), α,β-unsaturated compounds (2 mmol), used Cu/HMPC (10 mg), 25 °C.

e GC conversion.

f GC yield.

g Isolated yield.

h Selectivity.

i TON/TOF.

Reusability is one of the important advantages of the heterogeneous catalyst. Most of the eco-catalytic systems fail to demonstrate the recyclability. The recyclability of Cu/HMPC was tested in both coupling and aza-Michael reactions. After first cycle, the Cu/HMPC was washed well with diethyl ether and dried at 60 °C for several hours. A good yield of 2a with 100% selectivity was obtained at second cycle. The yield moderately decreased after second cycle. However, heating the used catalyst (after second cycle) at 400 °C for 1 h improved the yield of 2a. The lower yield afford by used Cu/HMPC may be due the blocking of active sites by products and reactants. It was noticed that the Cu/HMPC gave 92% of 4a with 100% selectivity even at 4^th^ cycle. Overall, the results demonstrate that the Cu/HMPC eco-catalyst is stable, heterogeneous, reusable and versatile.

### 3.7. Sustainability of Cu/HMPC

**Fig. 11:**
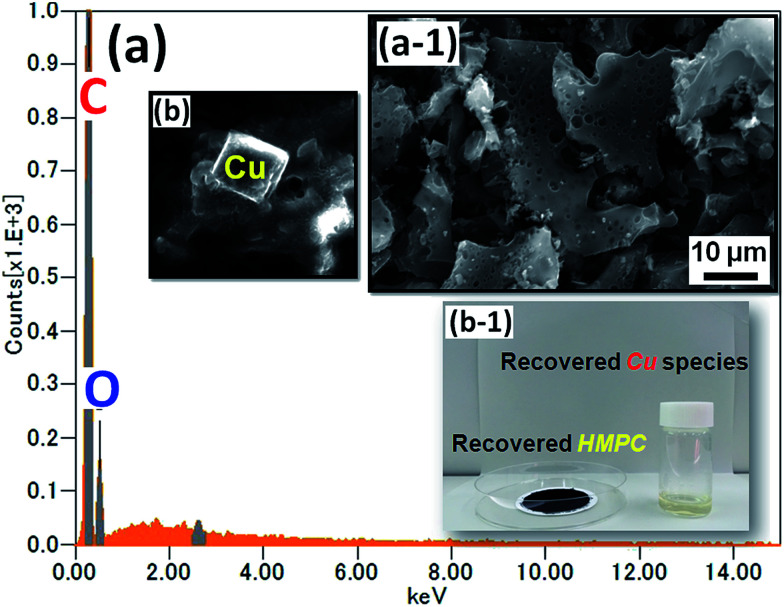
(a) EDS spectrum and (a-1) SEM image of recovered HMPC. (b) Recovered Cu, and (b-1) optical image of recovered HMPC and Cu.

According to Grison *et al.*,^53,54^ metal species recovered from eco-friendly plant-based material can be an efficient catalysts for various organic transformations. Very recently, Escande and co-workers^55^ prepared eco-Mn heterogenous catalyst from leaves of Mn-hyperaccumulating plant *Grevillea exul* subsp. *rubiginosa* (Proteaceae) and used for alkene epoxidation/oxidative cleavage. They found that the eco-Mn catalyst is highly efficient and versatile. In the present work, we have extracted the Cu-species from the used Cu/HMPC by using HCl to demonstrate the sustainability of the catalyst. Fig. 11 shows SEM image and EDS spectrum of recovered HMPC and Cu. The optical images of recovered HMPC and Cu (including other species such as Al and Mg) are also shown in Fig. 11. The SEM images and corresponding EDS spectrum of HMPC confirm the prescience of C (81 wt%) and O (19 wt%) elements only (Fig. 11a and a-1). Moreover, the interesting 1D/2D/3D morphology with porous nature was certainly maintained. Alike, successful recovery of Cu including other elements such as Mg, Ca, Al, K, and O was confirmed by the SEM-EDS results (Fig. 11, S6 and S7†). Recovery of Cu and HMPC from Cu/HMPC is one of the hallmarks of this present work. We presume that the recovered Cu species and HMPC would be useful for further applications.

## 4. Conclusion

Highly efficient and mild Cu-based eco-catalytic system was developed for imines synthesis and aza-Michael reactions. The Cu/HMPC successfully coupled a wide range of amines to imines under environmentally feasible conditions such as open air atmosphere, low temperature (80 °C) and low amount of catalyst (10 mg, 0.162 mol% Cu). The catalyst Cu/HMPC gave coupled products in excellent yields (98–61%) with very high TON/TOF values (1512/339–833/35 h^−1^). To the best of our knowledge, this is one of the most efficient Cu-based heterogeneous eco-catalysts reported to date for the (cross)coupling of amines. The versatility and reusability of the Cu/HMPC was also confirmed by excellent yields of used Cu/HMPC in aza-Michael reactions. The Cu species was successfully recovered from Cu/HMPC by a simple HCl treatment. Heterogeneity and reusability of Cu/HMPC were also found to be good. Overall, due to reliability and reproducibility, we believe that the present method can be highly helpful to the mining industries.

## Conflicts of interest

The authors declare no competing financial interest.

## Supplementary Material

RA-008-C7RA12470H-s001
