# Supplementary material for: Cultivation of a Cu/HMPC catalyst from a hyperaccumulating mustard plant for highly efficient and selective coupling reactions under mild conditions
Source: RSC Adv. 2018 Jan 24;8(9):4531–47. doi: 10.1039/c7ra12470h (PMC9077870; doi:10.1039/c7ra12470h)
Supplement: RA-008-C7RA12470H-s001 [file RA-008-C7RA12470H-s001.pdf]

Electronic Supplementary Material (ESI) for RSC Advances.
This journal is © The Royal Society of Chemistry 2017

Electronic supplementary information (ESI)

Cultivation of Cu/HMPC catalyst from hyperaccumulating mustard plant for highly efficient and selective coupling reactions under mild conditions

Mayakrishnan Gopiraman,[b,1] Kai Wei,[c] Ke-Qin Zhang,[c] Ill-Min Chung[b,*] Ick Soo Kim[a,*]

[a]*Nano Fusion Technology Research Group, Division of Frontier Fibers, Institute for Fiber Engineering (IFES), Interdisciplinary Cluster for Cutting Edge Research (ICCER), Shinshu University, Tokida 3-15-1, Ueda, Nagano Prefecture, 386-8567, Japan. E-mail: kim@shinshu-u.ac.jp*

[b]*Department of Applied Bioscience, College of Life & Environment Science, Konkuk University, 120 Neungdong-ro, Gwangjin-gu, Seoul 05029, South Korea. E-mail: imchung@konkuk.ac.kr*

[c]*National Engineering Laboratory for Modern Silk (NESLab), College of Textile and Clothing Engineering, Soochow University, Suzhou, China*

Experimental section

Preparation of Hogland nutrient solution. The modified Hoagland's solution was prepared by mixing three different nutrient solutions such as microelement nutrient solution, ferric salt solution and macroelement nutrient solution. At first, stock solution of these three different nutrient solutions were prepared. Then the prepared microelement nutrient solution and ferric salt solution were mixed into macroelement nutrient solution at particular concentration.[1,2]

The macroelement nutrient solution was prepared in aqueous solution containing calcium nitrate tetrahydrate (945 mg/L), potassium nitrate (506 mg/L), ammonium nitrate (80 mg/L), potassium phosphate monobasic (136 mg/L), magnesium sulphate (493 mg/L), ferric salt solution (2.5 ml), microelement nutrient solution (5 ml, pH=6.0).

The ferric salt solution was prepared by dissolving ferrous sulfate (2.78 g) and ethylenediaminetetraacetic acid disodium salt (EDTA-2Na, 3.73 g) in 0.5 L water.

Similarly, potassium iodide (0.83 mg/L), orthoboric acid (6.2 mg/L), manganese sulphate (22.3 mg/L), zinc sulfate (8.6 mg/L), sodiummolybdatedehydrate (0.25 mg/L), copper sulfate (0.025 mg/L), and cobalt chloride (0.025 mg/L) were dissolved in 1L of water to prepare microelement nutrient solution.

Reference

(1) T. Barac, S. Taghavi, B. Borremans, A. Provoost, L. Oeyen, J. V. Colpaert, J. Vangronsveld, D. van der Lelie, *Nat. Biotechnol.*, 2004, **22**, 583–588.

(2) D. Ye, T. Li, Z. Zheng, X. Zhang, G. Chen, H. Yu, *Front. Plant. Sci*. 2015, **6**, 1–10.

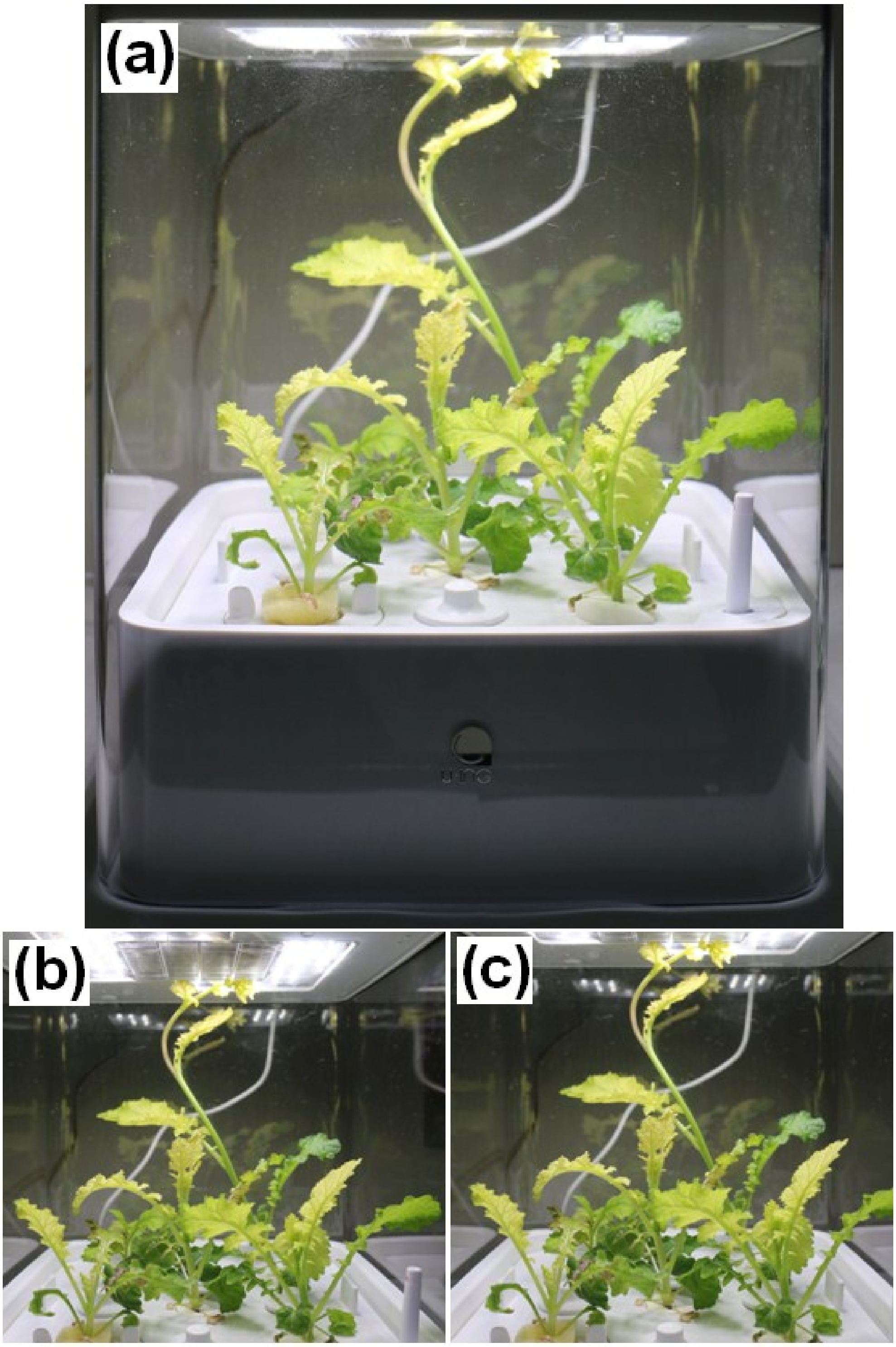

Fig. S1. Cultivation of HMP using Cu^{2+} nutrient solution.

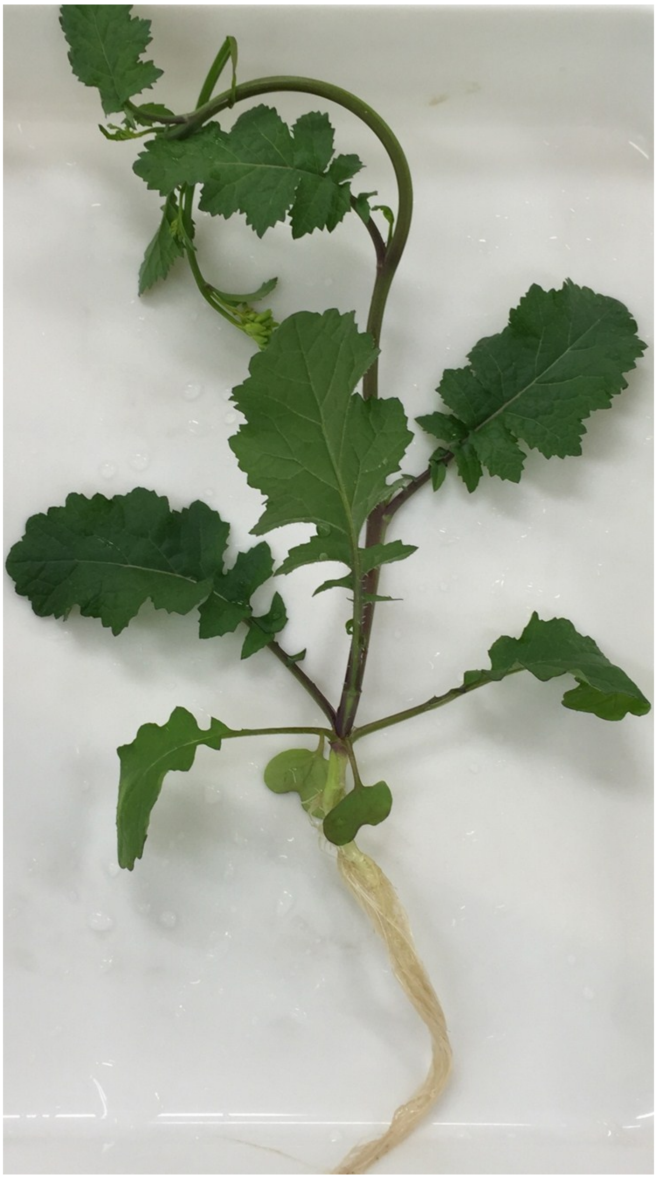

Fig. S2. Optical image of well-grown Cu/HMP plant.

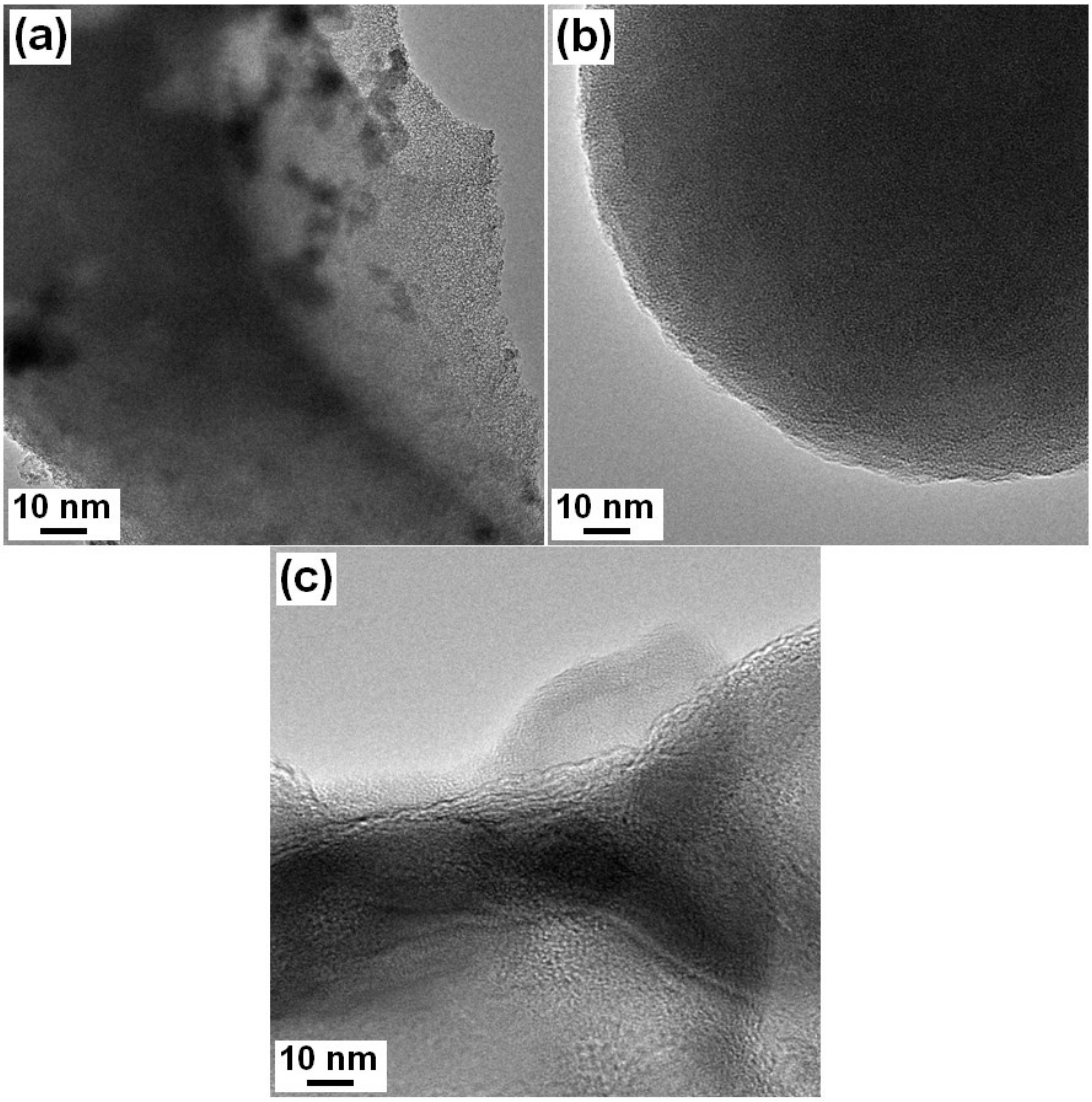

Fig. S3. HR-TEM images of Cu/HMPC.

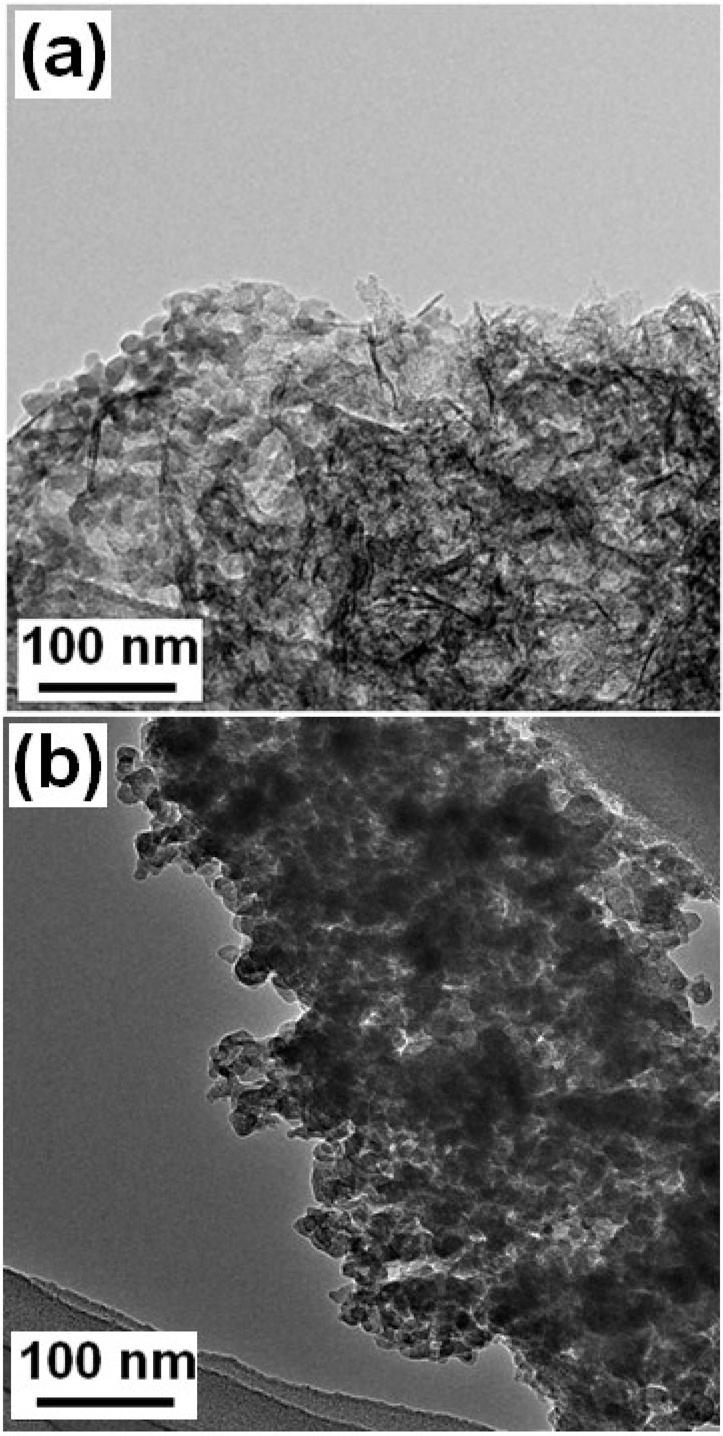

Fig. S4. HR-TEM images of Cu/HMPC show the interconnected 1D, 2D and 3D morphology.

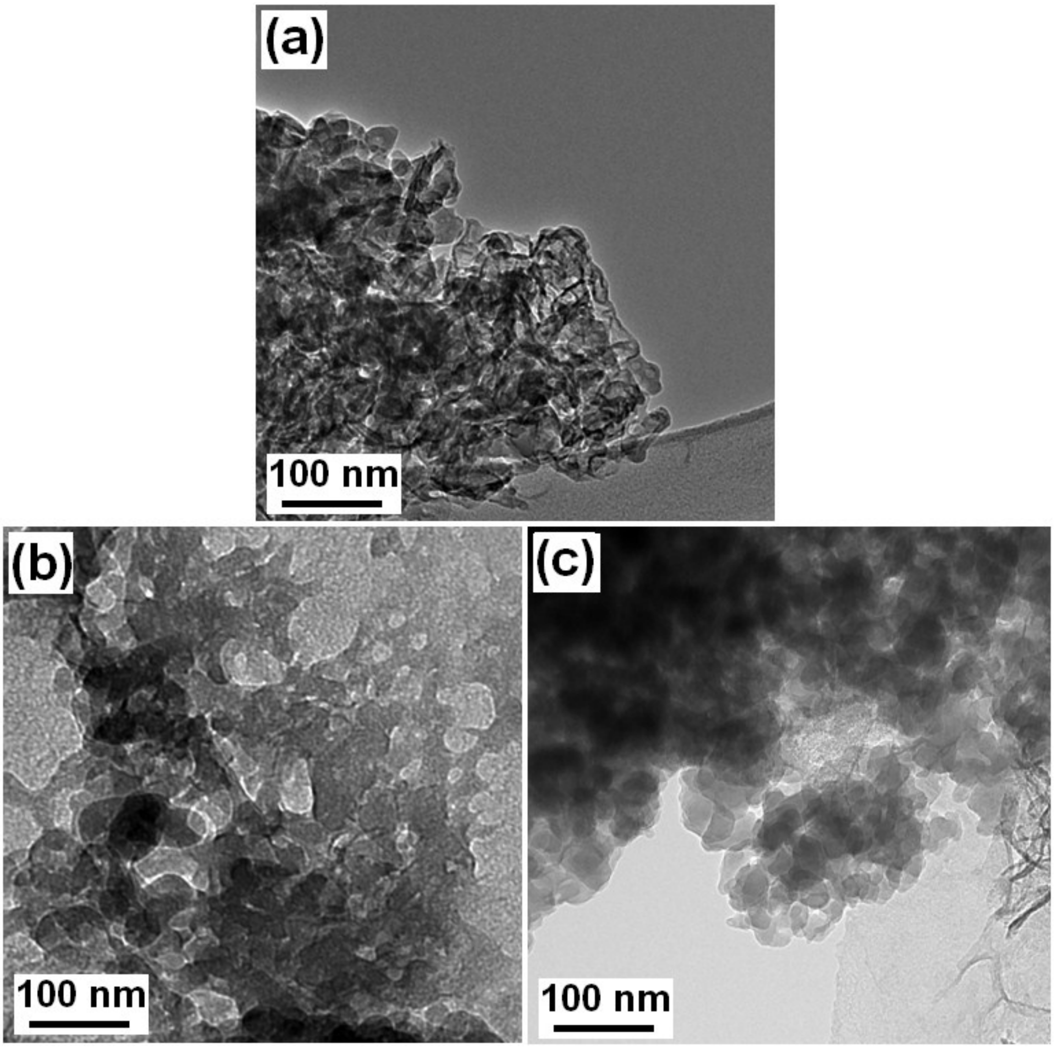

Fig. S5. TEM images of Cu/HMPC.

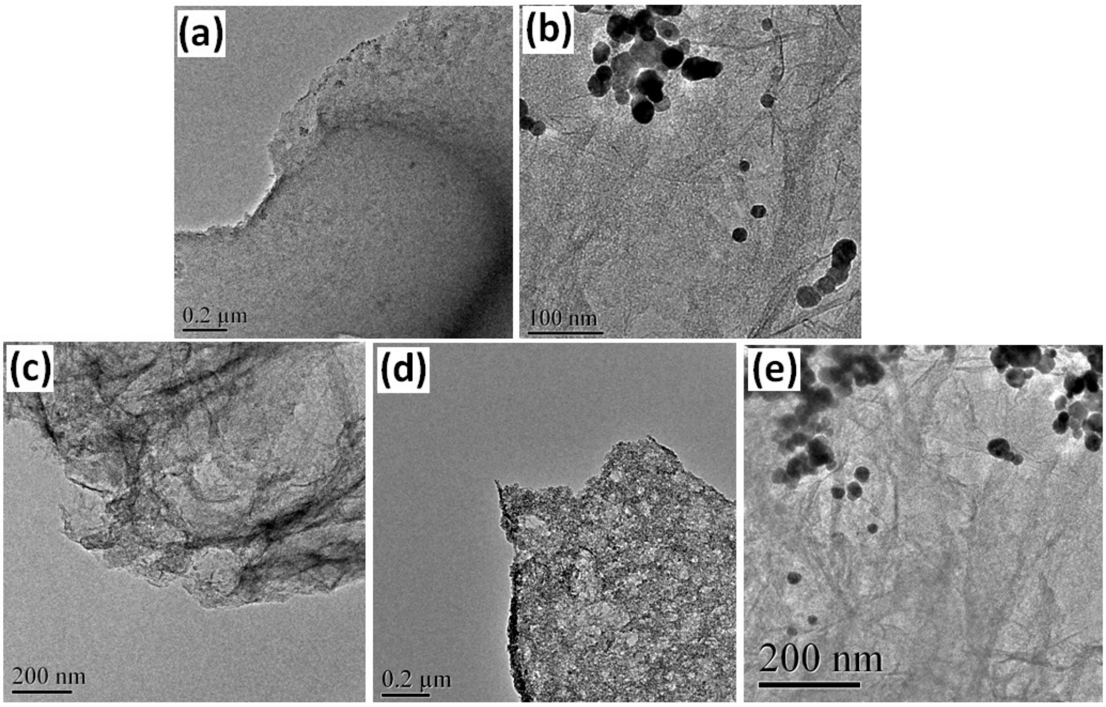

Fig. S6. TEM images of HMPC.

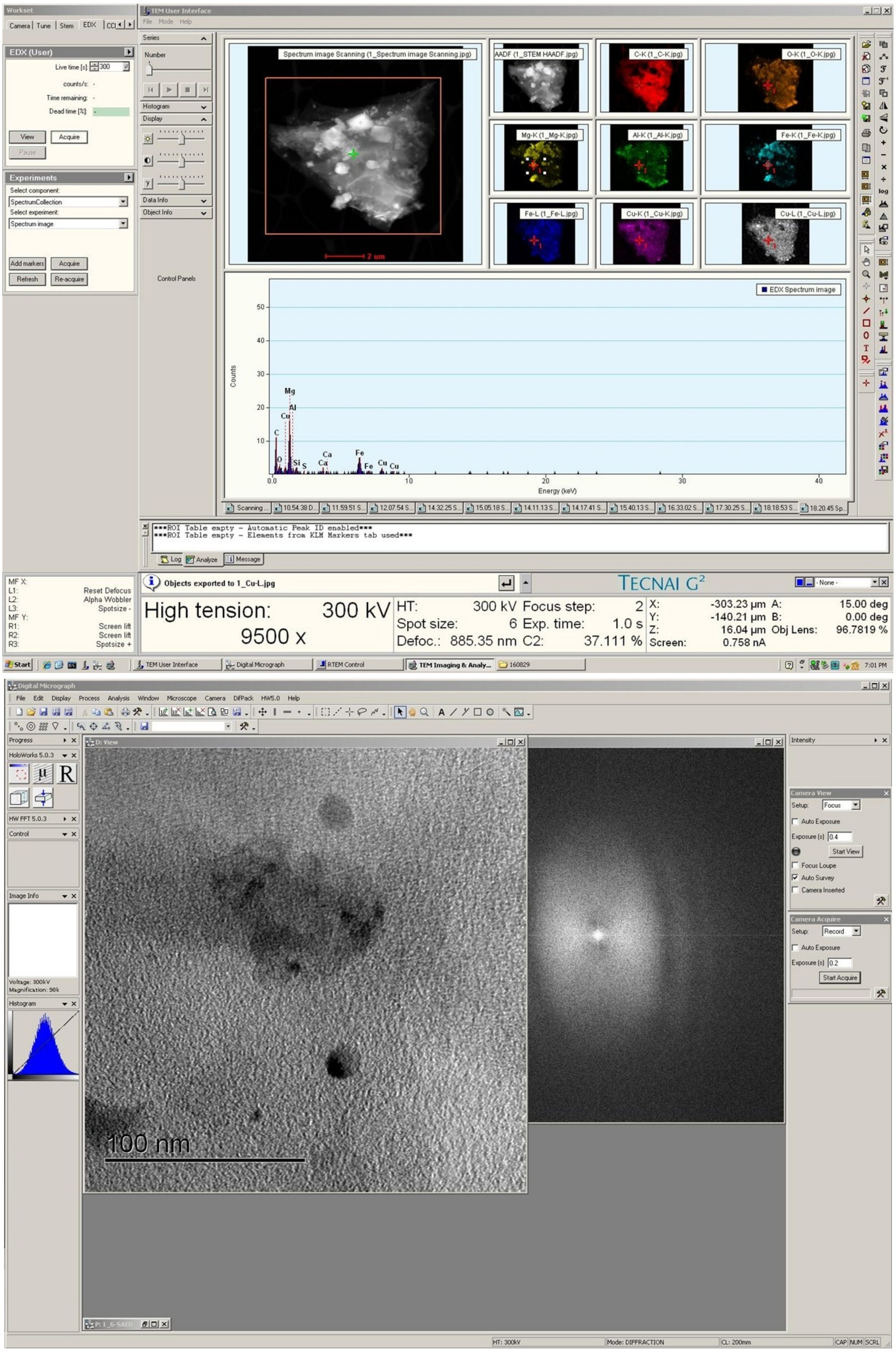

Fig. S7. The screenshot was taken while analysis HR-TEM, SAED and elemental mapping of Cu/HMPC.

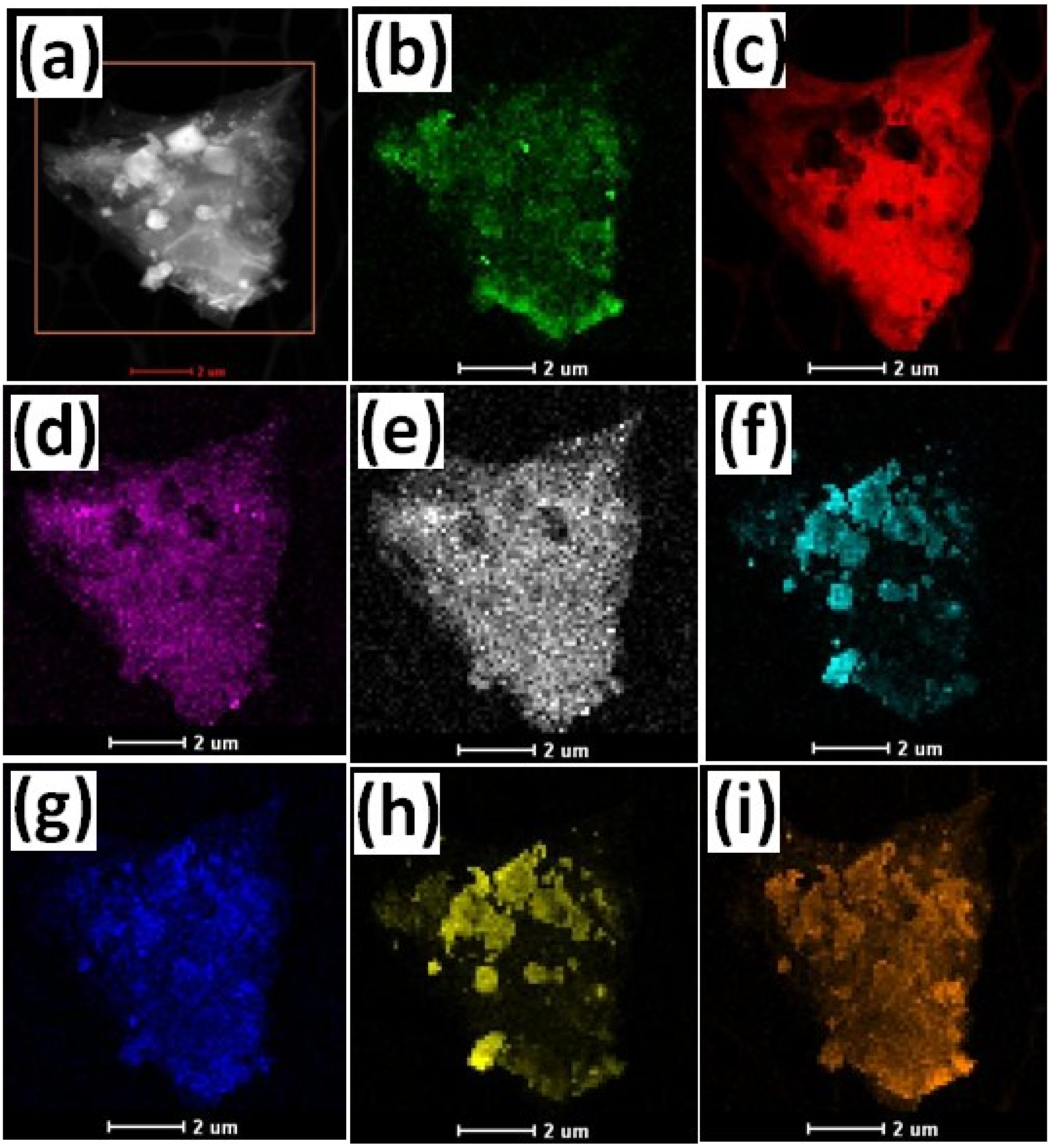

Fig. S8. (a) HRTEM image of Cu/HMPC and corresponding mapping of (b) Al, (c) C, (d) Cu-K, (e) Cu-L, (f) Fe-K, (g) Fe-L, (h) Mg, and (i) O.

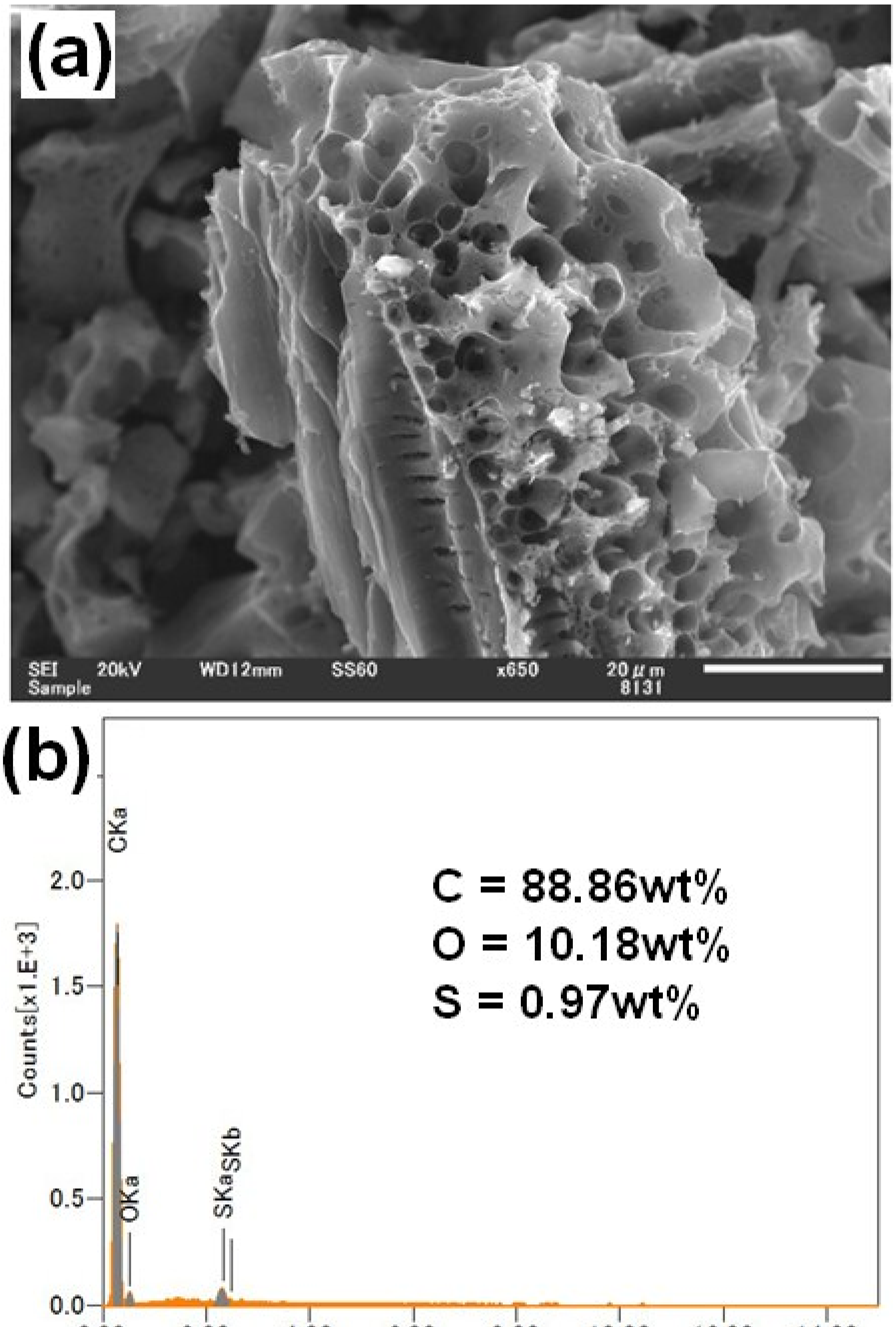

Fig. S9. (a) SEM image and (b) corresponding EDS spectrum of HMPC.

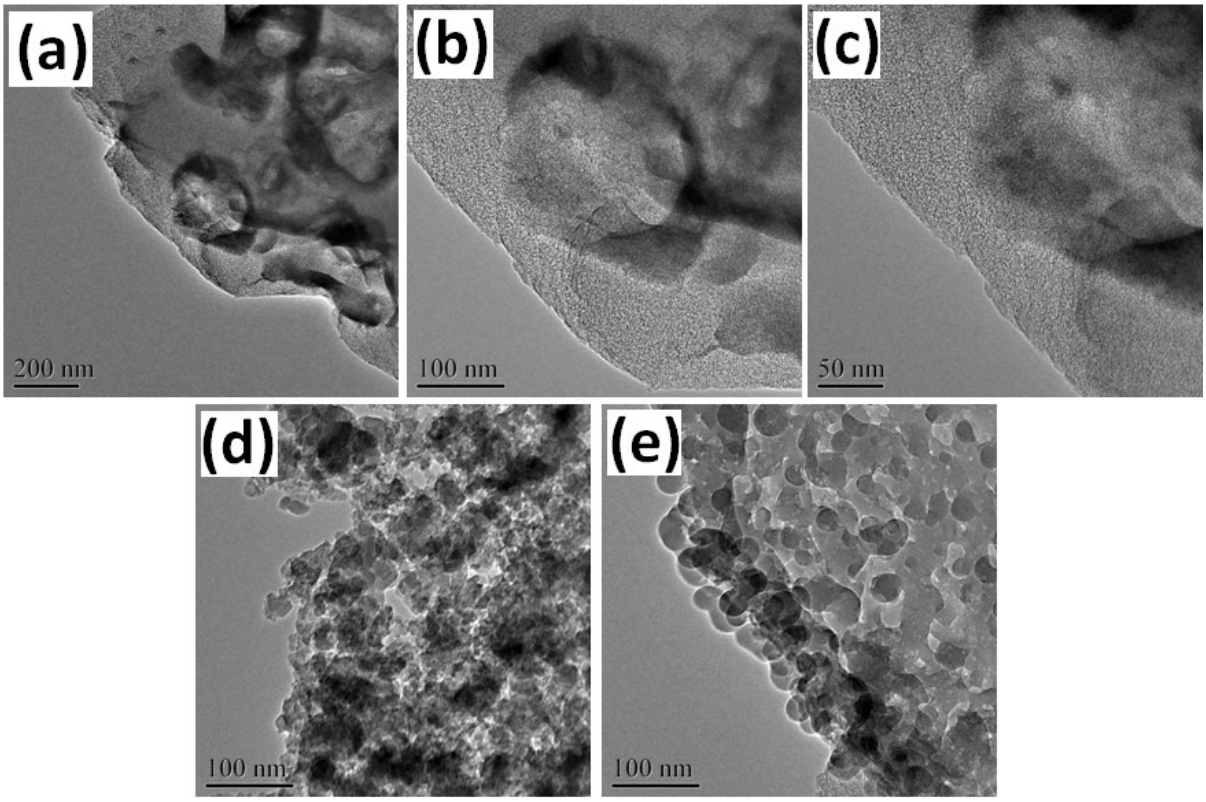

Fig. S10. TEM images of Cu/HMPC-1.

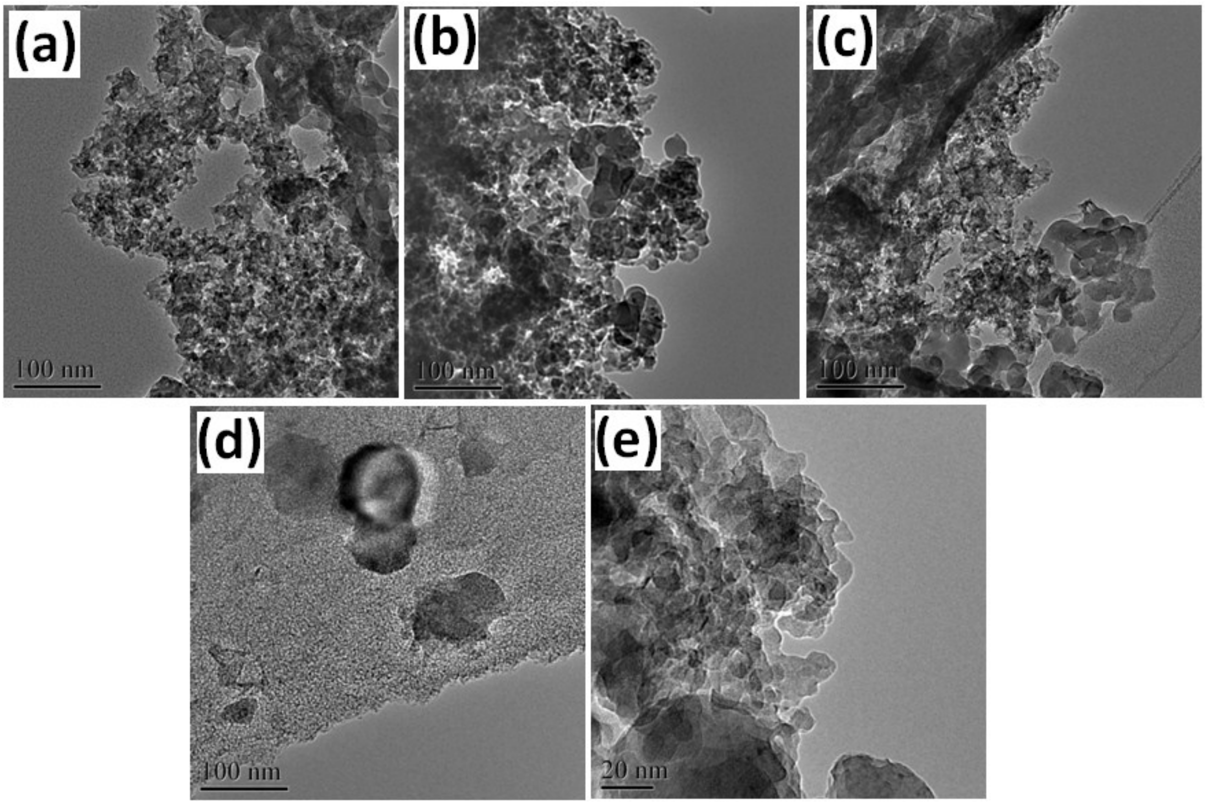

Fig. S11. TEM images of HMPC-1.

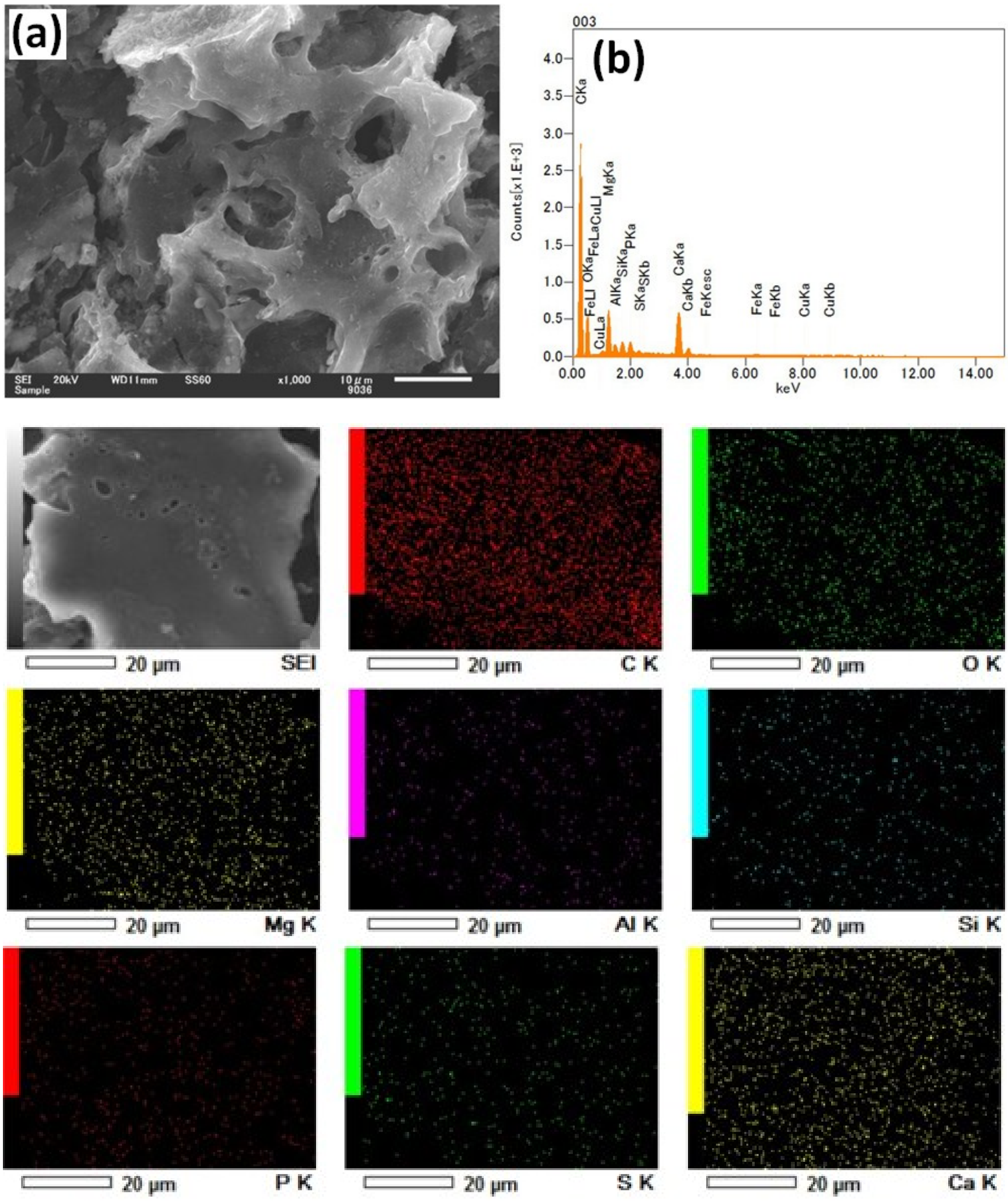

Fig. S12. SEM, EDS and corresponding elemental mapping of Cu/HMPC-1.

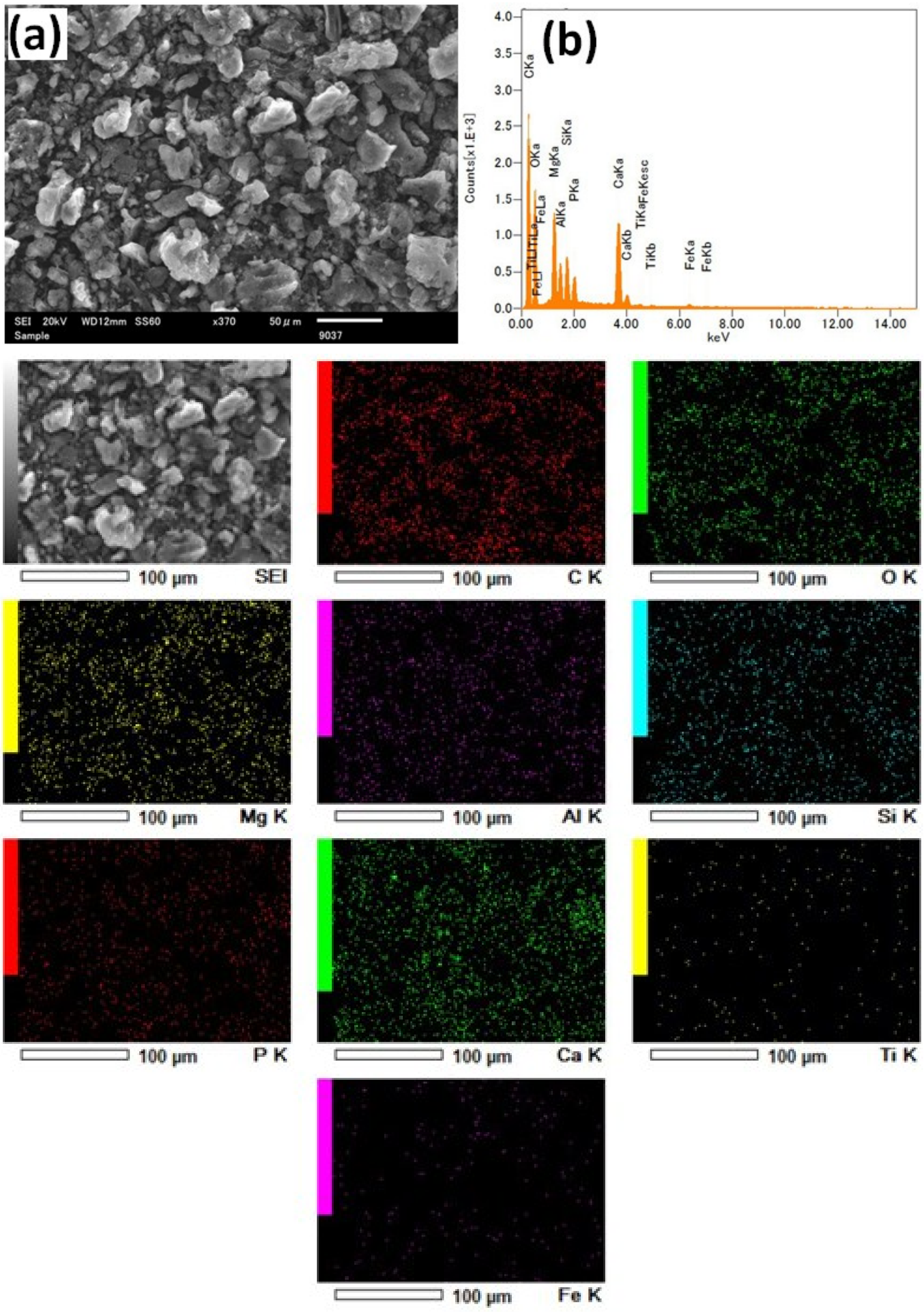

Fig. S13. SEM, EDS and corresponding elemental mapping of HMPC-1.

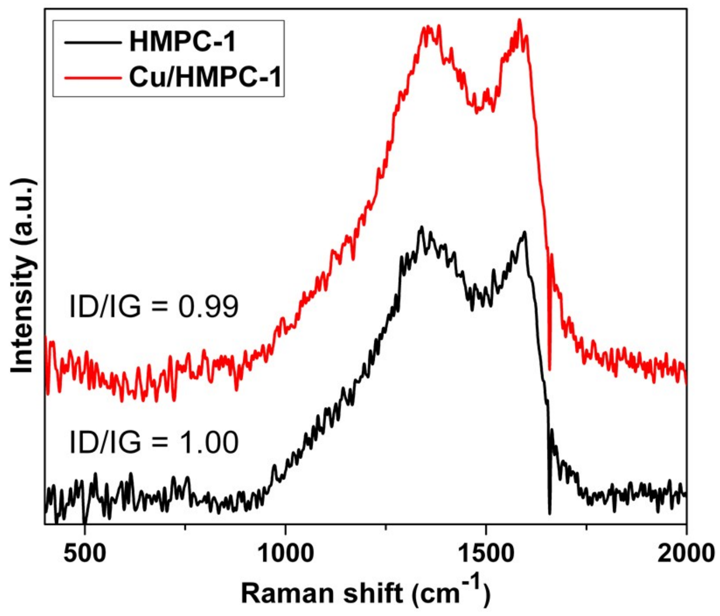

Fig. S14. Raman spectra of HMPC-1 and Cu/HMPC-1.

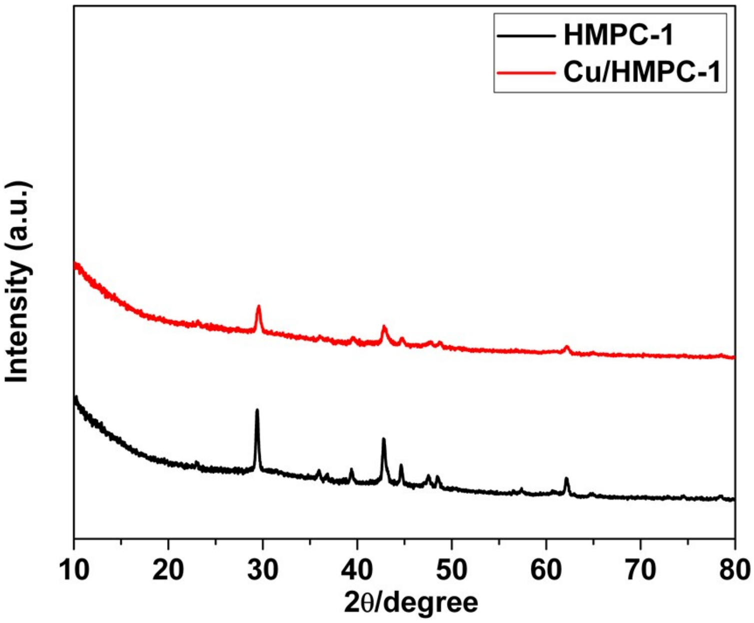

Fig. S15. XRD patterns of HMPC-1 and Cu/HMPC-1.

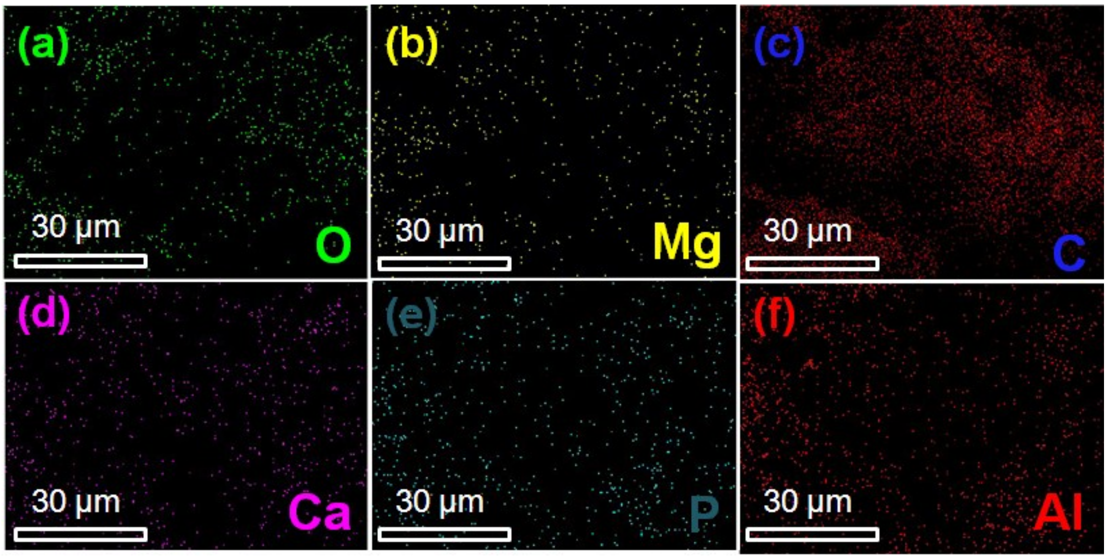

Fig. S16. EDS elemental mappings of used Cu/HMPC.

^{1}H NMR spectra, ^{13}C NMR spectra, GC chromatogram, MS(GC) spectra of catalytic products

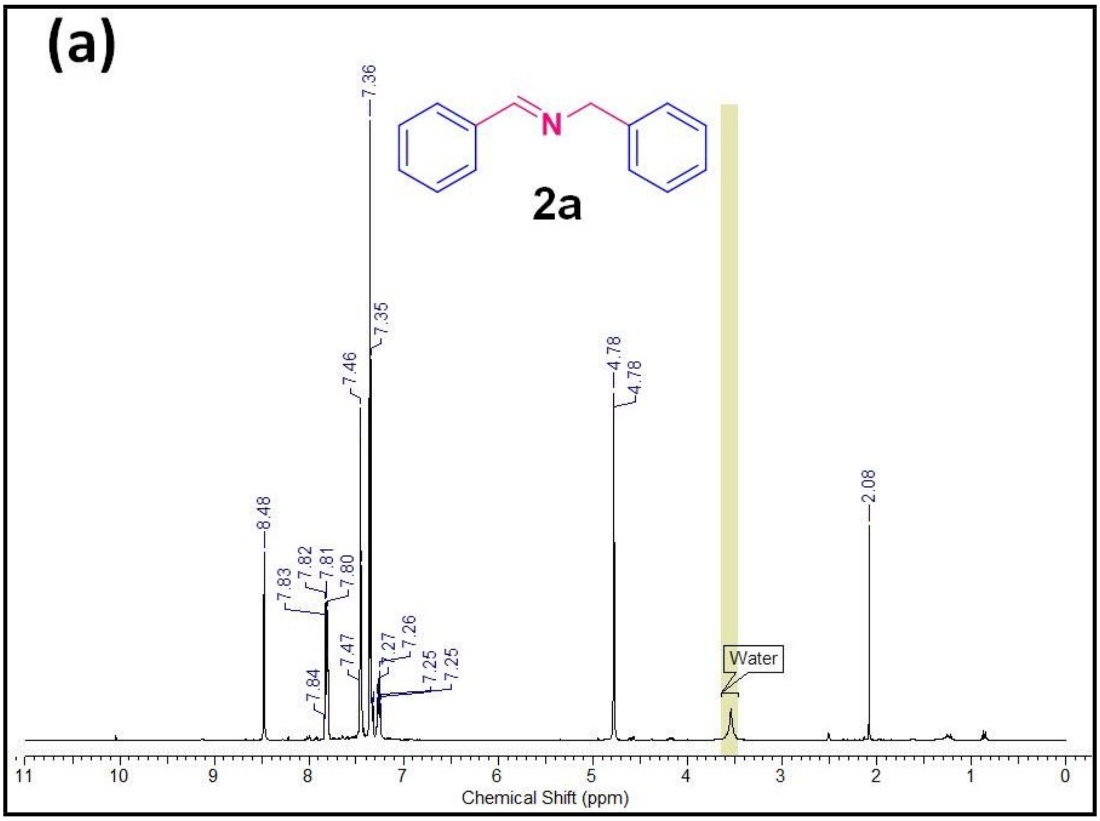

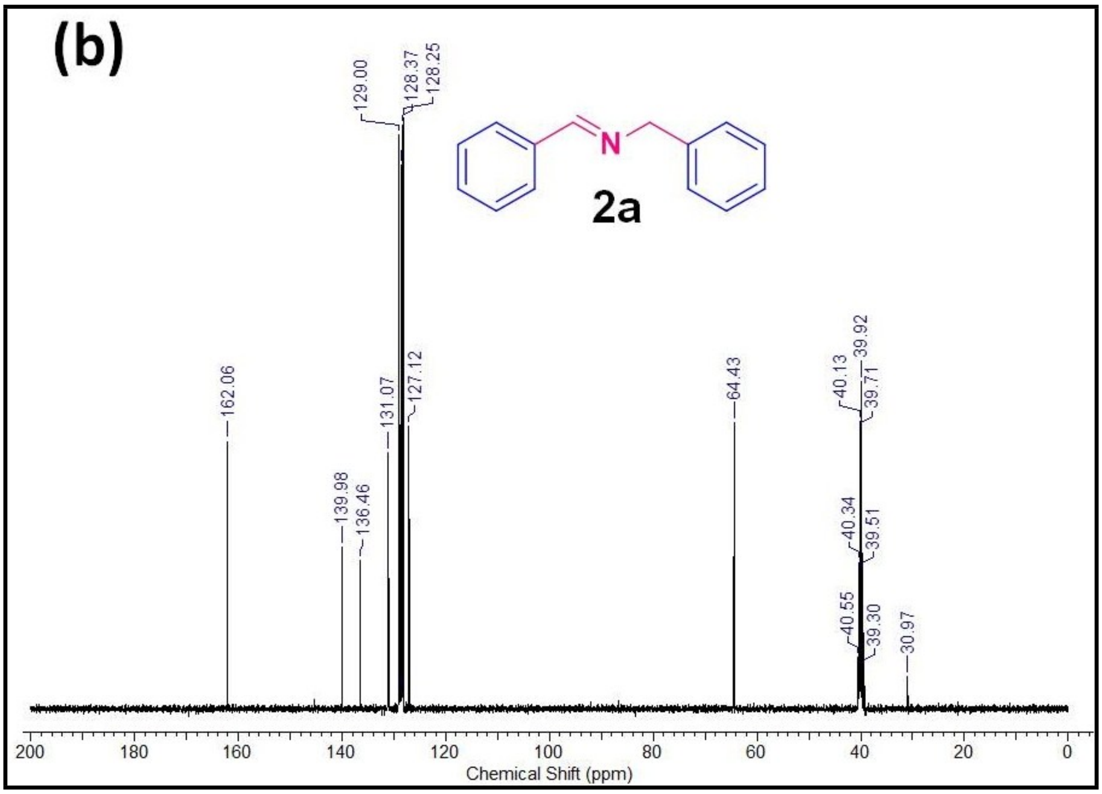

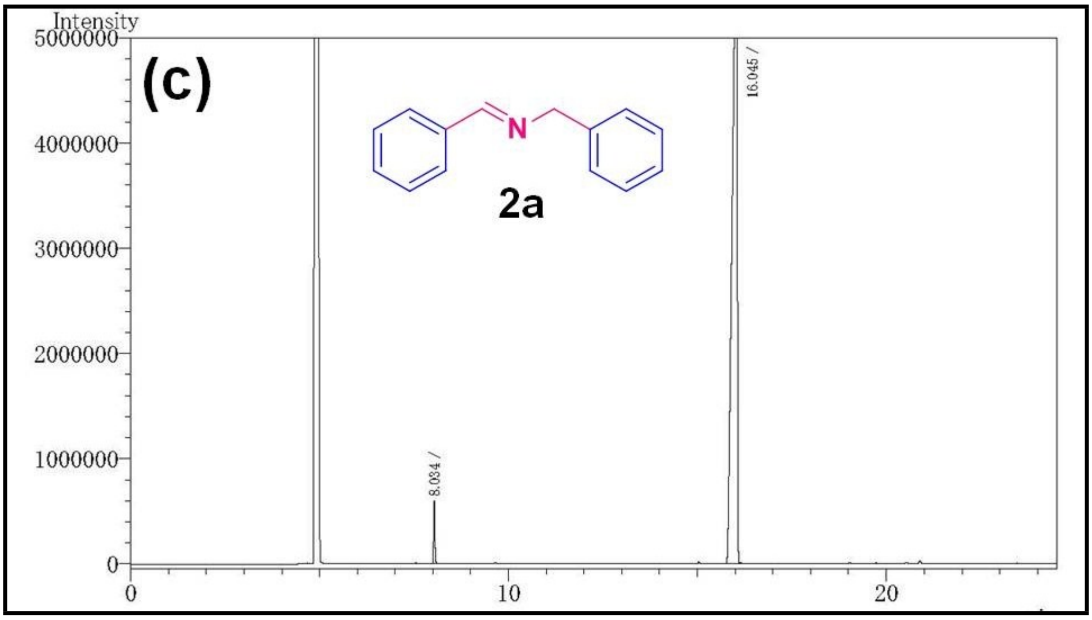

Fig. S17. (a) ^{1}H NMR spectrum, (b) ^{13}C NMR spectrum and (c) GC chromatogram of *N*-benzylidene-1-phenylmethanamine (**2a**).

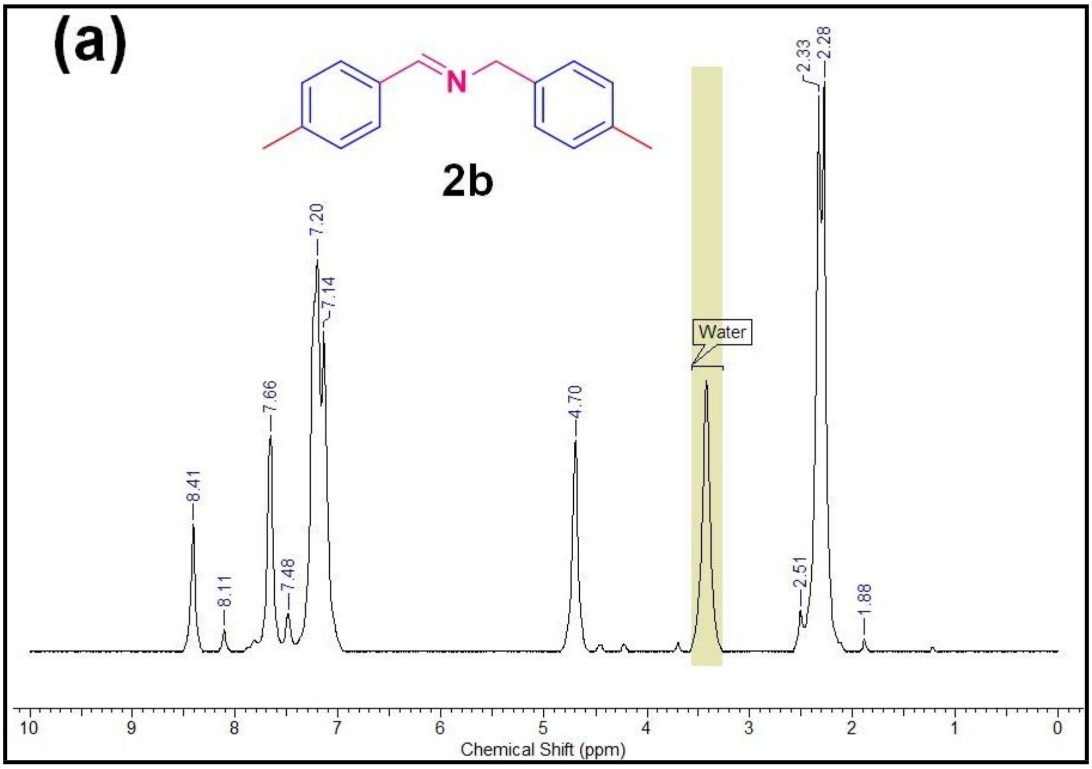
(a)
N
2b
8.41
8.11
7.66
7.48
7.20
7.14
4.70
Water
2.51
2.33
2.28
1.88
10 9 8 7 6 5 4 3 2 1 0
Chemical Shift (ppm)

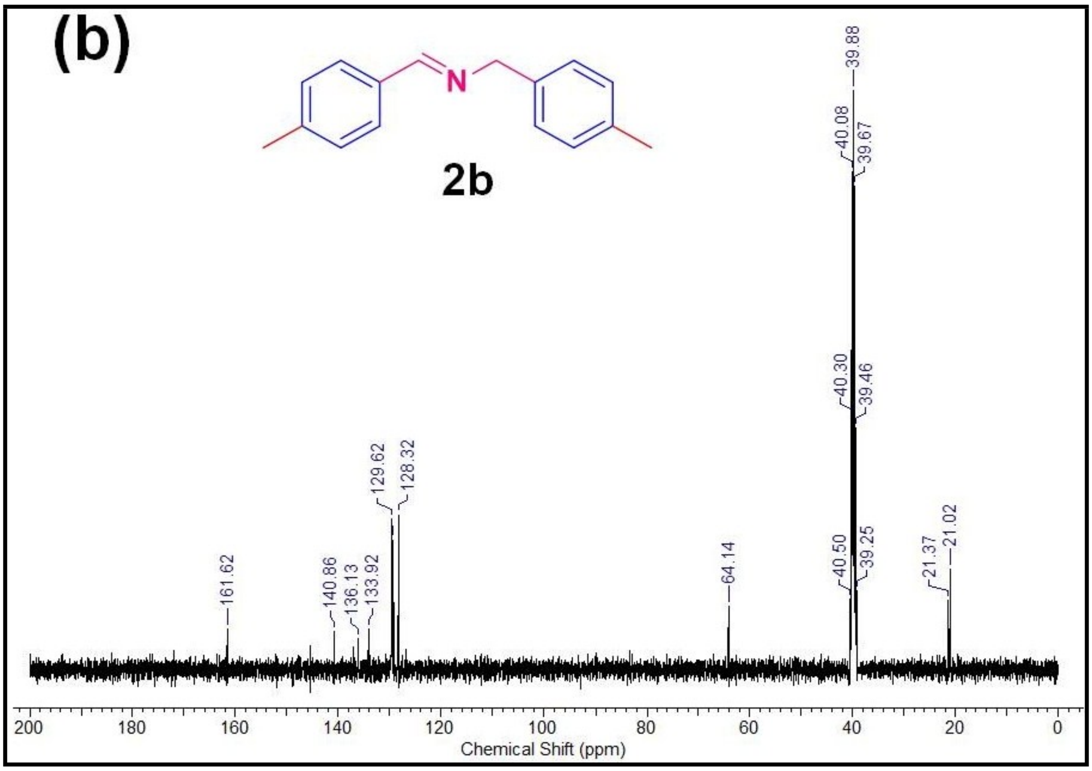
(b)
N
2b
161.62
140.86
136.13
133.92
129.62
128.32
64.14
40.50
40.30
40.08
39.88
39.67
39.46
39.25
21.37
21.02
200 180 160 140 120 100 80 60 40 20 0
Chemical Shift (ppm)

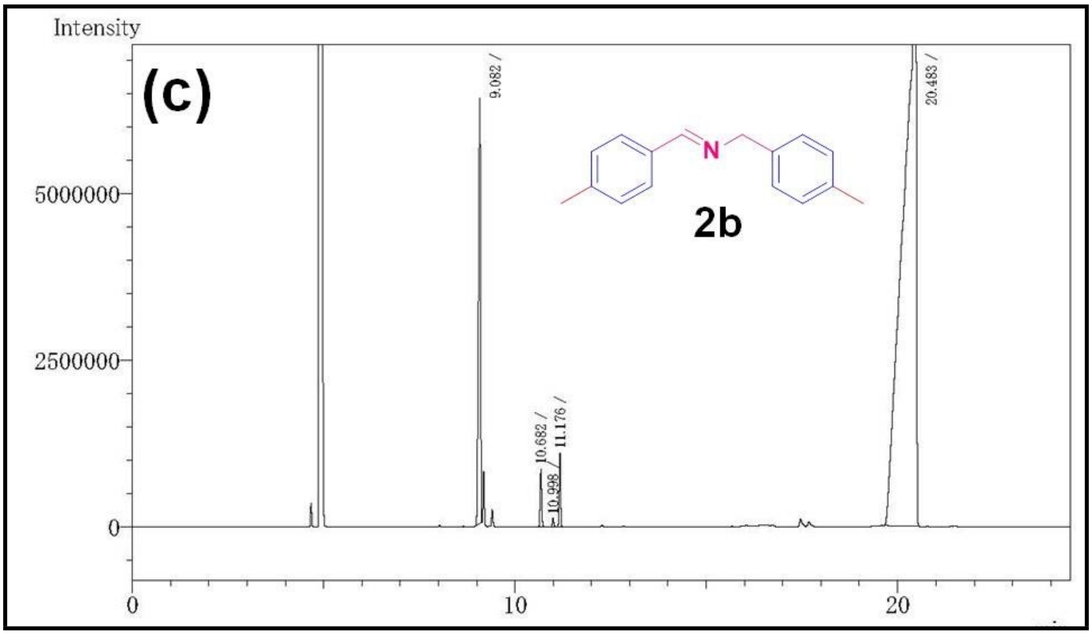

Fig. S18. (a) ^{1}H NMR spectrum, (b) ^{13}C NMR spectrum and (c) GC chromatogram of *N*-(4-methylbenzylidene)-1-(4-tolyl)methanamine (**2b**).

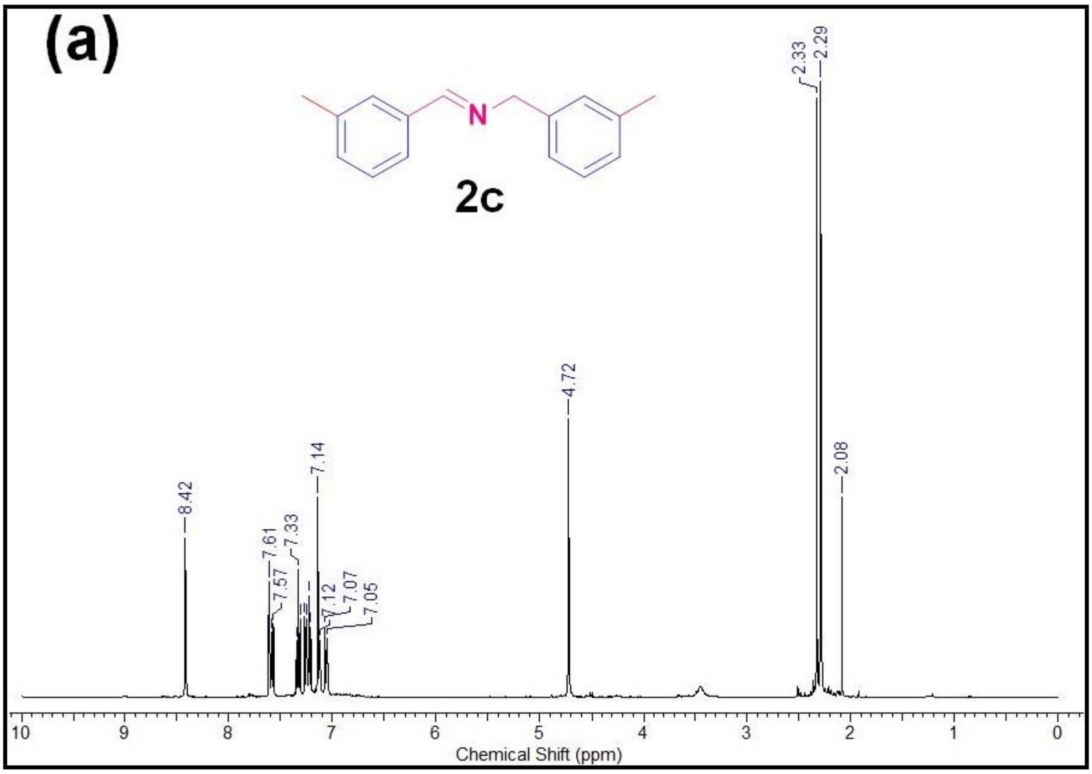
(a)
N
2c
8.42
7.61
7.57
7.33
7.14
7.12
7.07
7.05
4.72
2.33
2.29
2.08
10 9 8 7 6 5 4 3 2 1 0
Chemical Shift (ppm)

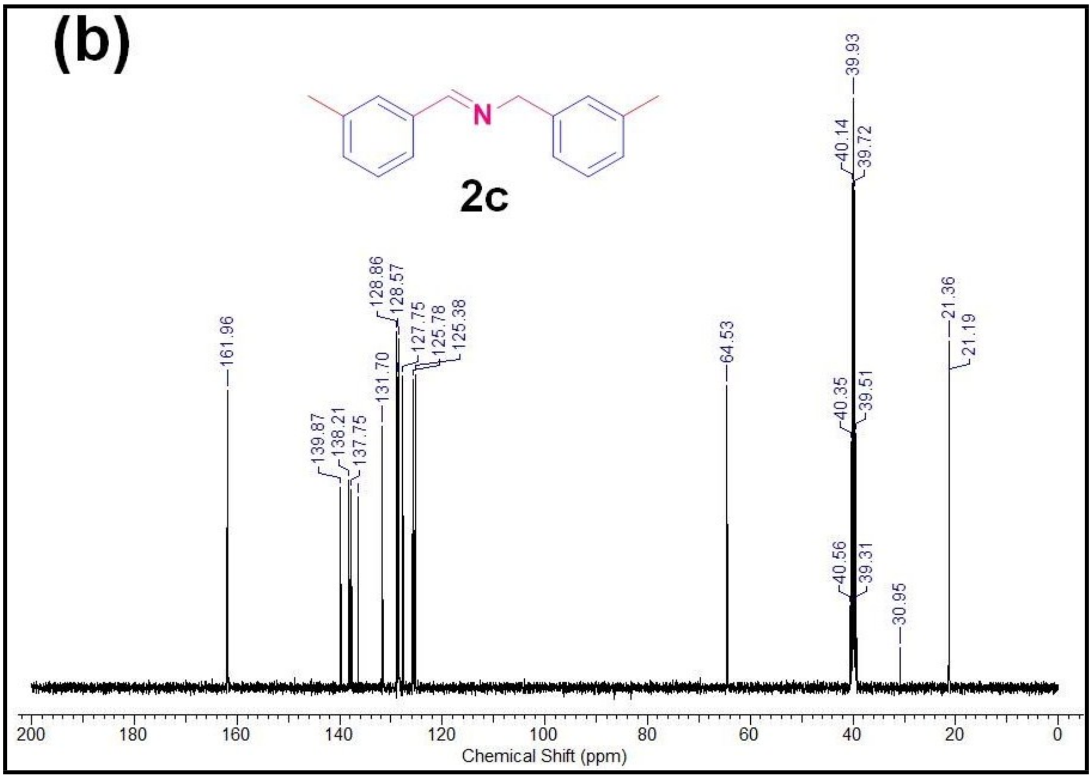
(b)
N
2c
161.96
139.87
138.21
137.75
131.70
128.86
128.57
127.75
125.78
125.38
64.53
40.56
40.35
40.14
39.93
39.72
39.51
39.31
30.95
21.36
21.19
200 180 160 140 120 100 80 60 40 20 0
Chemical Shift (ppm)

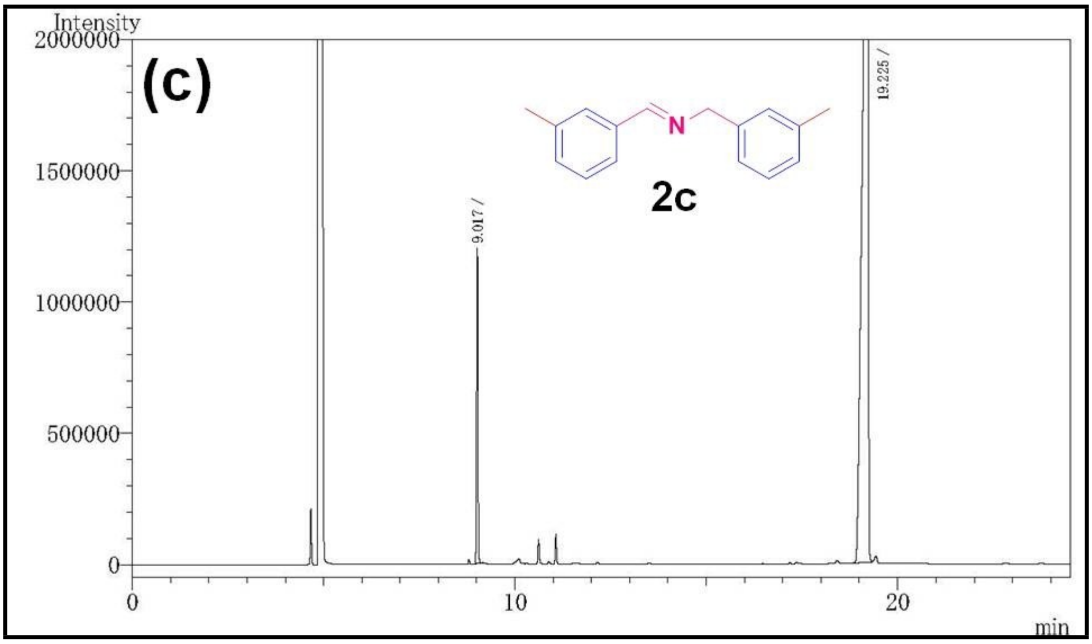

Fig. S19 (a) ^{1}H NMR spectrum, (b) ^{13}C NMR spectrum and (c) GC chromatogram of *N*-(3-methylbenzyl)-1-(3-tolyl)methanimine (**2c**).

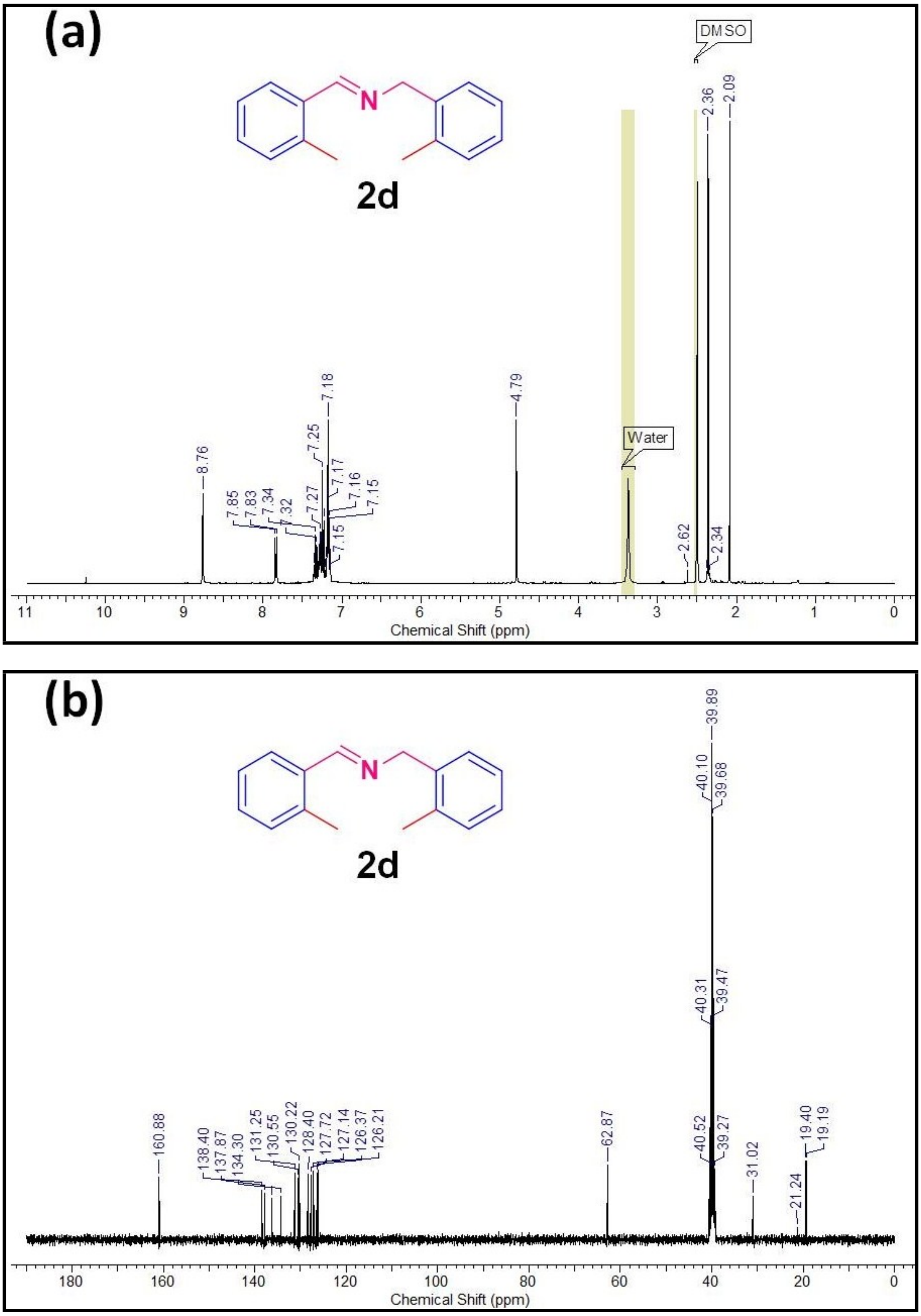
(a)
N
2d
DMSO
Water
8.76
7.85
7.83
7.34
7.32
7.27
7.25
7.18
7.17
7.16
7.15
7.15
4.79
2.62
2.36
2.34
2.09
11 10 9 8 7 6 5 4 3 2 1 0
Chemical Shift (ppm)
(b)
N
2d
160.88
138.40
137.87
134.30
131.25
130.55
130.22
128.40
127.72
127.14
126.37
126.21
62.87
40.52
40.31
40.10
39.89
39.68
39.47
39.27
31.02
21.24
19.40
19.19
180 160 140 120 100 80 60 40 20 0
Chemical Shift (ppm)

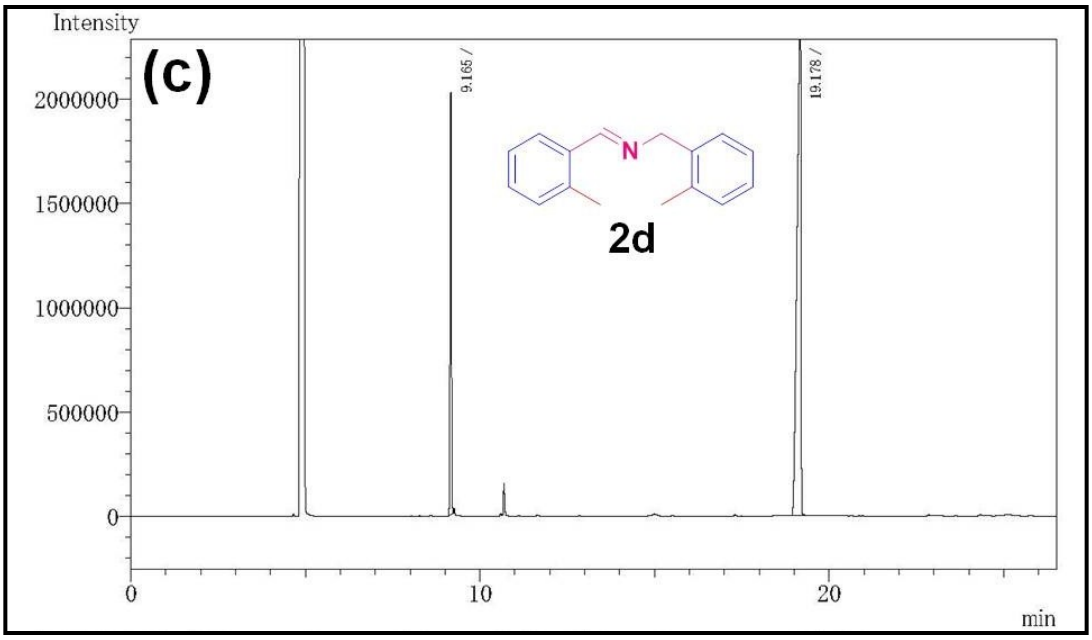

Fig. S20. (a) ^{1}H NMR spectrum, (b) ^{13}C NMR spectrum and (c) GC chromatogram of *N*-(2-methylbenzylidene)-2-methylbenzylamine (**2d**).

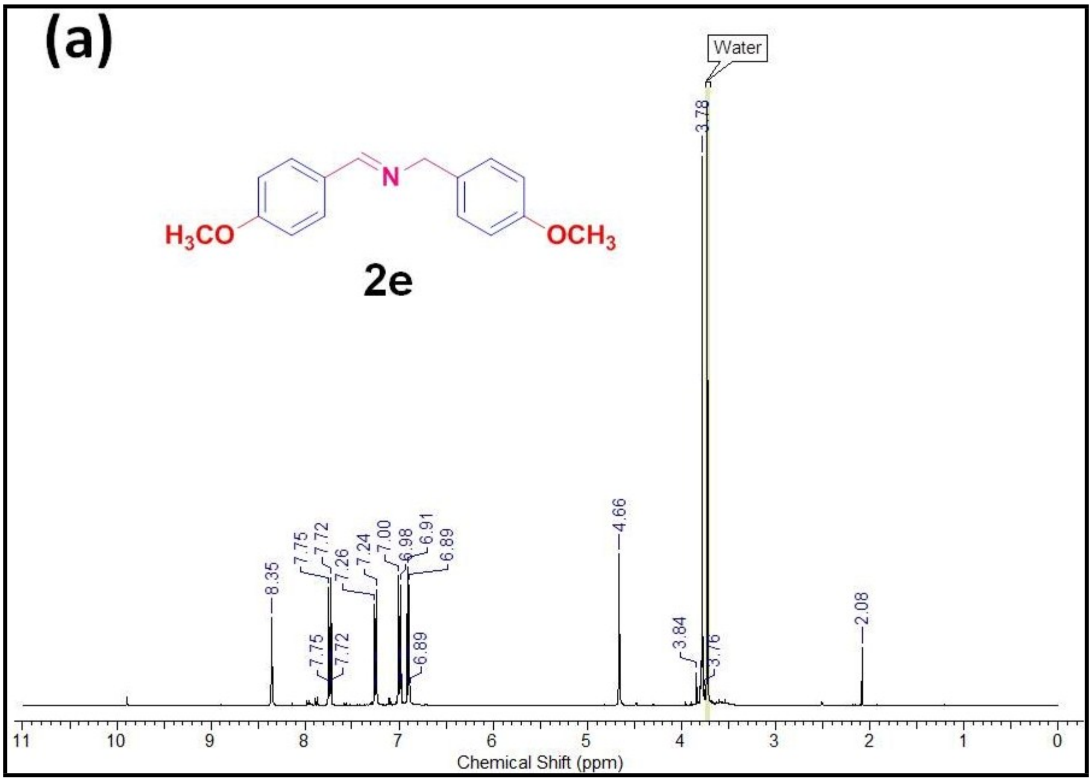
(a)
Water
N
H3CO
OCH3
2e
8.35
7.75
7.72
7.26
7.24
7.00
6.98
6.91
6.89
7.75
7.72
6.89
4.66
3.78
3.84
3.76
2.08
11 10 9 8 7 6 5 4 3 2 1 0
Chemical Shift (ppm)

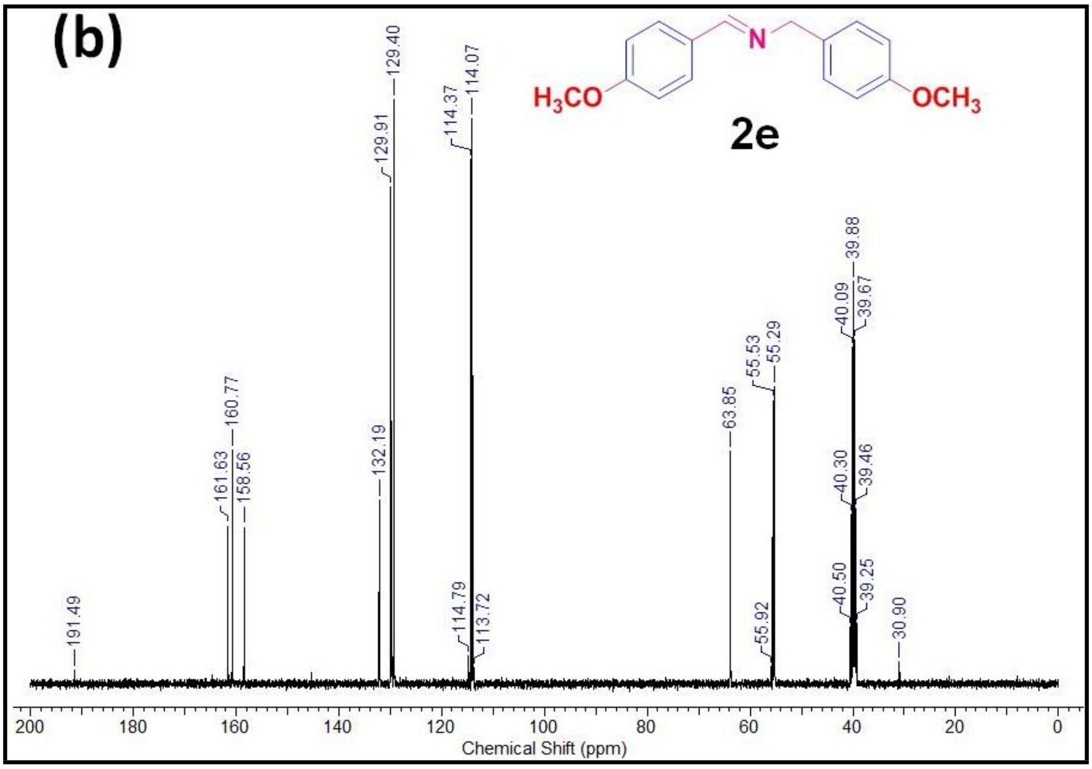
(b)
N
H3CO
OCH3
2e
191.49
161.63
160.77
158.56
132.19
129.91
129.40
114.37
114.07
114.79
113.72
63.85
55.53
55.29
55.92
40.09
39.88
39.67
40.30
39.46
40.50
39.25
30.90
200 180 160 140 120 100 80 60 40 20 0
Chemical Shift (ppm)

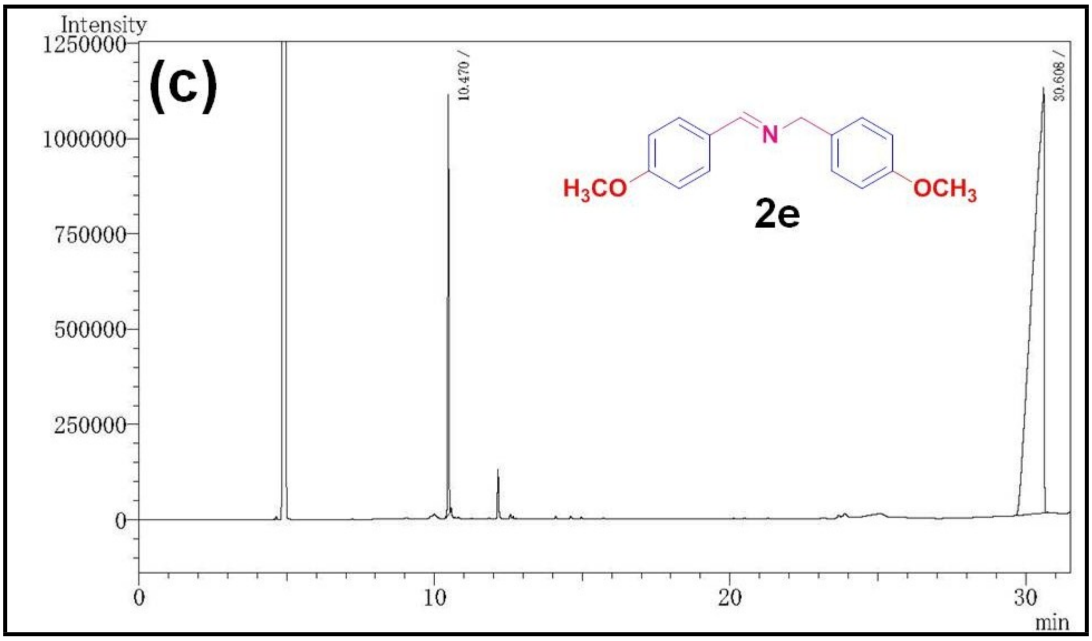

Fig. S21. (a) ^{1}H NMR spectrum, (b) ^{13}C NMR spectrum and (c) GC chromatogram of *N*-(4-methoxybenzylidene)-1-(4-methoxyphenyl)methanamine (**2e**).

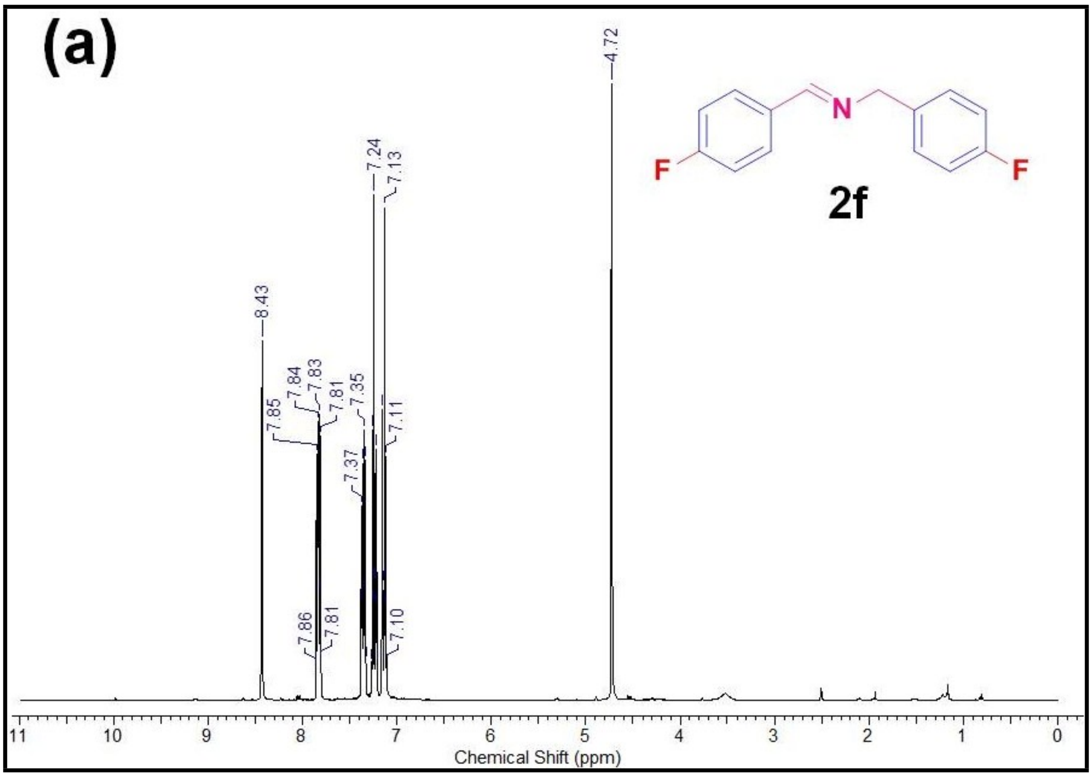
(a)
4.72
7.24
7.13
8.43
7.85
7.84
7.83
7.81
7.35
7.11
7.37
7.86
7.81
7.10
F
N
F
2f
11 10 9 8 7 6 5 4 3 2 1 0
Chemical Shift (ppm)

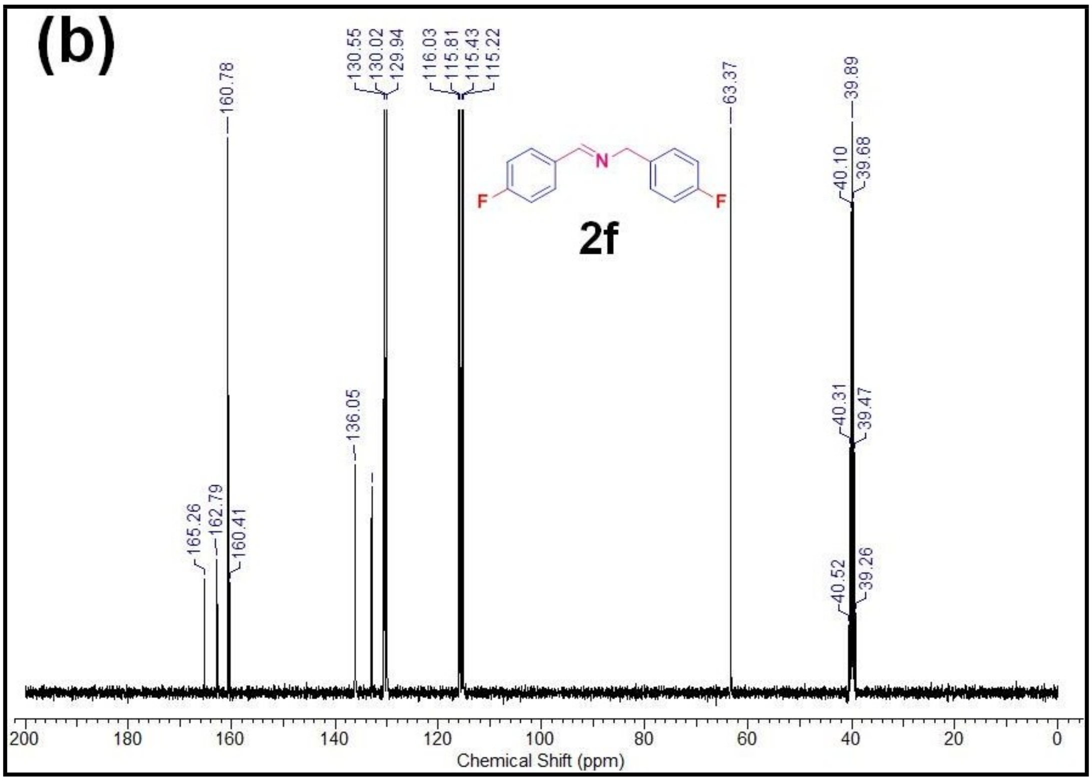
(b)
160.78
130.55
130.02
129.94
116.03
115.81
115.43
115.22
63.37
39.89
40.10
39.68
136.05
40.31
39.47
165.26
162.79
160.41
40.52
39.26
F
N
F
2f
200 180 160 140 120 100 80 60 40 20 0
Chemical Shift (ppm)

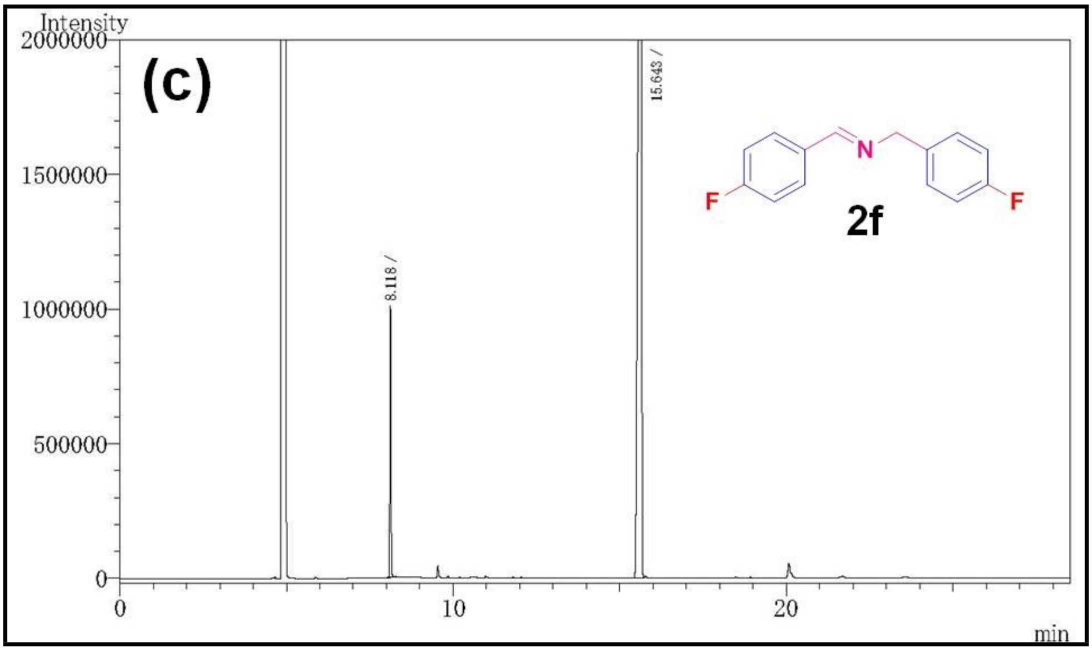

Fig. S22. (a) ^{1}H NMR spectrum, (b) ^{13}C NMR spectrum and (c) GC chromatogram of *N*-(4-fluorobenzylidene)-1-(4-fluorophenyl)methanamine (**2f**).

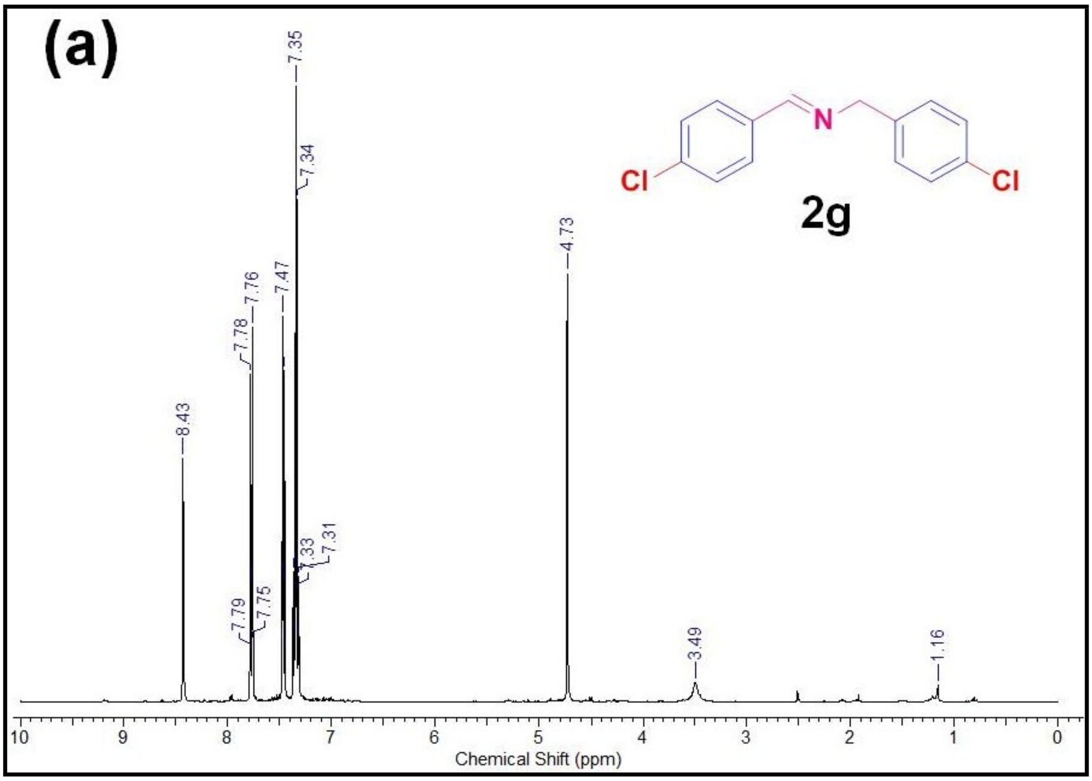
(a)
2g
Cl
N
Cl
8.43
7.79
7.78
7.76
7.75
7.47
7.35
7.34
7.33
7.31
4.73
3.49
1.16
10
9
8
7
6
5
4
3
2
1
0
Chemical Shift (ppm)

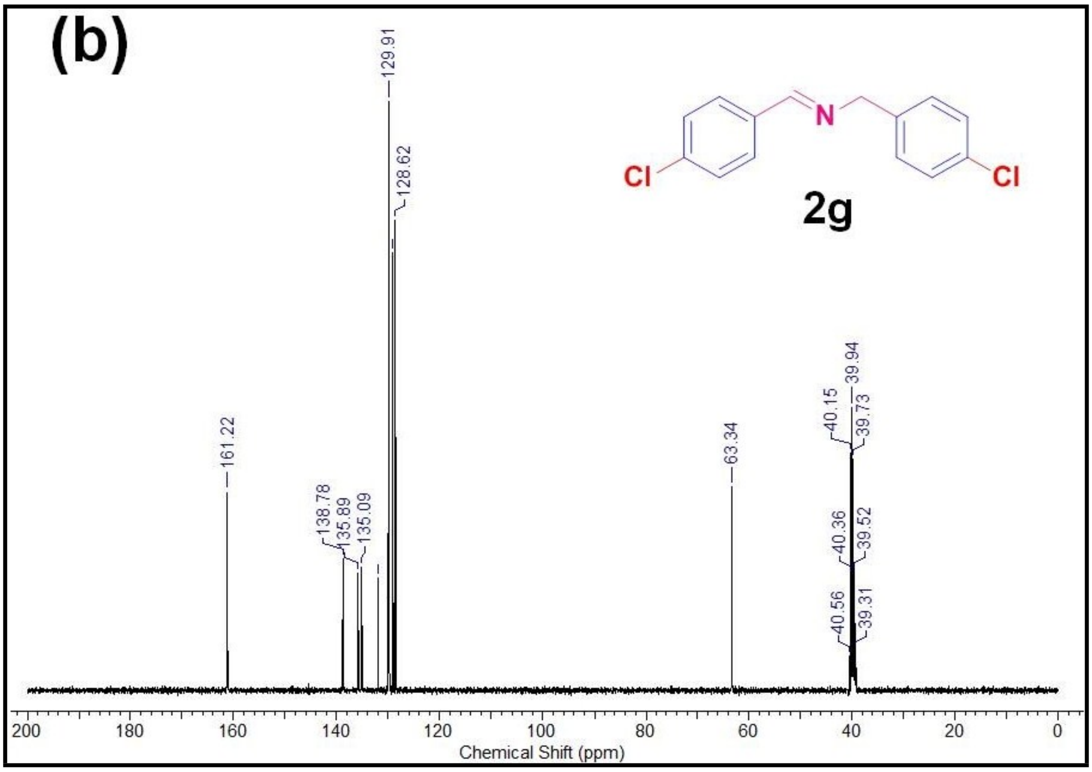
(b)
2g
Cl
N
Cl
161.22
138.78
135.89
135.09
129.91
128.62
63.34
40.56
40.36
40.15
39.94
39.73
39.52
39.31
200
180
160
140
120
100
80
60
40
20
0
Chemical Shift (ppm)

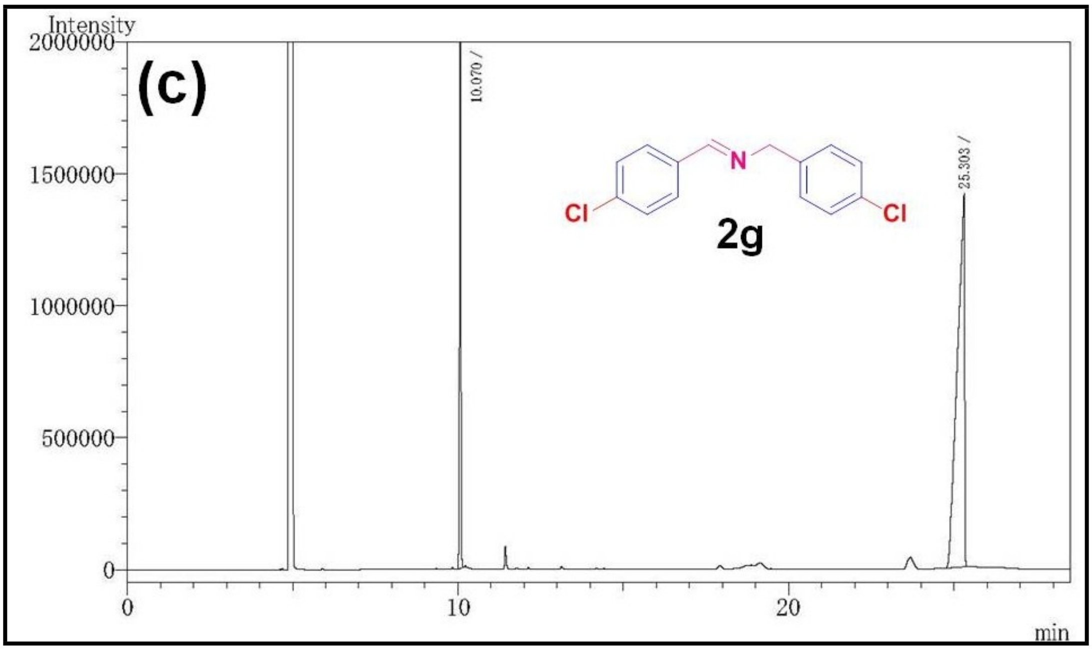

Fig. S23. (a) ^{1}H NMR spectrum, (b) ^{13}C NMR spectrum and (c) GC chromatogram of *N*-(4-Chlorobenzylidene)-1-(4-chlorophenyl)methanamine (**2g**).

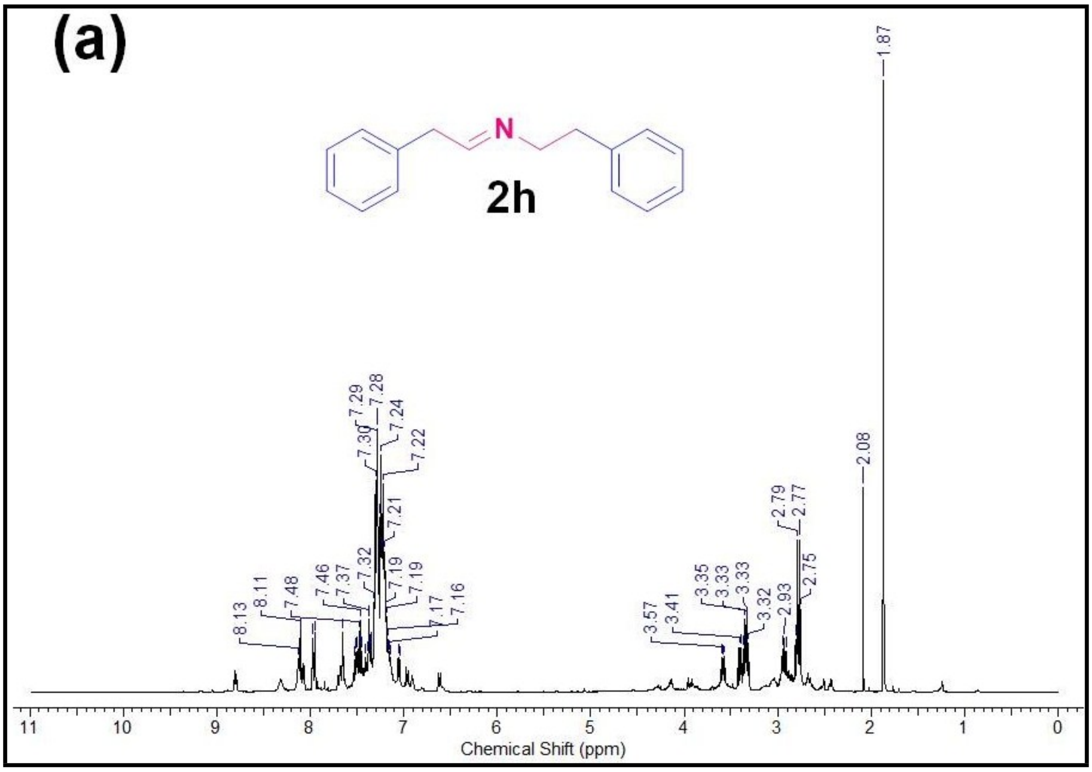
(a)
N
2h
8.13
8.11
7.48
7.46
7.37
7.32
7.30
7.29
7.28
7.24
7.22
7.21
7.19
7.19
7.17
7.16
3.57
3.41
3.35
3.33
3.33
3.32
2.93
2.79
2.77
2.75
2.08
1.87
Chemical Shift (ppm)

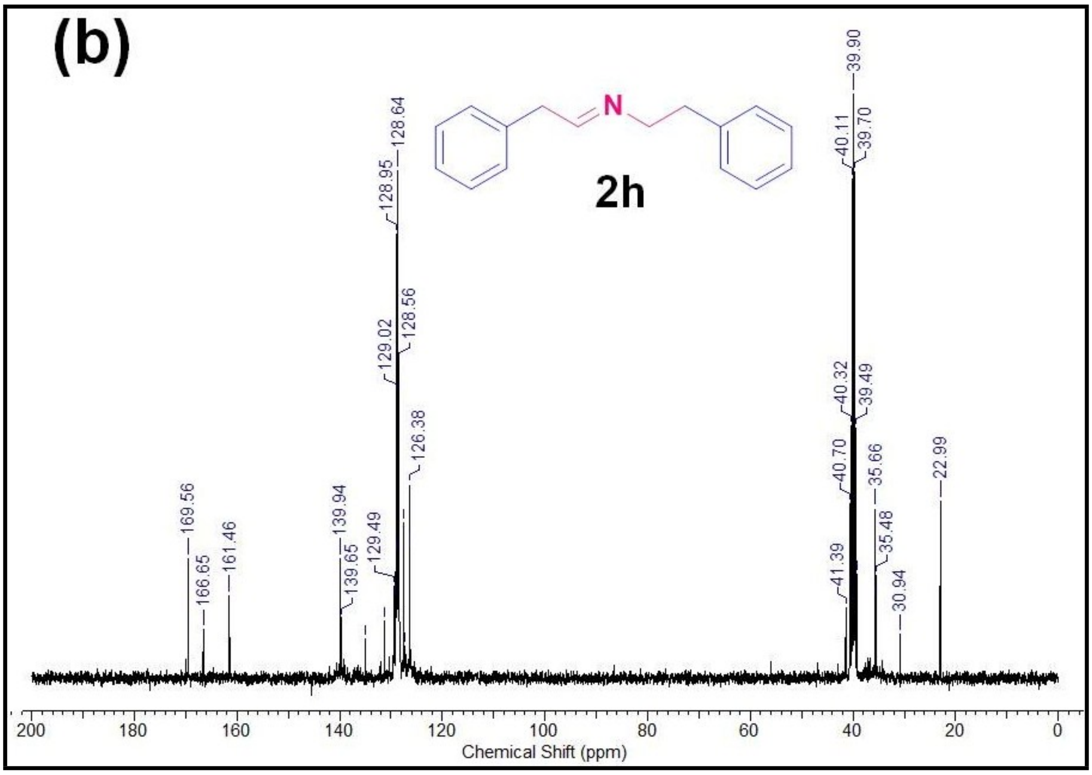
(b)
N
2h
169.56
166.65
161.46
139.94
139.65
129.49
129.02
128.95
128.64
128.56
126.38
41.39
40.70
40.32
40.11
39.90
39.70
39.49
35.66
35.48
30.94
22.99
Chemical Shift (ppm)

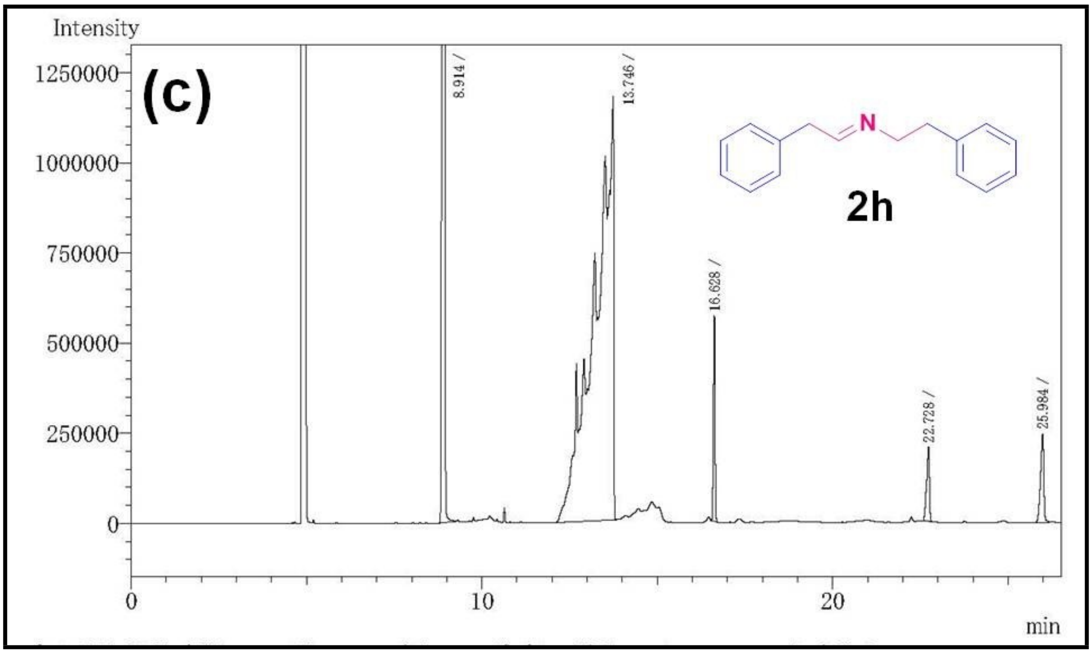

Fig. S24. (a) ^{1}H NMR spectrum, (b) ^{13}C NMR spectrum and (c) GC chromatogram of 2-phenyl-*N*-(2-phenylethylidene)ethanamine (**2h**).

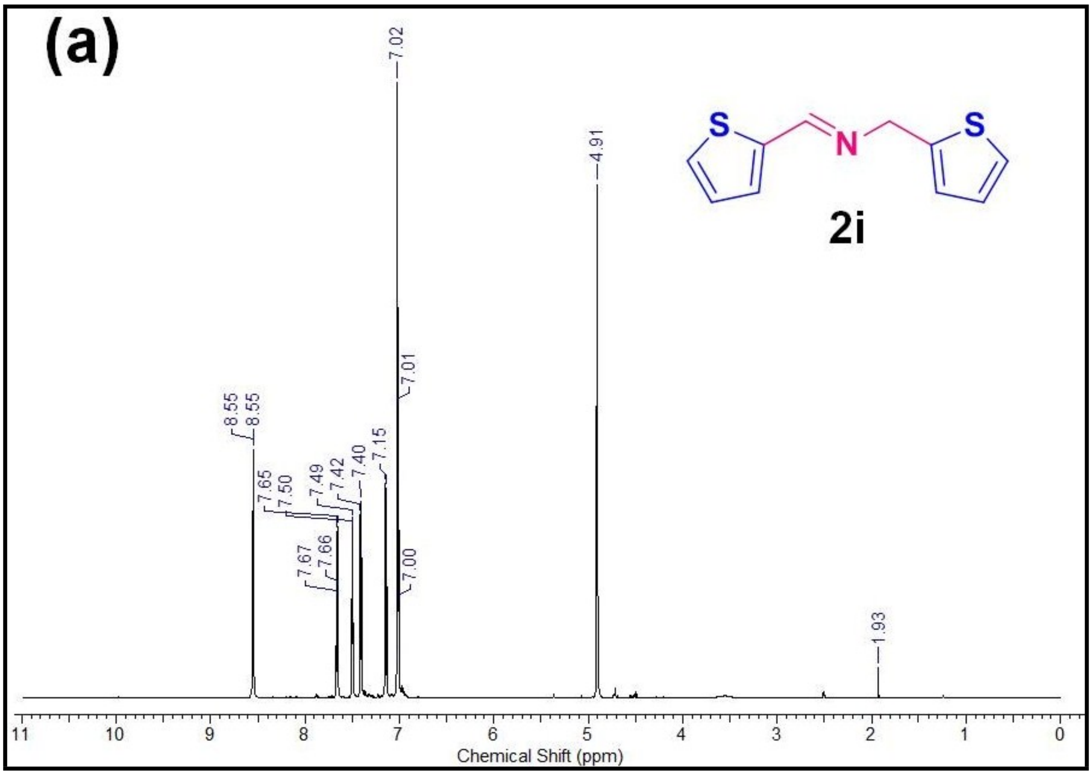
(a)
2i
S
N
S
7.02
4.91
7.01
8.55
8.55
7.65
7.50
7.49
7.42
7.40
7.15
7.67
7.66
7.00
1.93
11 10 9 8 7 6 5 4 3 2 1 0
Chemical Shift (ppm)

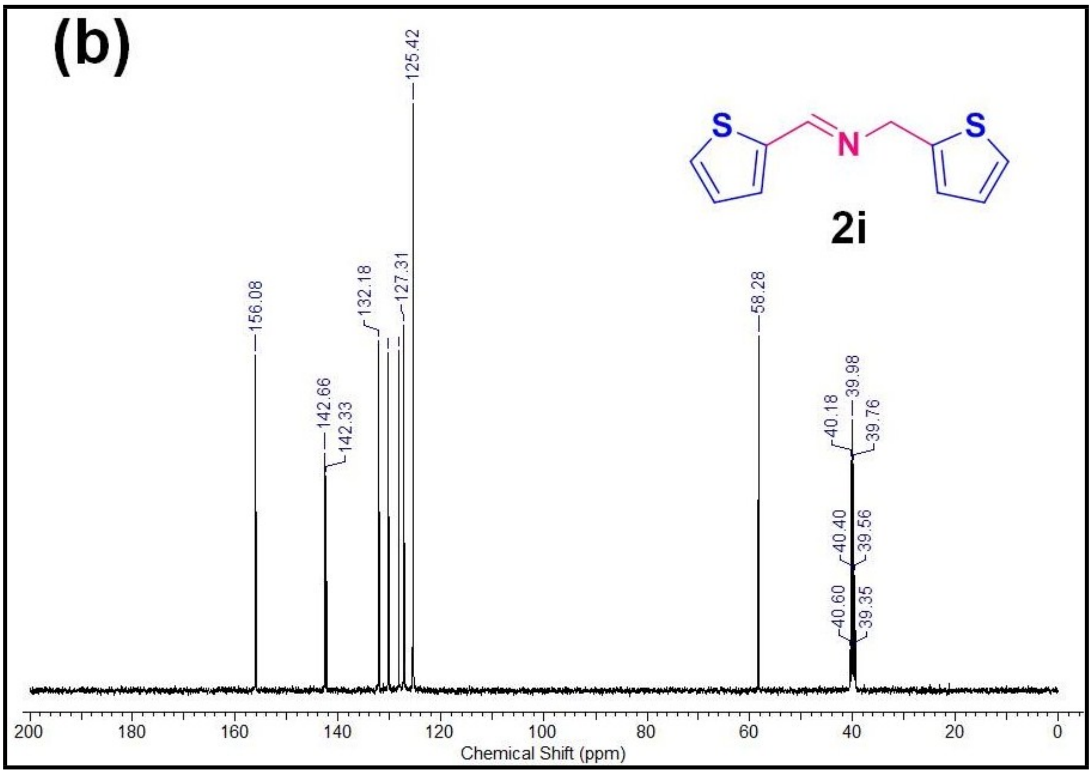
(b)
2i
S
N
S
125.42
156.08
132.18
127.31
58.28
142.66
142.33
40.18
39.98
39.76
40.40
39.56
40.60
39.35
200 180 160 140 120 100 80 60 40 20 0
Chemical Shift (ppm)

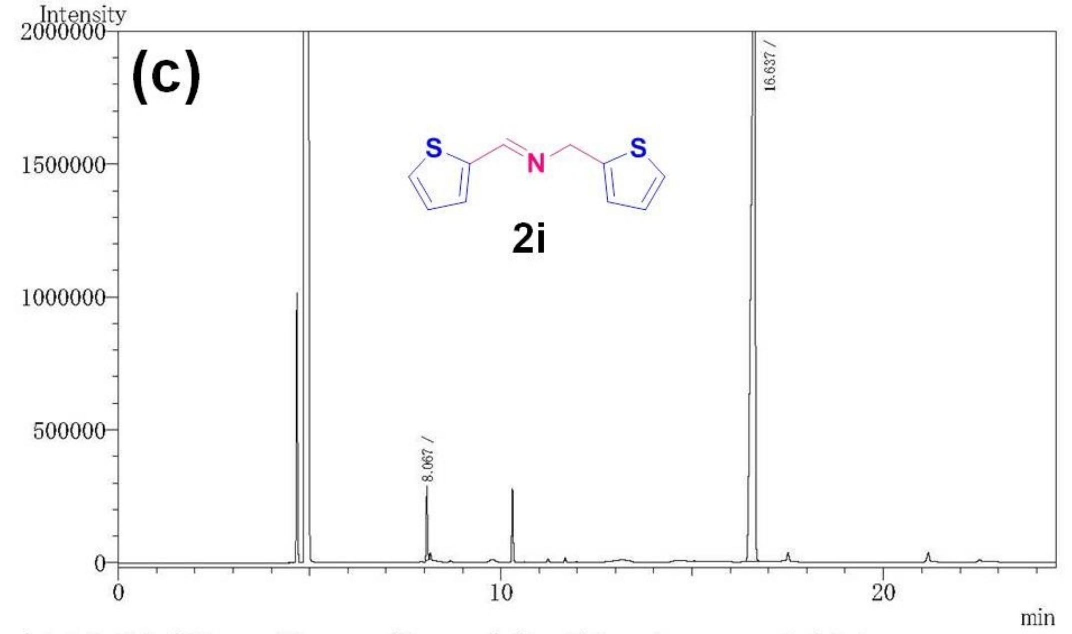

ピーク番号	保持時間	面積	高さ	濃度	単位	マーク	ID#	化合物名
1	8.067	629823	279419	3.365				
2	16.637	18087354	2673129	96.635				
合計		18717177	2952548					

Fig. S25. (a) ^{1}H NMR spectrum, (b) ^{13}C NMR spectrum and (c) GC chromatogram of *N*-(thiophen-2-yl)-1-(thiophen-2-yl)methanamine (**2i**).

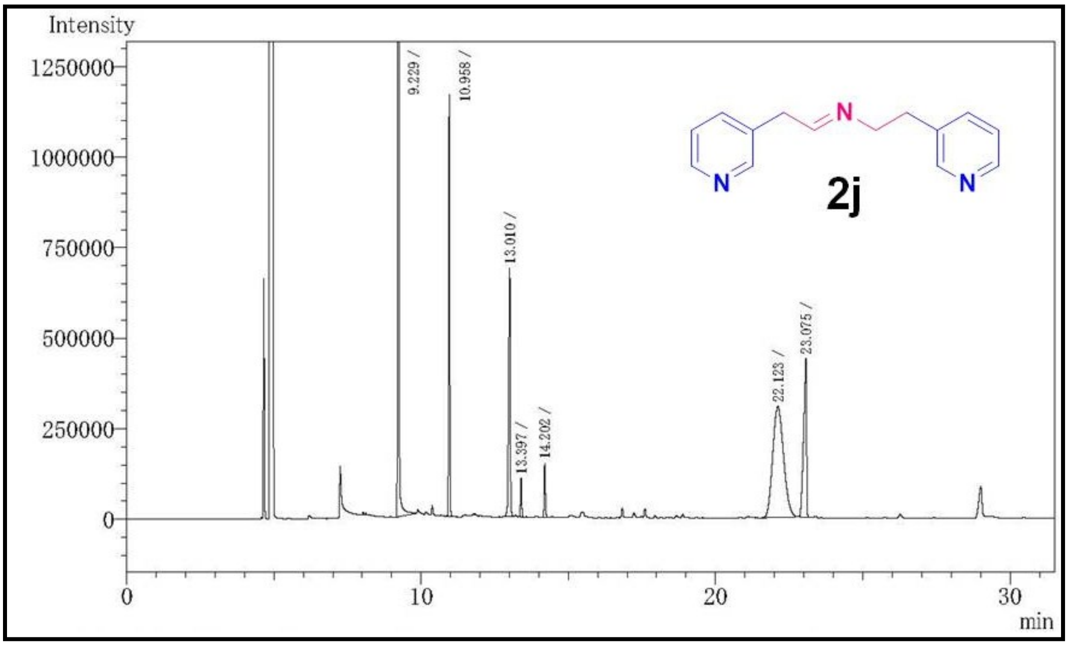

Fig. S26. GC chromatogram of 2-(pyridin-3-yl)-*N*-(2-(pyridin-3-yl)ethylidene)ethanamine (**2j**).

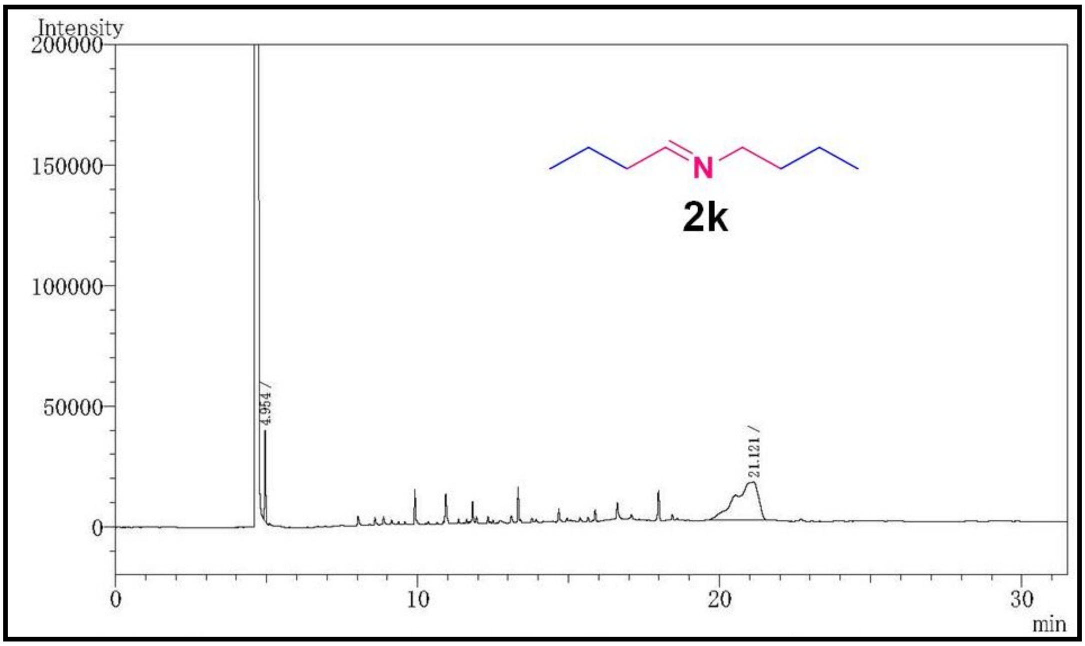

Fig. S27. GC chromatogram of *N*-butylidenebutan-1-amine (**2k**).

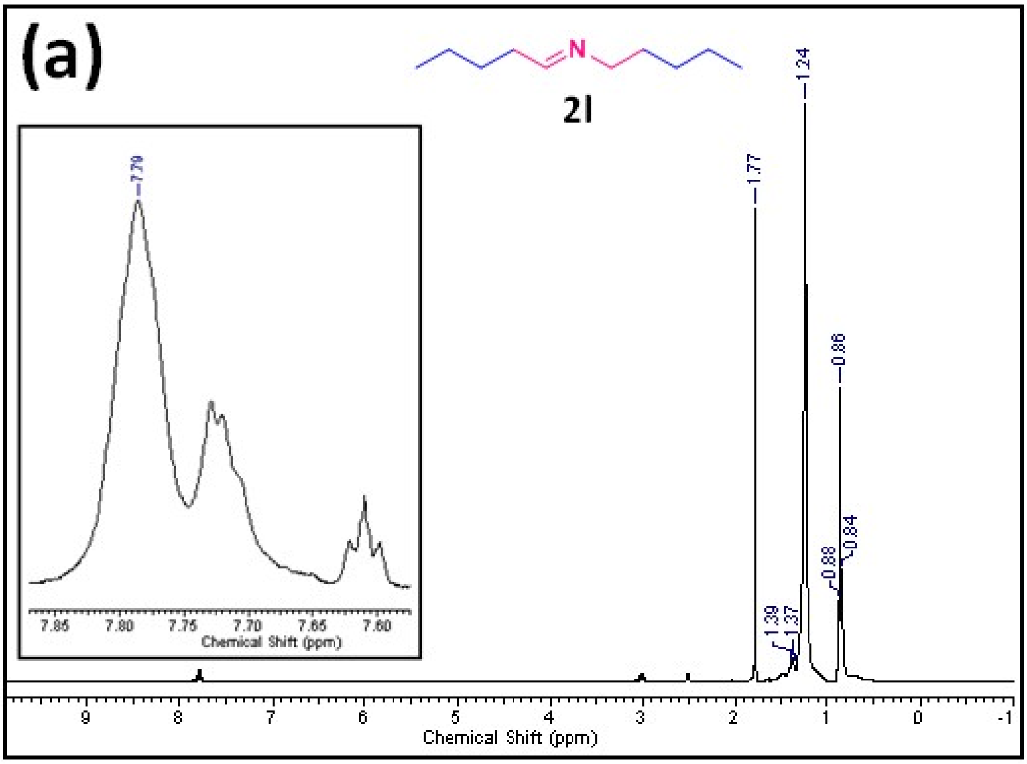
(a)
2l
7.79
1.77
1.24
0.86
0.88
0.84
1.39
1.37
7.85 7.80 7.75 7.70 7.65 7.60
Chemical Shift (ppm)
9 8 7 6 5 4 3 2 1 0 -1
Chemical Shift (ppm)

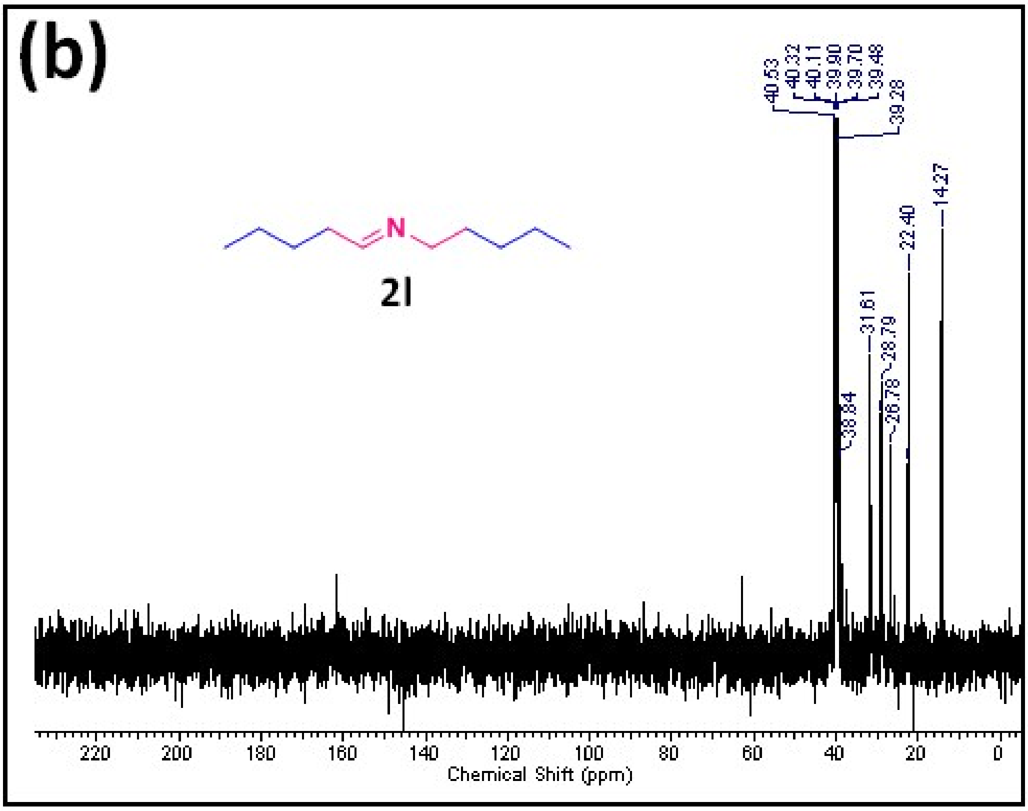
(b)
2l
40.53
40.32
40.11
39.90
39.70
39.48
39.28
38.84
31.61
28.79
26.78
22.40
14.27
220 200 180 160 140 120 100 80 60 40 20 0
Chemical Shift (ppm)

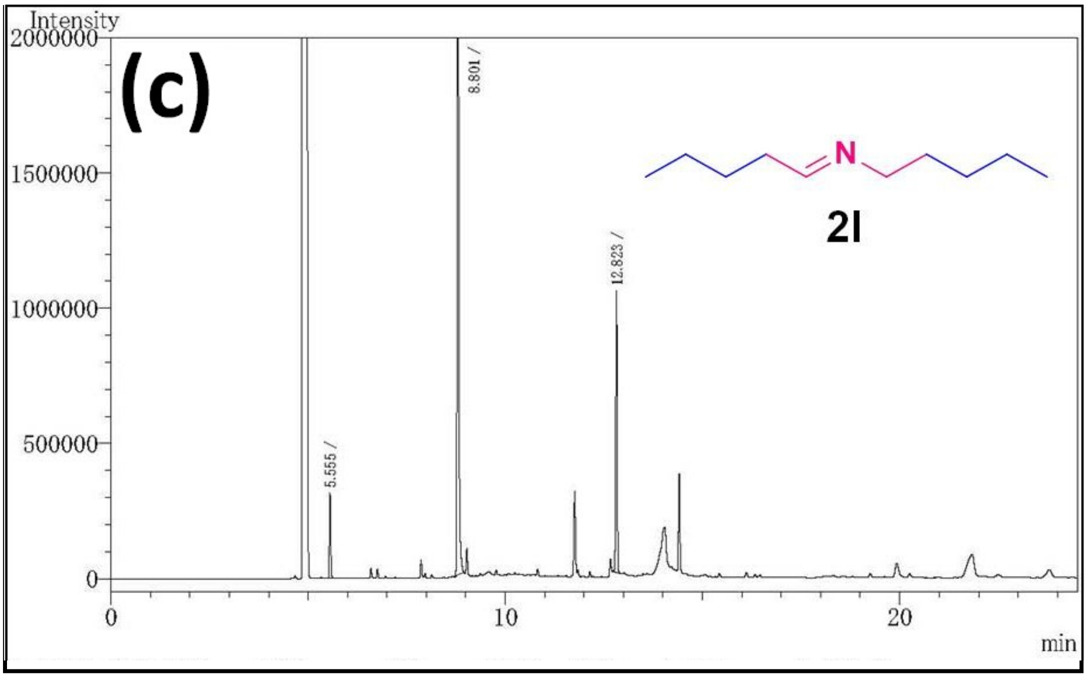

Fig. S28. (a) ^{1}H NMR spectrum, (b) ^{13}C NMR spectrum and (c) GC chromatogram of *N*-pentylidenepentan-1-amine (**2l**).

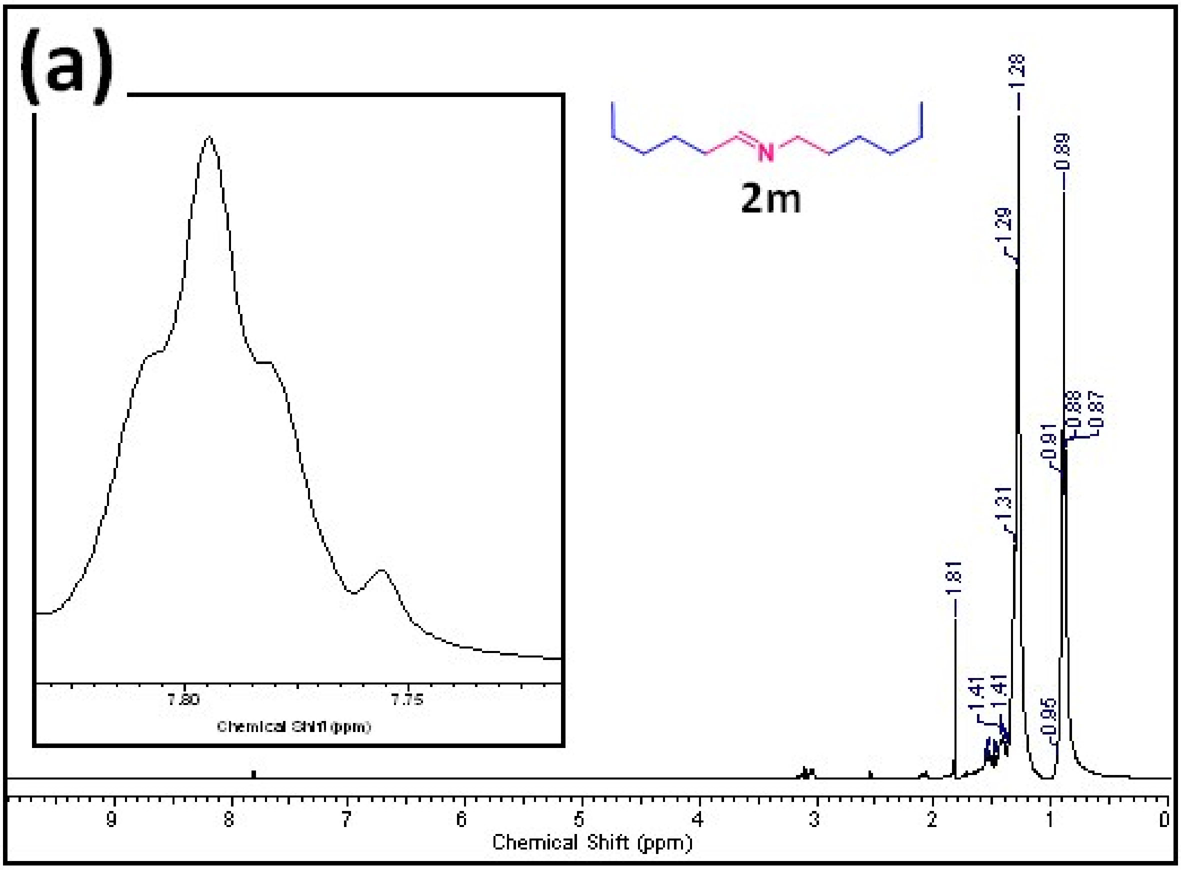
(a)
2m
1.28
1.29
0.89
0.88
0.87
0.91
1.31
1.81
1.41
1.41
0.95
7.80
7.75
Chemical Shift (ppm)
9
8
7
6
5
4
3
2
1
0
Chemical Shift (ppm)

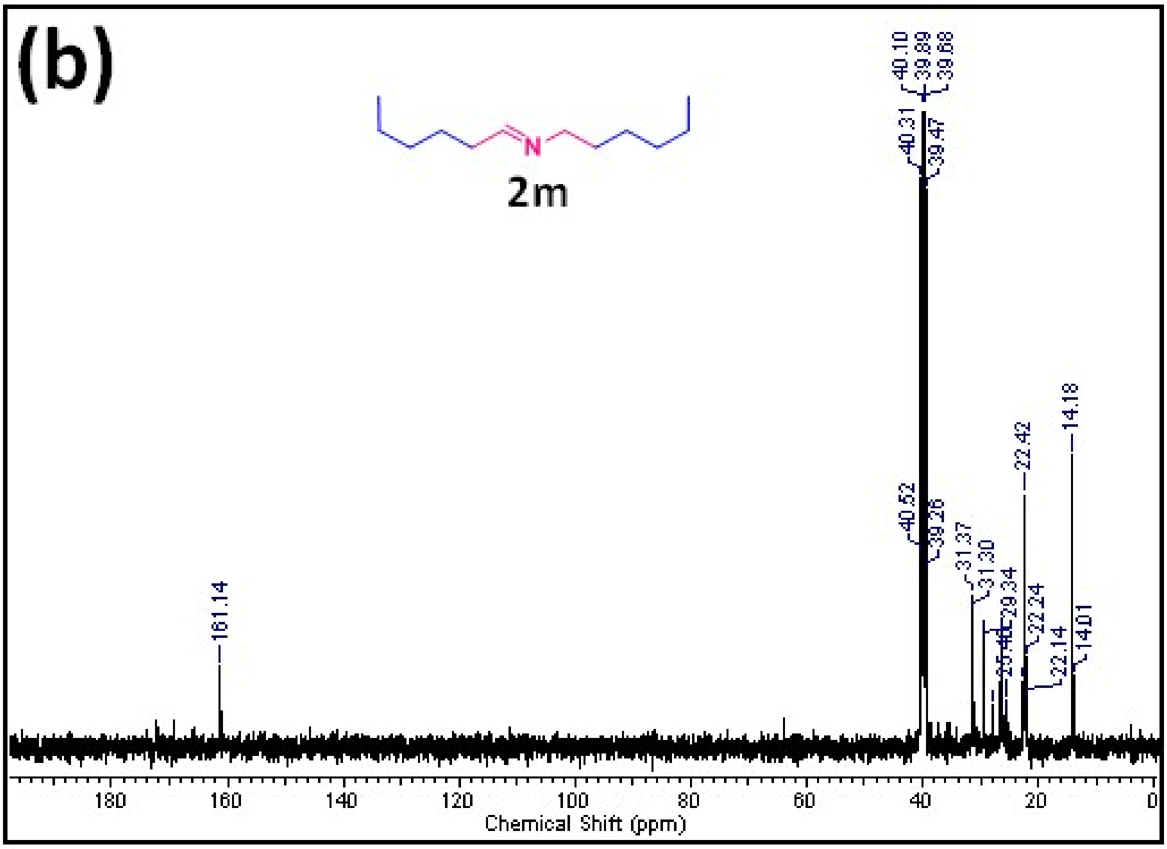
(b)
2m
40.10
39.89
39.68
40.31
39.47
14.18
22.42
40.52
39.26
31.37
31.30
29.34
22.24
22.14
14.01
161.14
180
160
140
120
100
80
60
40
20
0
Chemical Shift (ppm)

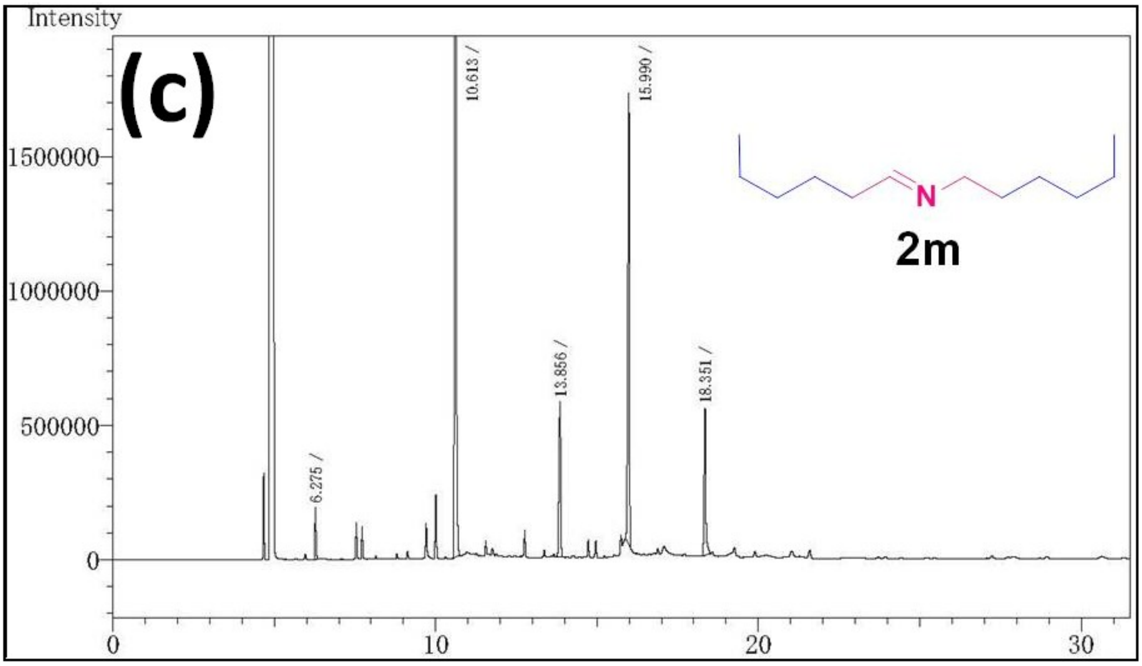

Fig. S29. (a) ^{1}H NMR spectrum, (b) ^{13}C NMR spectrum and (c) GC chromatogram of *N*-hexylidenehexan-1-amine (**2m**).

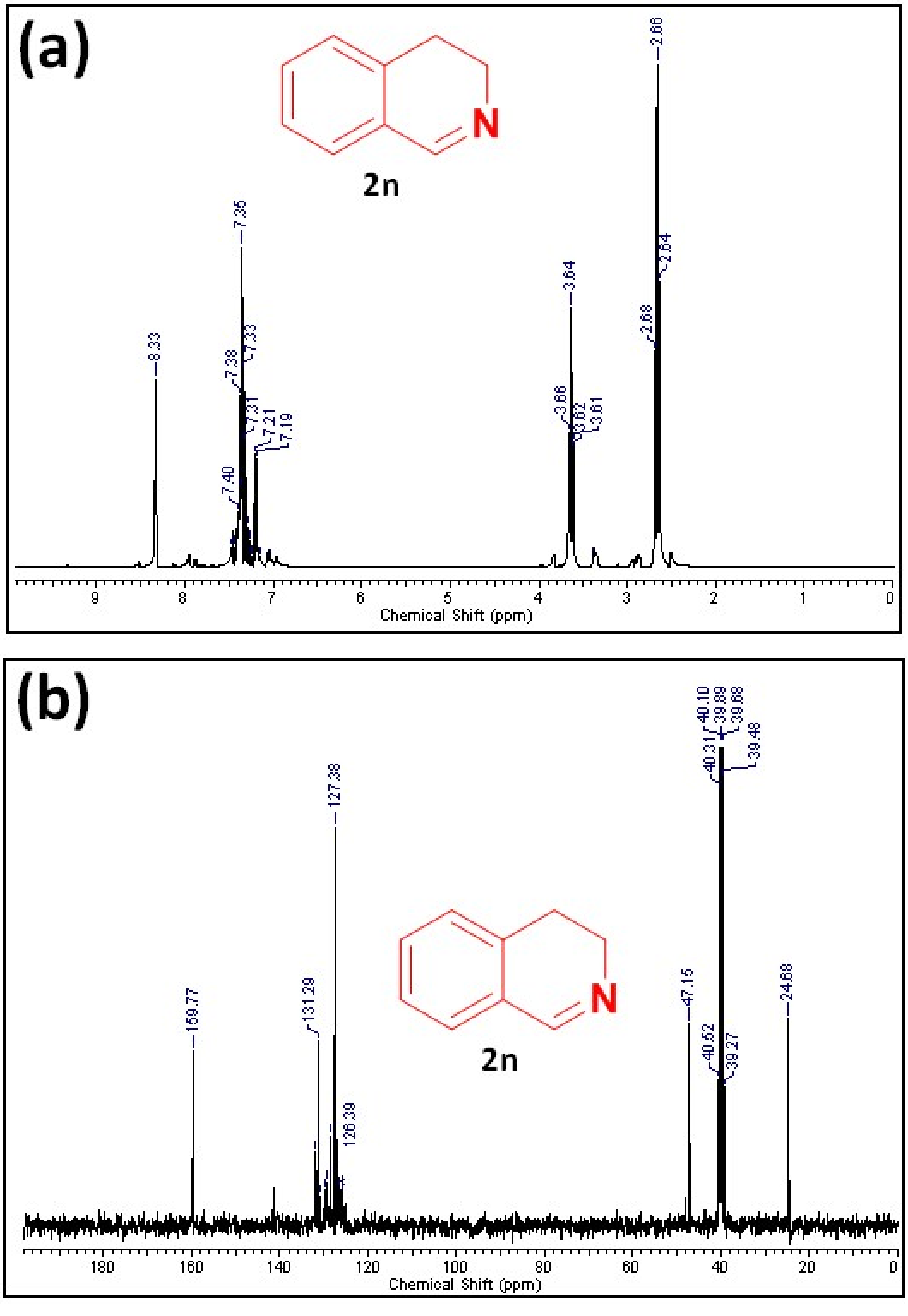

(a)
N
2n
8.33
7.40
7.38
7.35
7.33
7.31
7.21
7.19
3.66
3.64
3.62
3.61
2.68
2.66
2.64
9 8 7 6 5 4 3 2 1 0
Chemical Shift (ppm)
(b)
N
2n
159.77
131.29
127.38
126.39
47.15
40.52
40.31
40.10
39.89
39.68
39.48
39.27
24.68
180 160 140 120 100 80 60 40 20 0
Chemical Shift (ppm)

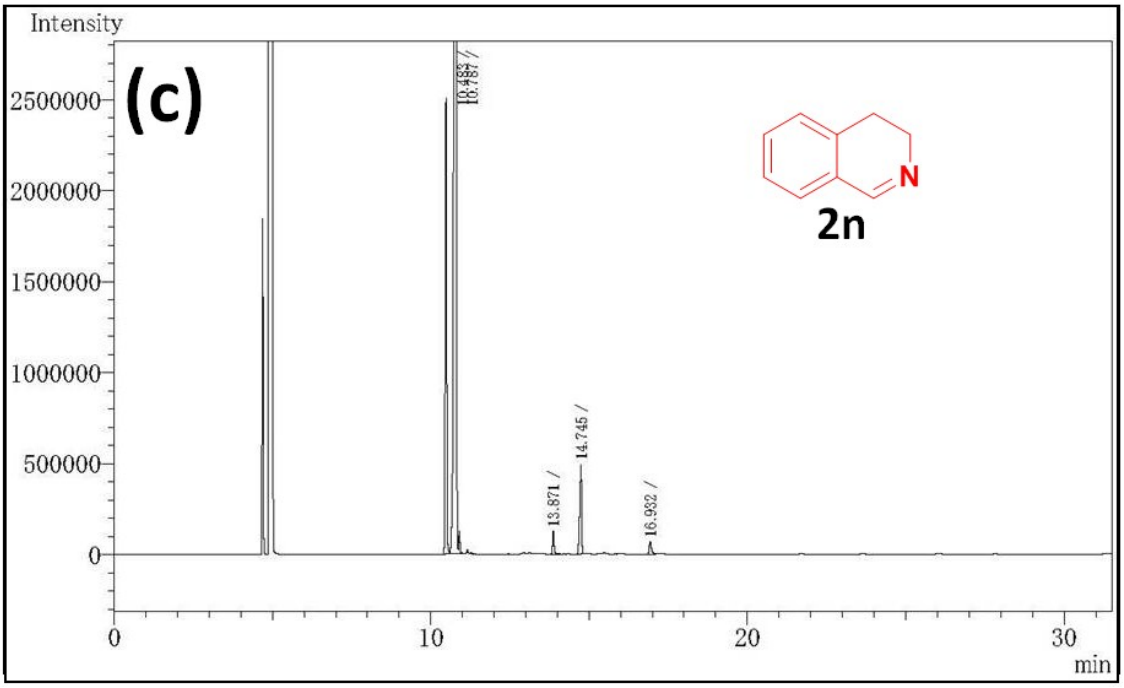

Fig. S30. (a) ^{1}H NMR spectrum, (b) ^{13}C NMR spectrum and (c) GC chromatogram of 3,4-dihydroisoquinoline (**2n**).

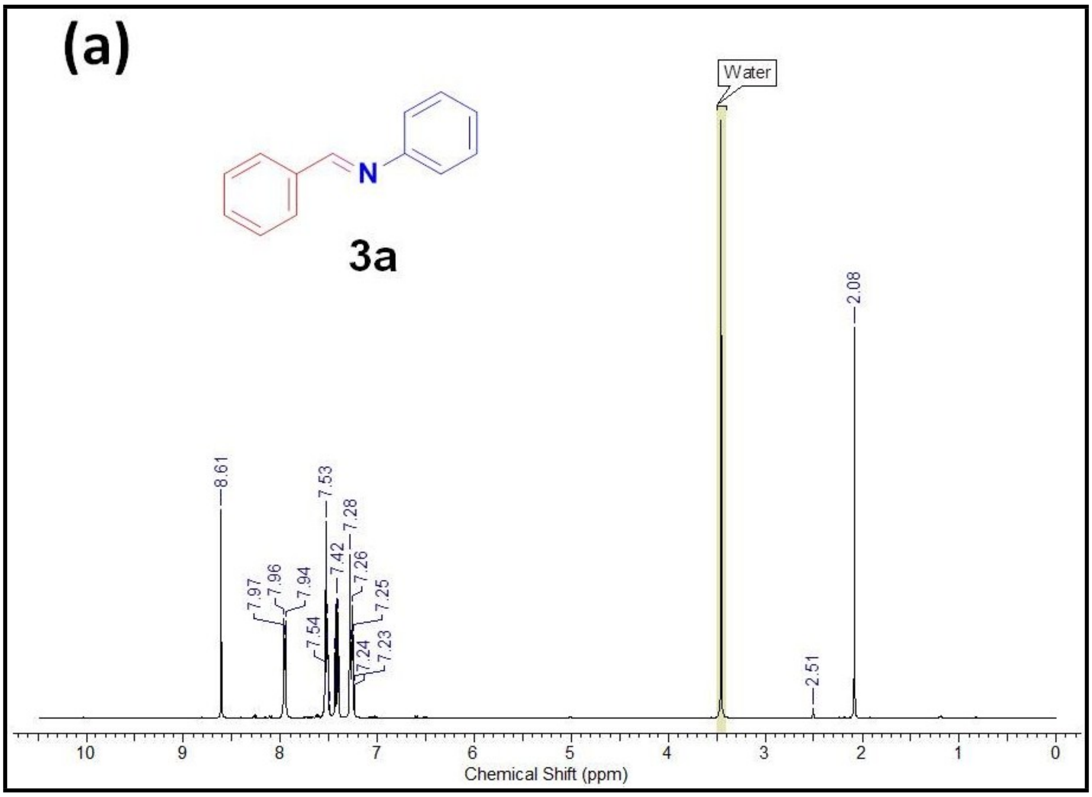
(a)
N
3a
Water
8.61
7.97
7.96
7.94
7.54
7.53
7.42
7.28
7.26
7.25
7.24
7.23
2.51
2.08
10
9
8
7
6
5
4
3
2
1
0
Chemical Shift (ppm)

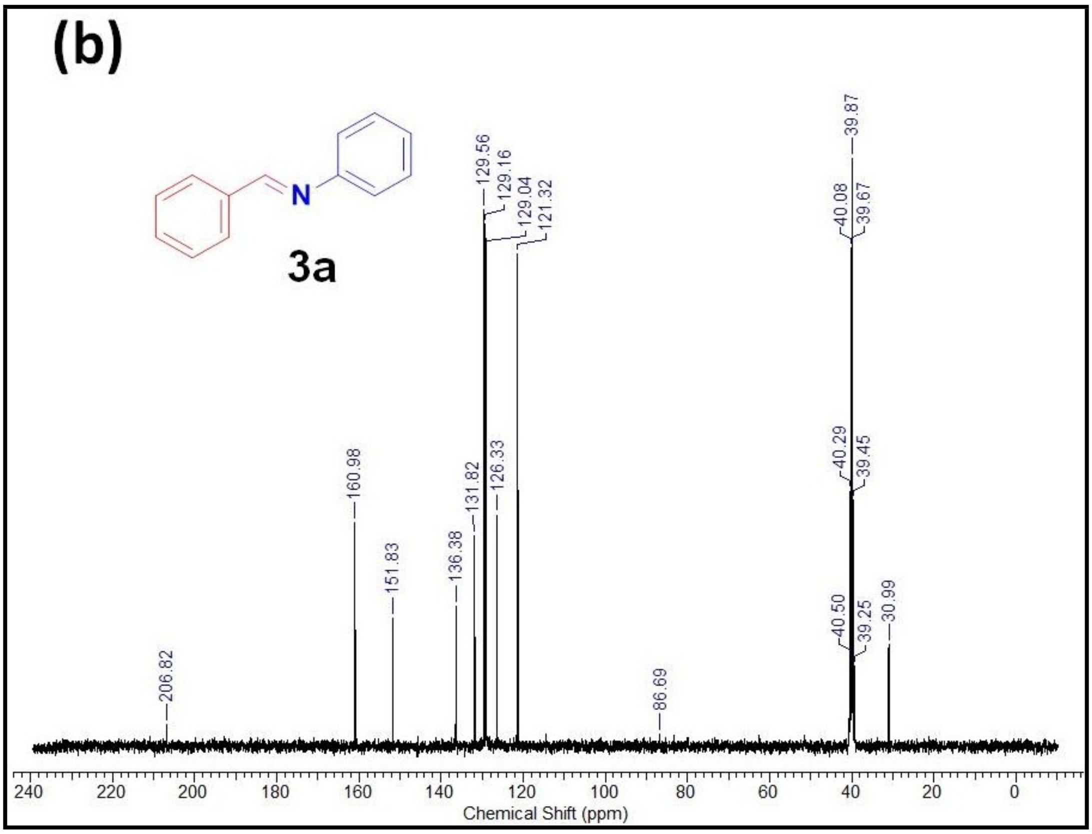
(b)
N
3a
206.82
160.98
151.83
136.38
131.82
129.56
129.16
129.04
126.33
121.32
86.69
40.50
40.29
40.08
39.87
39.67
39.45
39.25
30.99
240
220
200
180
160
140
120
100
80
60
40
20
0
Chemical Shift (ppm)

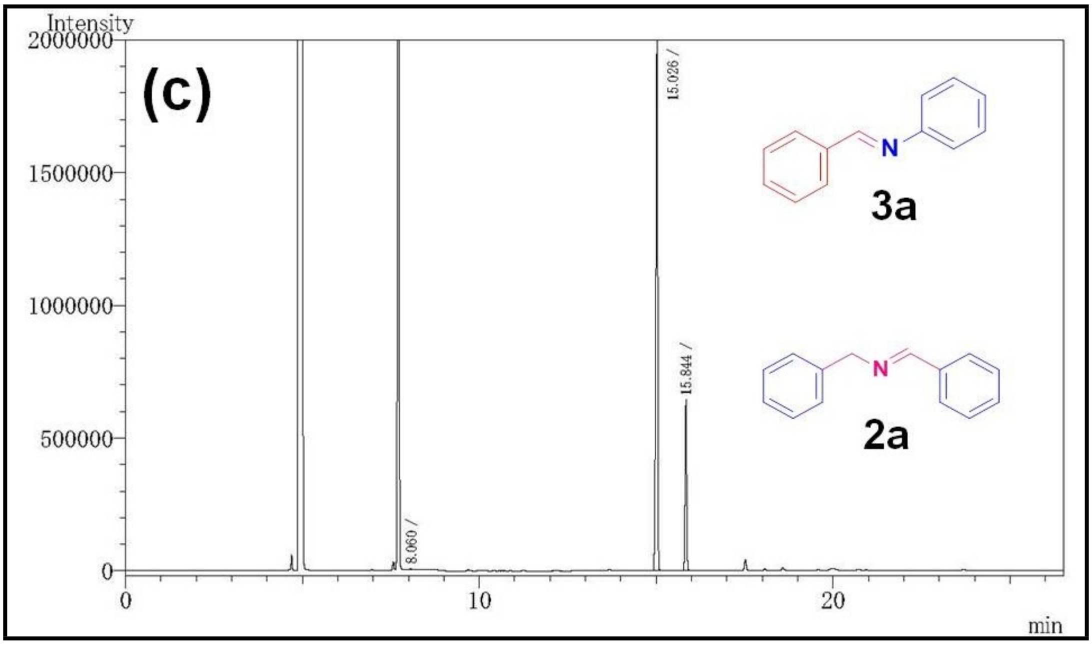

Fig. S31. (a) ^{1}H NMR spectrum, (b) ^{13}C NMR spectrum and (c) GC chromatogram of *N*-benzylideneaniline (**3a**).

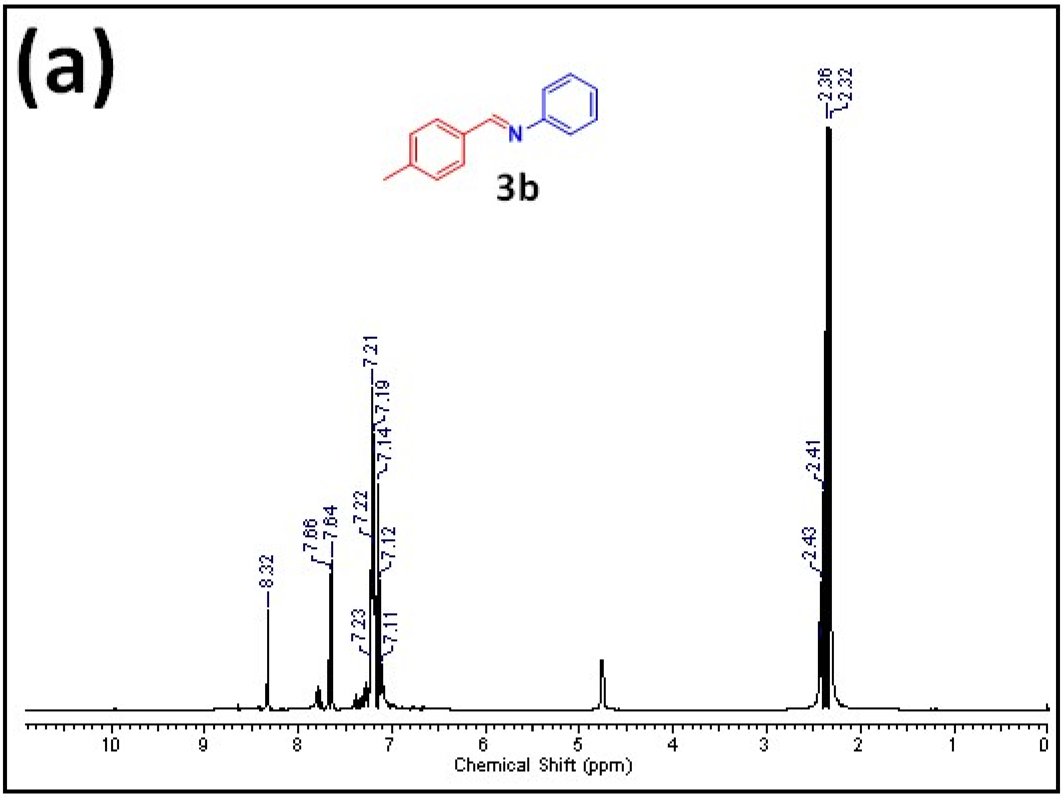
(a)
N
3b
8.32
7.66
7.64
7.23
7.22
7.21
7.19
7.14
7.12
7.11
2.43
2.41
2.36
2.32
10 9 8 7 6 5 4 3 2 1 0
Chemical Shift (ppm)

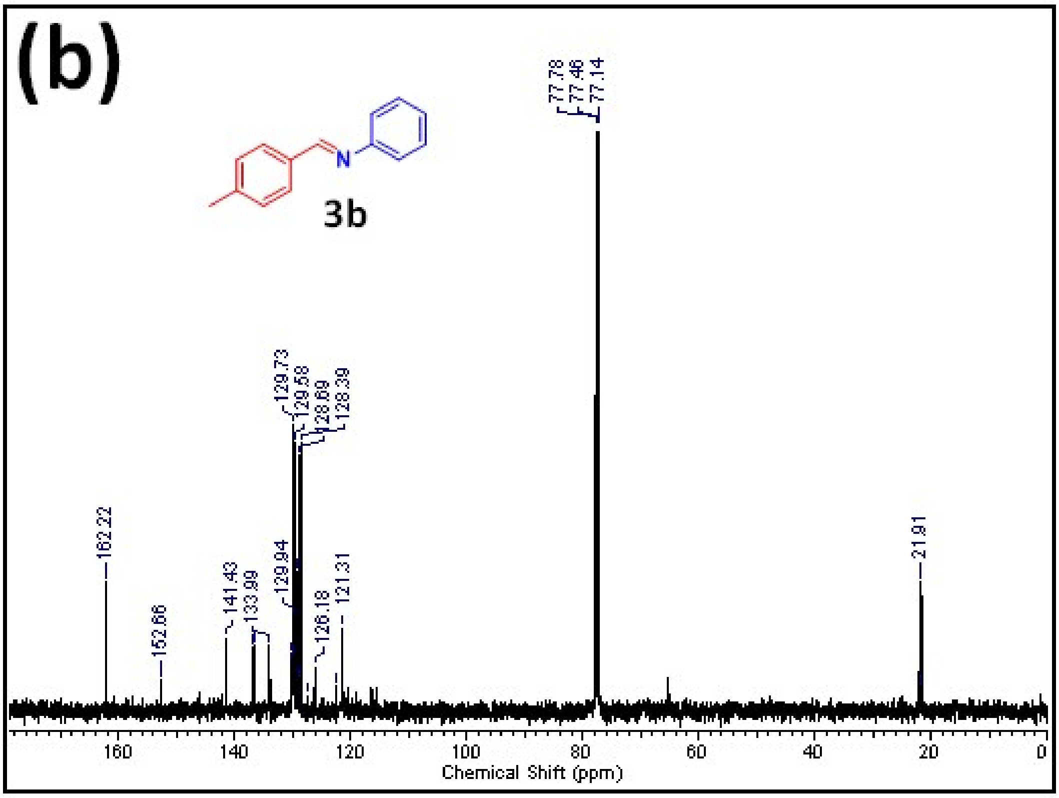
(b)
N
3b
162.22
152.66
141.43
133.99
129.94
129.73
129.58
128.69
128.39
126.18
121.31
77.78
77.46
77.14
21.91
160 140 120 100 80 60 40 20 0
Chemical Shift (ppm)

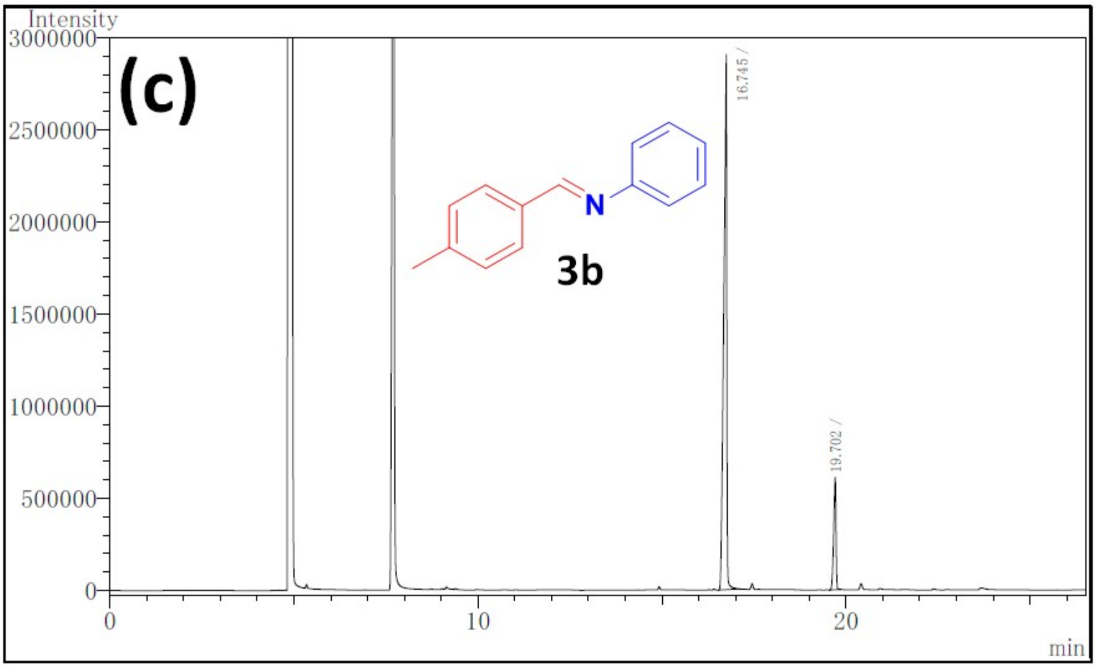

Fig. S32. (a) ^{1}H NMR spectrum, (b) ^{13}C NMR spectrum and (c) GC chromatogram of *N*-(4-Methylbenzylidene)aniline (**3b**).

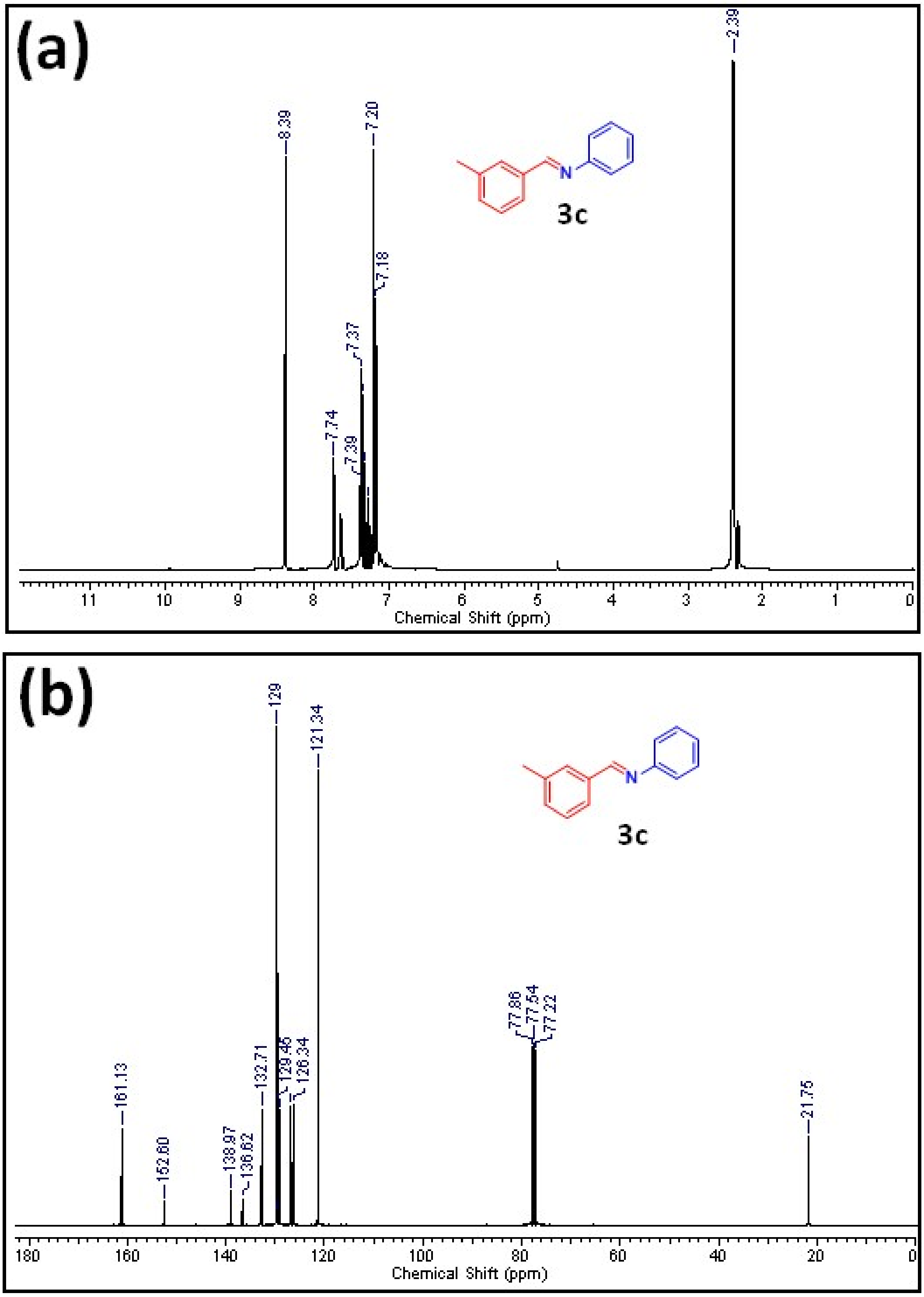
(a)
8.39
7.74
7.39
7.37
7.20
7.18
2.39
3c
N
11 10 9 8 7 6 5 4 3 2 1 0
Chemical Shift (ppm)
(b)
161.13
152.60
138.97
136.62
132.71
129
129.45
126.34
121.34
77.86
77.54
77.22
21.75
3c
N
180 160 140 120 100 80 60 40 20 0
Chemical Shift (ppm)

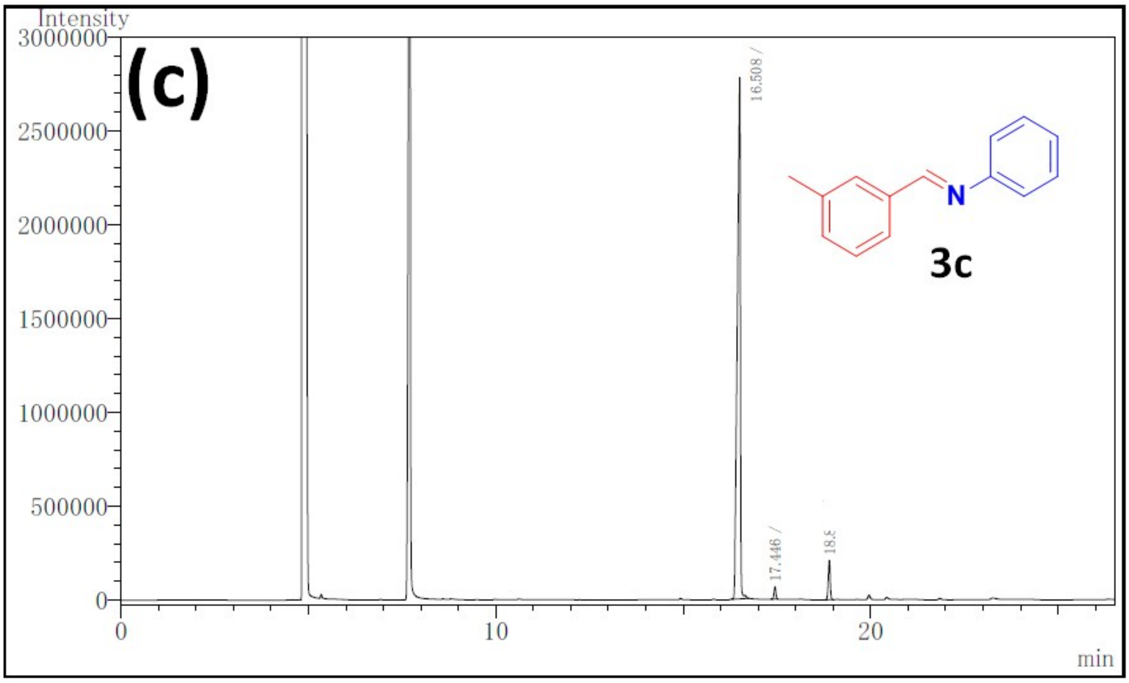

Fig. S33. (a) ^{1}H NMR spectrum, (b) ^{13}C NMR spectrum and (c) GC chromatogram of *N*-phenyl-1-(m-tolyl)methanimine (**3c**).

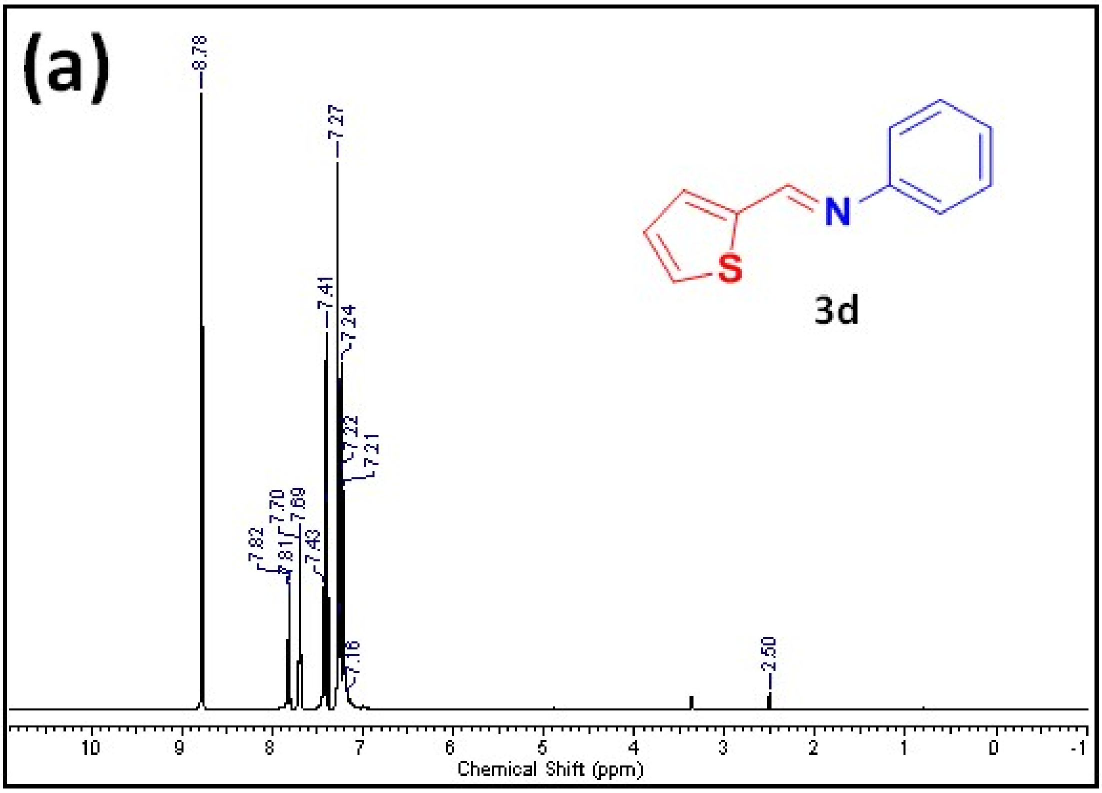
(a)
8.78
7.27
7.41
7.24
7.22
7.21
7.82
7.81
7.70
7.69
7.43
7.18
2.50
N
S
3d
10 9 8 7 6 5 4 3 2 1 0 -1
Chemical Shift (ppm)

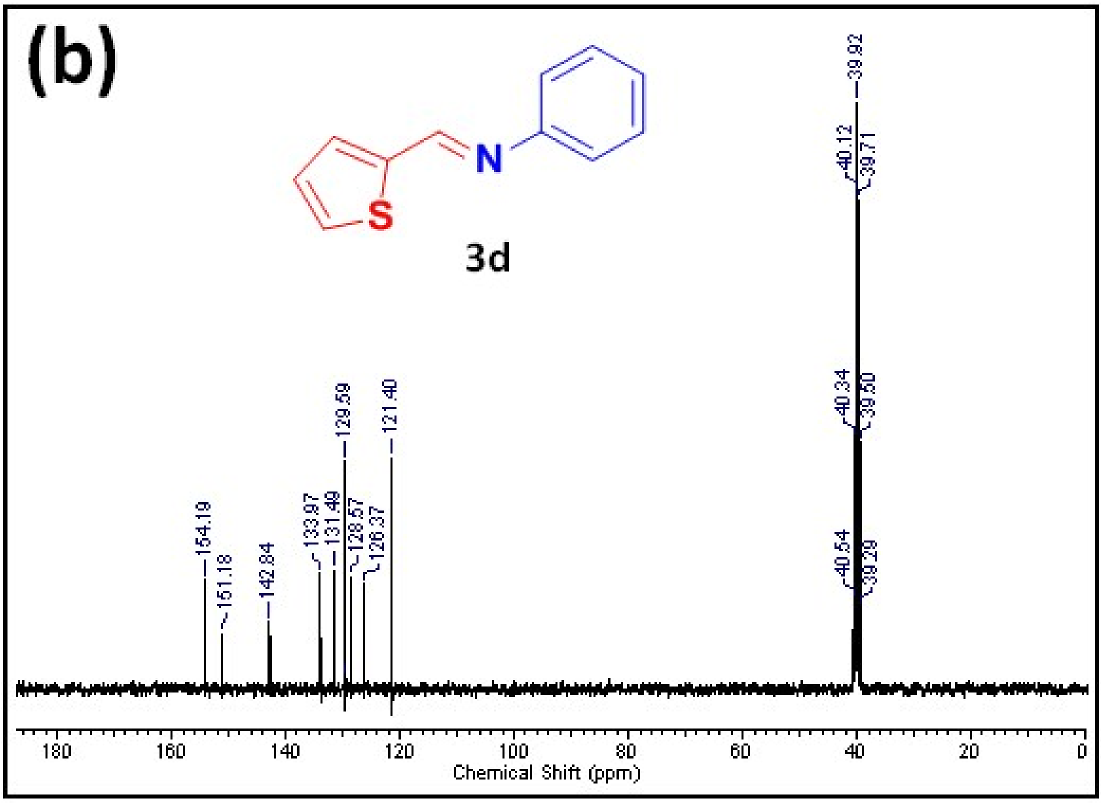
(b)
N
S
3d
39.92
40.12
39.71
40.34
39.50
40.54
39.29
154.19
151.18
142.84
133.97
131.49
129.59
128.57
126.37
121.40
180 160 140 120 100 80 60 40 20 0
Chemical Shift (ppm)

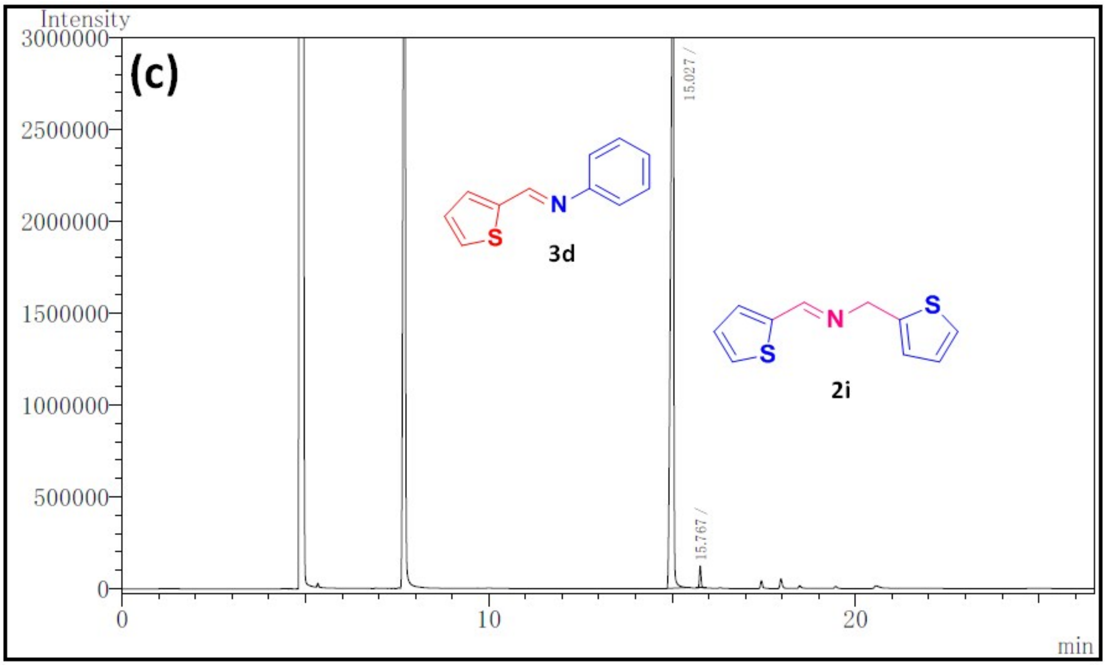

Fig. S34. (a) ^{1}H NMR spectrum, (b) ^{13}C NMR spectrum and (c) GC chromatogram of *N*-(thiophen-2-yl)-1-methanamine (**3d**).

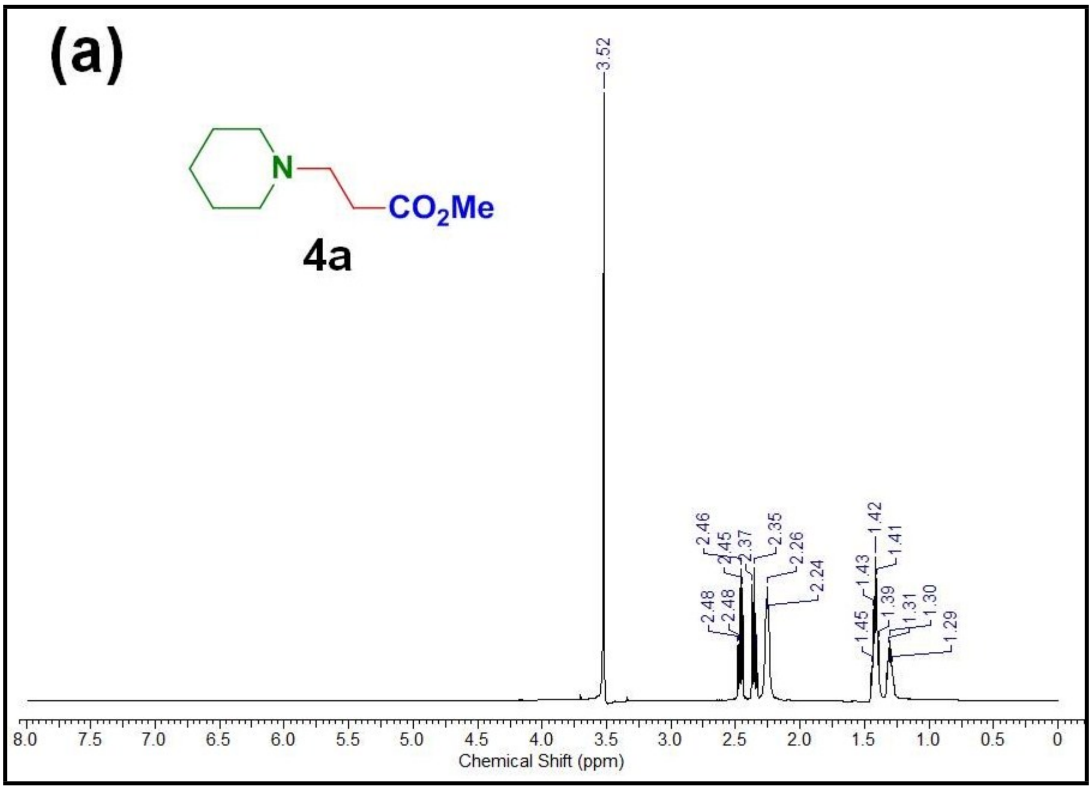
(a)
N
CO_2Me
4a
3.52
2.46
2.45
2.37
2.35
2.26
2.24
2.48
2.48
1.42
1.41
1.43
1.39
1.31
1.30
1.45
1.29
8.0 7.5 7.0 6.5 6.0 5.5 5.0 4.5 4.0 3.5 3.0 2.5 2.0 1.5 1.0 0.5 0
Chemical Shift (ppm)

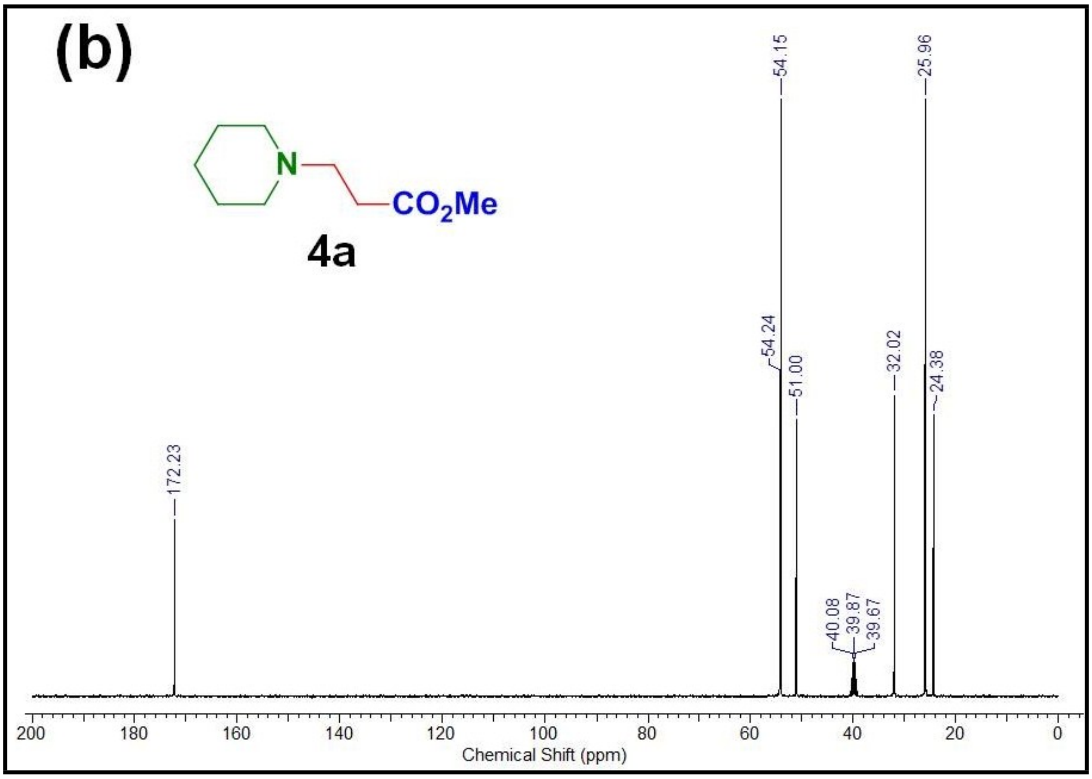
(b)
N
CO_2Me
4a
54.15
25.96
54.24
51.00
32.02
24.38
172.23
40.08
39.87
39.67
200 180 160 140 120 100 80 60 40 20 0
Chemical Shift (ppm)

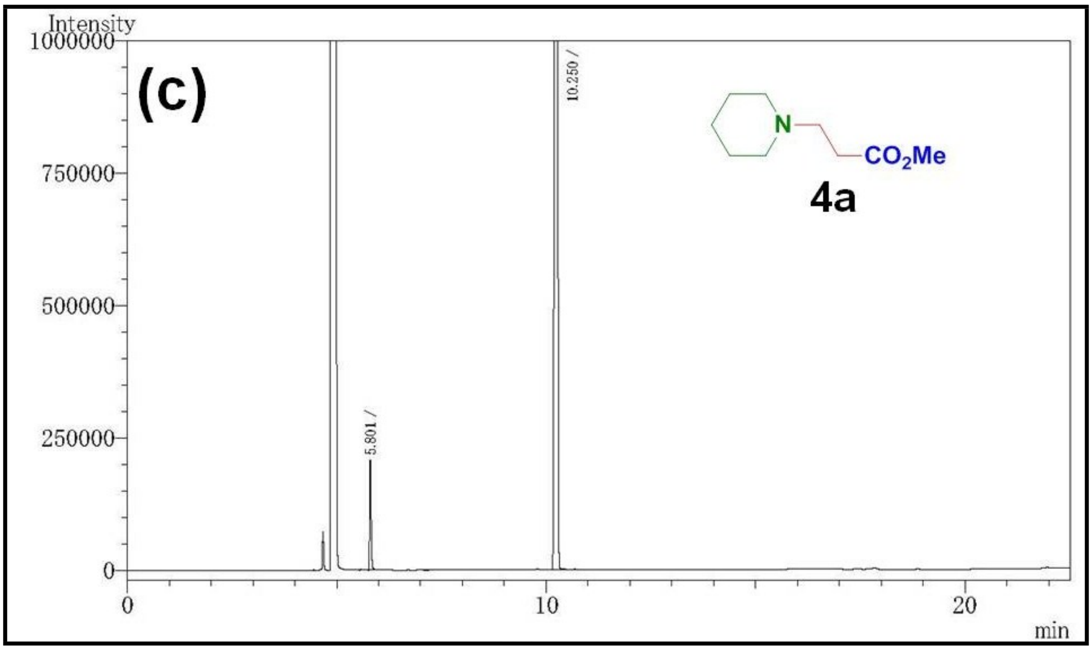

Fig. S35. (a) ^{1}H NMR spectrum, (b) ^{13}C NMR spectrum and (c) GC chromatogram of methyl 3-(piperidin-1-yl)propanoate (**4a**).

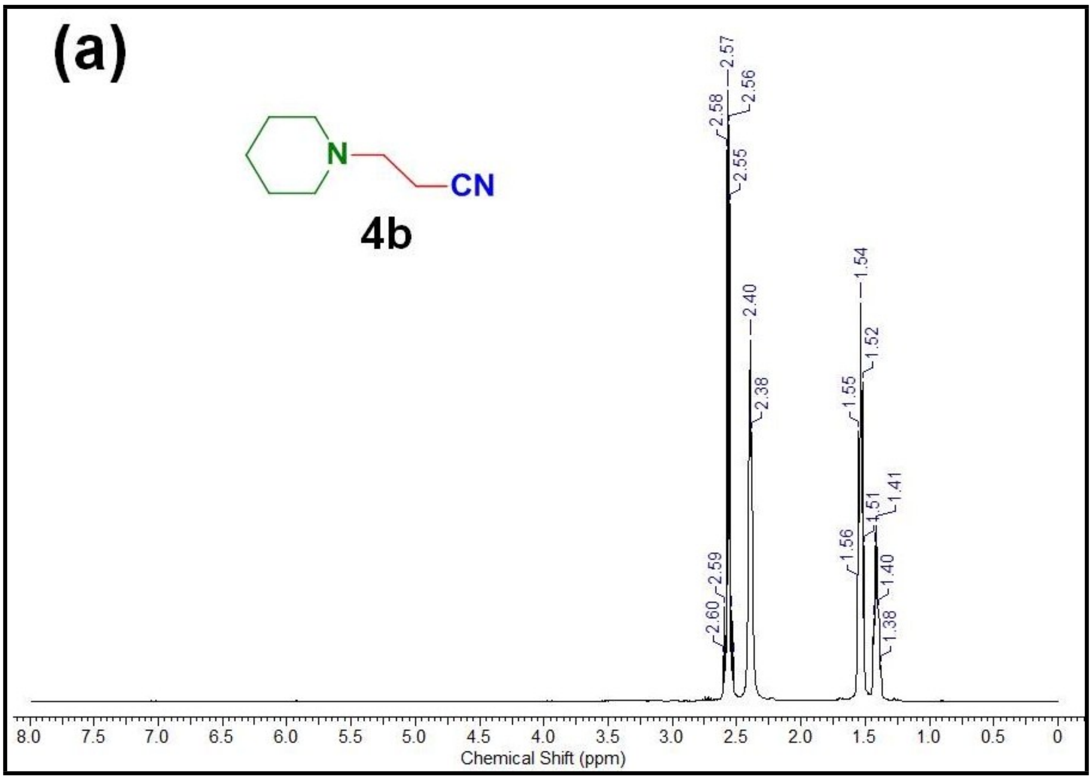
(a)
N
CN
4b
2.57
2.56
2.58
2.55
2.40
2.38
1.54
1.52
1.55
1.51
1.41
1.56
1.40
1.38
2.59
2.60
8.0 7.5 7.0 6.5 6.0 5.5 5.0 4.5 4.0 3.5 3.0 2.5 2.0 1.5 1.0 0.5 0
Chemical Shift (ppm)

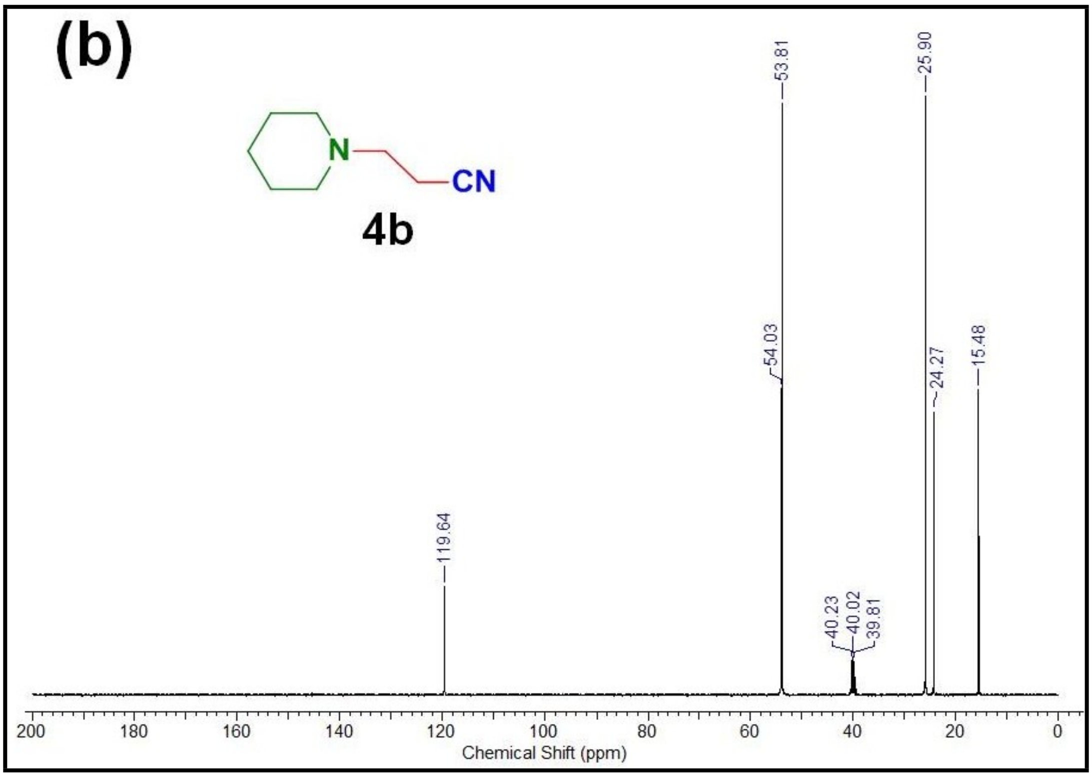
(b)
N
CN
4b
53.81
25.90
54.03
24.27
15.48
119.64
40.23
40.02
39.81
200 180 160 140 120 100 80 60 40 20 0
Chemical Shift (ppm)

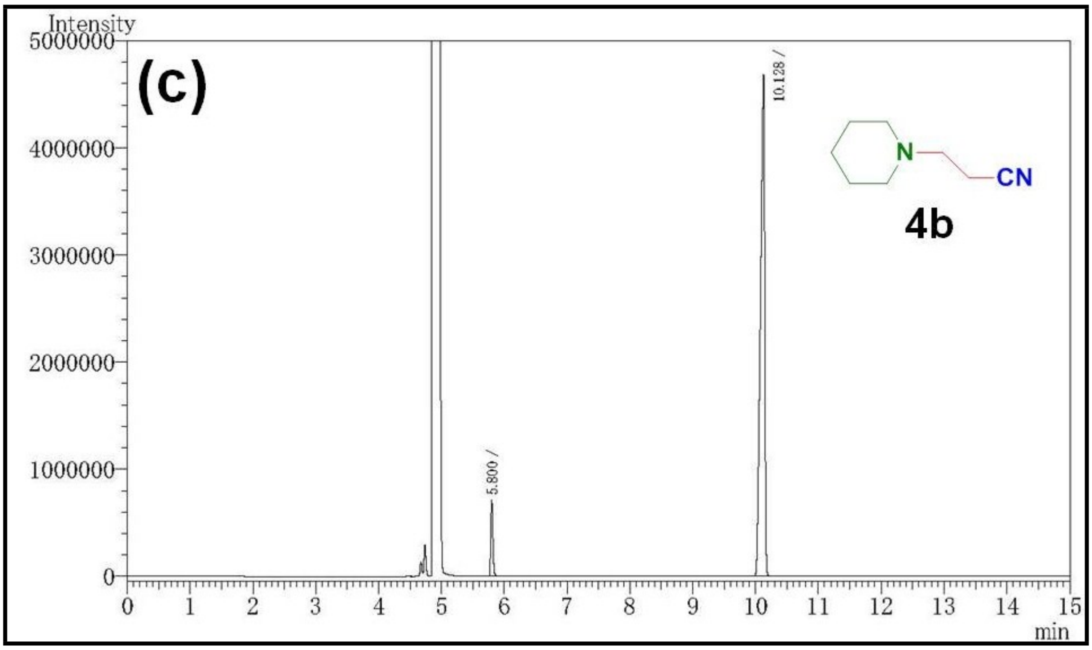

Fig. S36. (a) ^{1}H NMR spectrum, (b) ^{13}C NMR spectrum and (c) GC chromatogram of 3-(piperidin-1-yl)propanenitrile (**4b**).

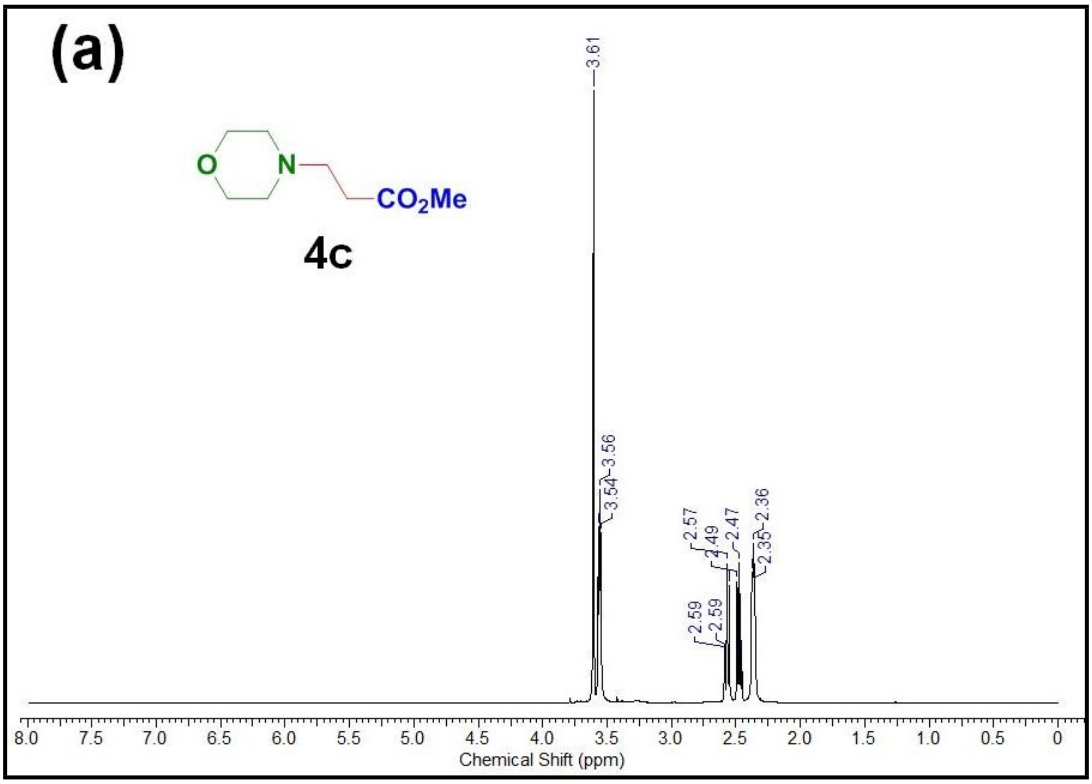
(a)
O
N
CO2Me
4c
3.61
3.56
3.54
2.57
2.49
2.47
2.36
2.35
2.59
2.59
8.0 7.5 7.0 6.5 6.0 5.5 5.0 4.5 4.0 3.5 3.0 2.5 2.0 1.5 1.0 0.5 0
Chemical Shift (ppm)

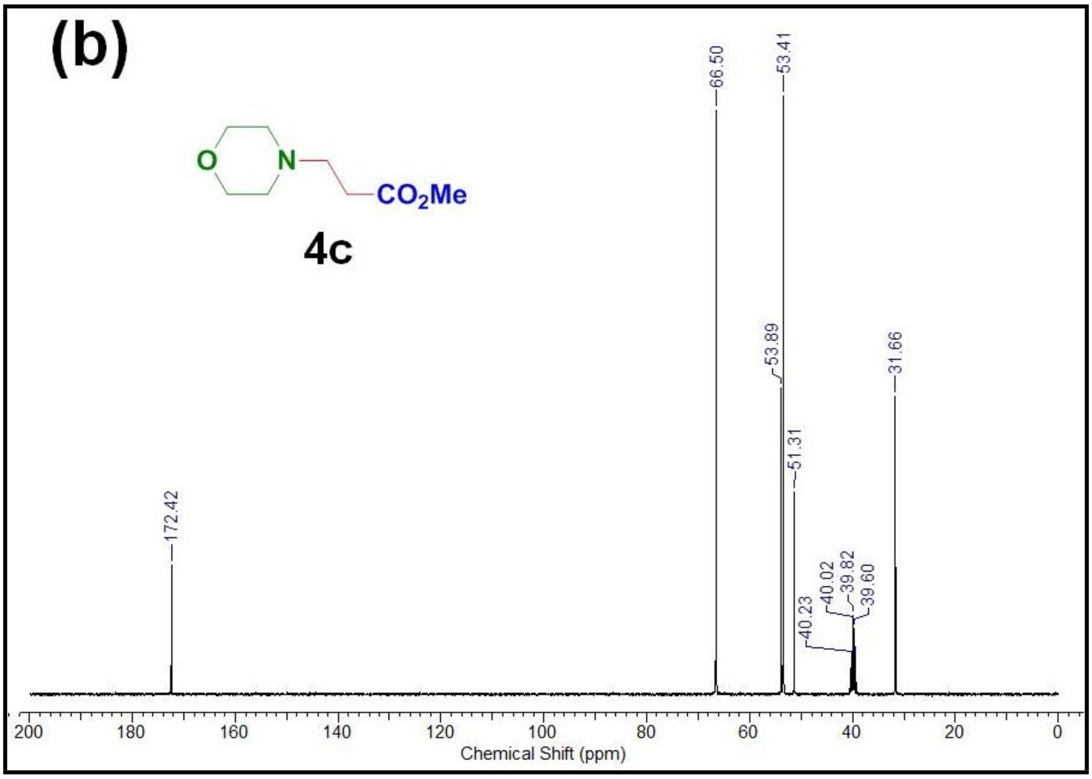
(b)
O
N
CO2Me
4c
66.50
53.41
53.89
31.66
51.31
172.42
40.23
40.02
39.82
39.60
200 180 160 140 120 100 80 60 40 20 0
Chemical Shift (ppm)

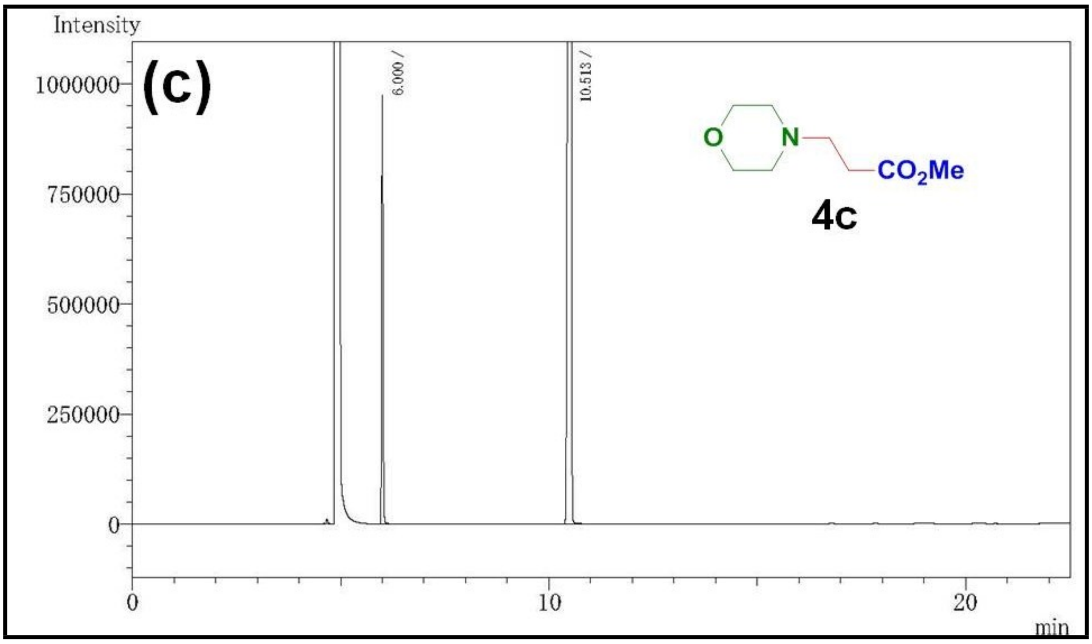

Fig. S37. (a) ^{1}H NMR spectrum, (b) ^{13}C NMR spectrum and (c) GC chromatogram of methyl 3-morpholinopropanoate (**4c**).

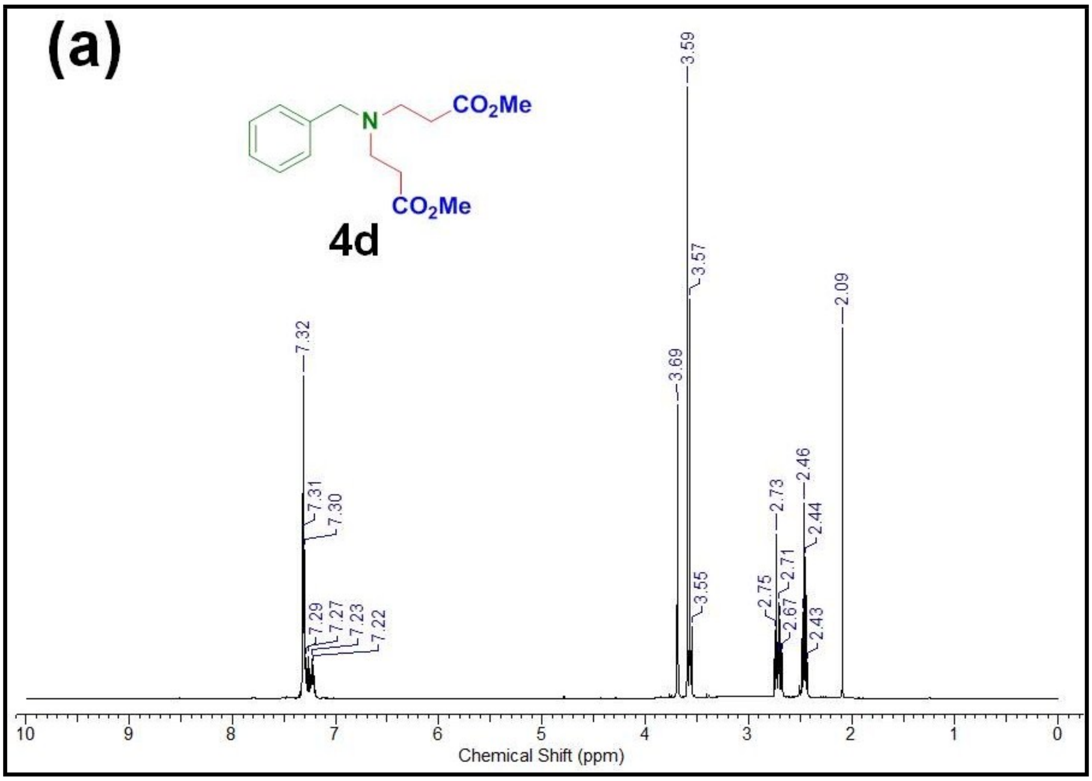
(a)
CO2Me
N
CO2Me
4d
Chemical Shift (ppm)

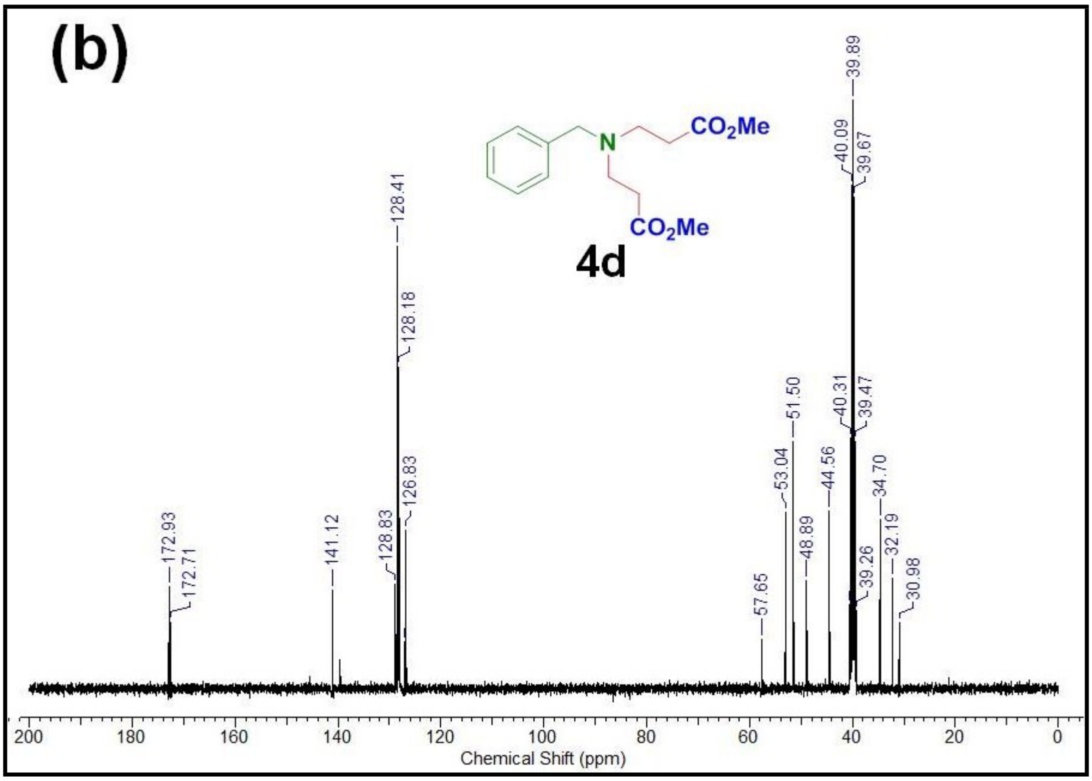
(b)
CO2Me
N
CO2Me
4d
Chemical Shift (ppm)

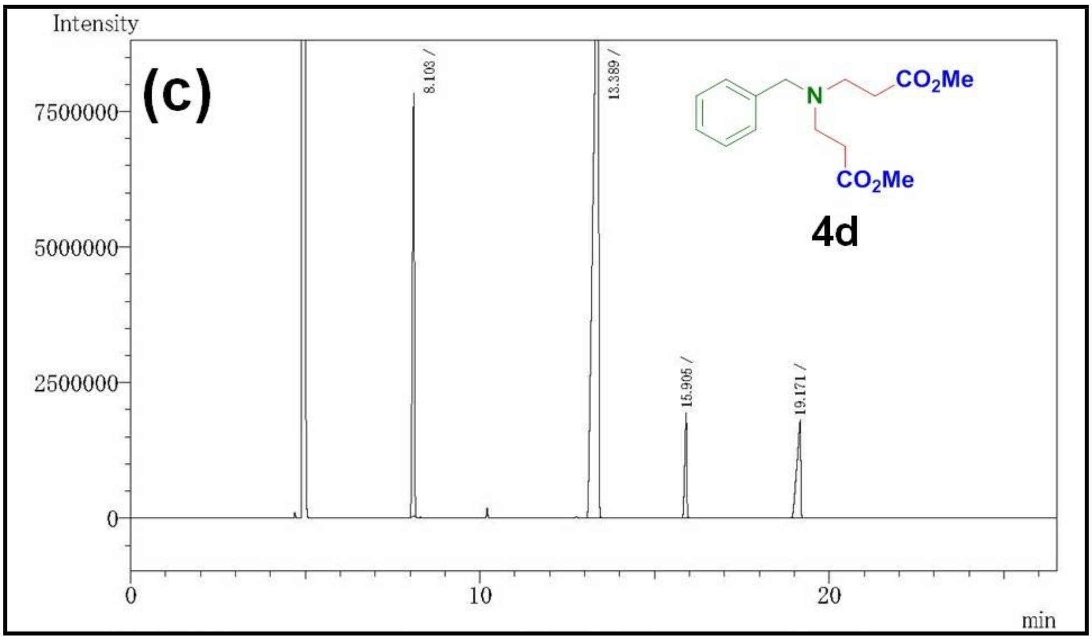

Fig. S38. (a) ^{1}H NMR spectrum, (b) ^{13}C NMR spectrum and (c) GC chromatogram of 3,3'-(*N*-benzylimino)dipropionic acid dimethyl ester (**4d**).

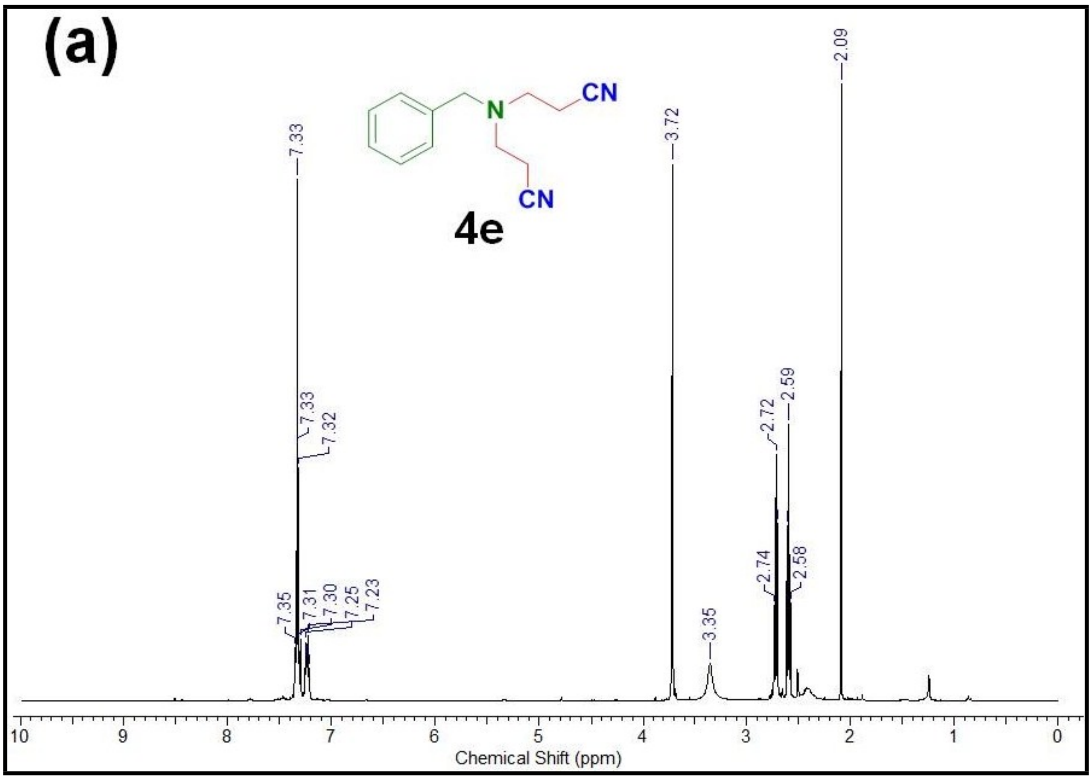
(a)
CN
N
CN
4e
7.33
7.33
7.32
7.35
7.31
7.30
7.25
7.23
3.72
3.35
2.72
2.74
2.59
2.58
2.09
10 9 8 7 6 5 4 3 2 1 0
Chemical Shift (ppm)

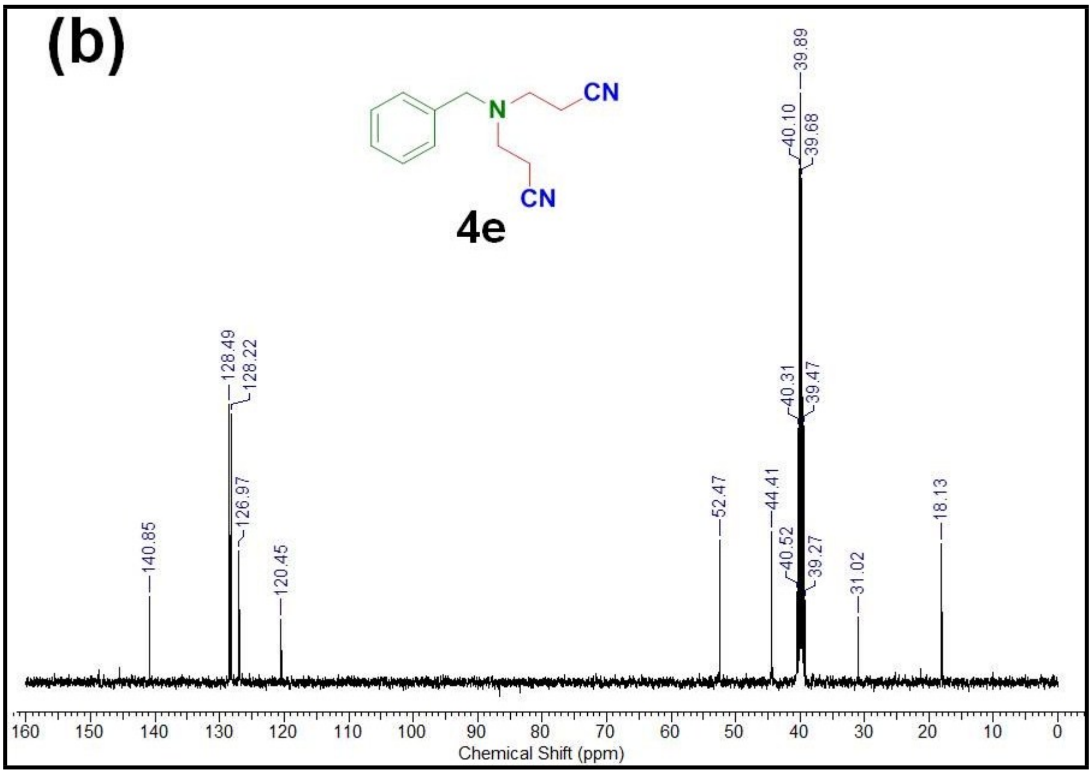
(b)
CN
N
CN
4e
140.85
128.49
128.22
126.97
120.45
52.47
44.41
40.52
40.31
40.10
39.89
39.68
39.47
39.27
31.02
18.13
160 150 140 130 120 110 100 90 80 70 60 50 40 30 20 10 0
Chemical Shift (ppm)

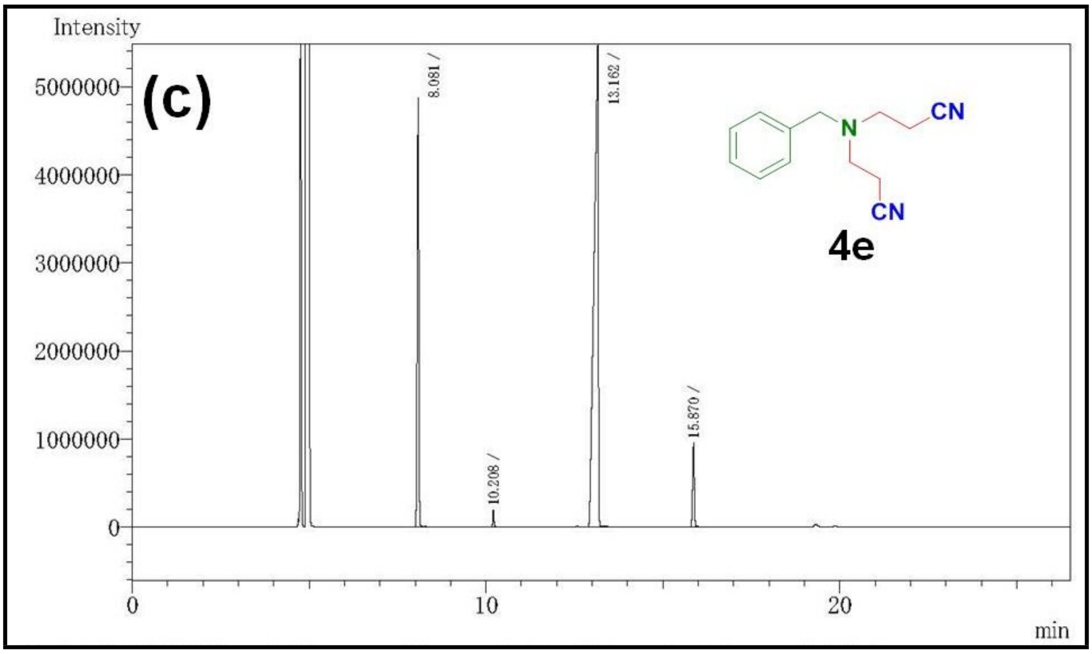

Fig. S39. (a) ^{1}H NMR spectrum, (b) ^{13}C NMR spectrum and (c) GC chromatogram of 3,3'-(benzylazanediyl)dipropanenitrile (**4e**).

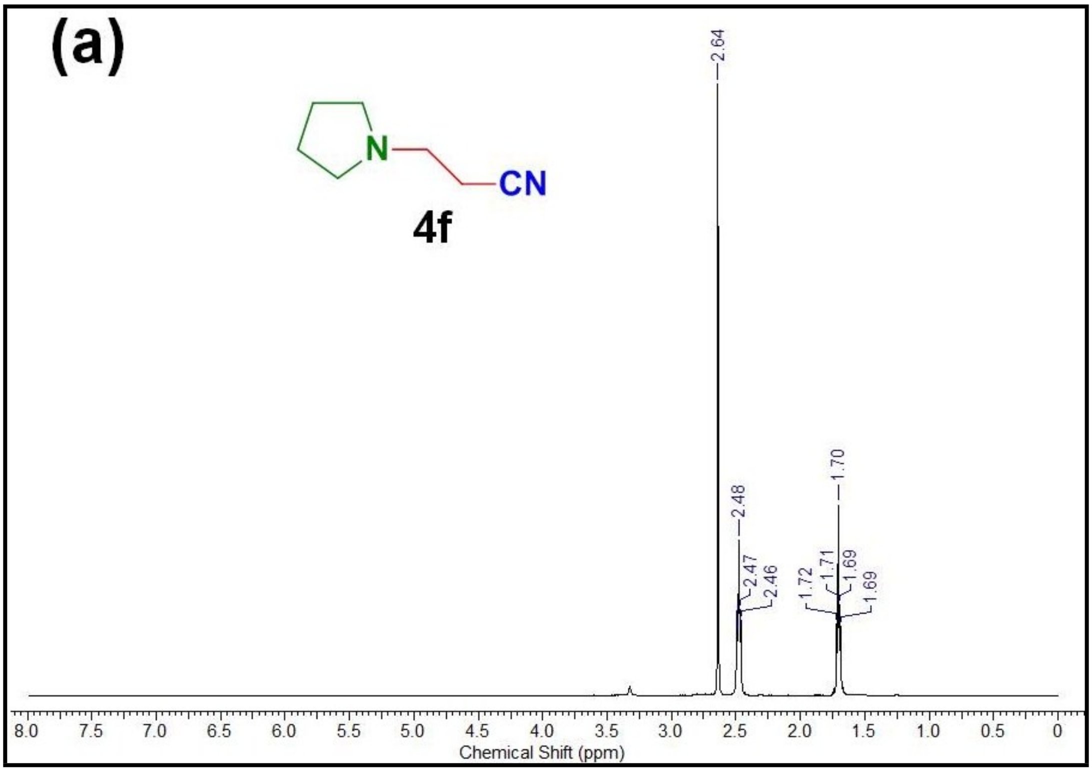

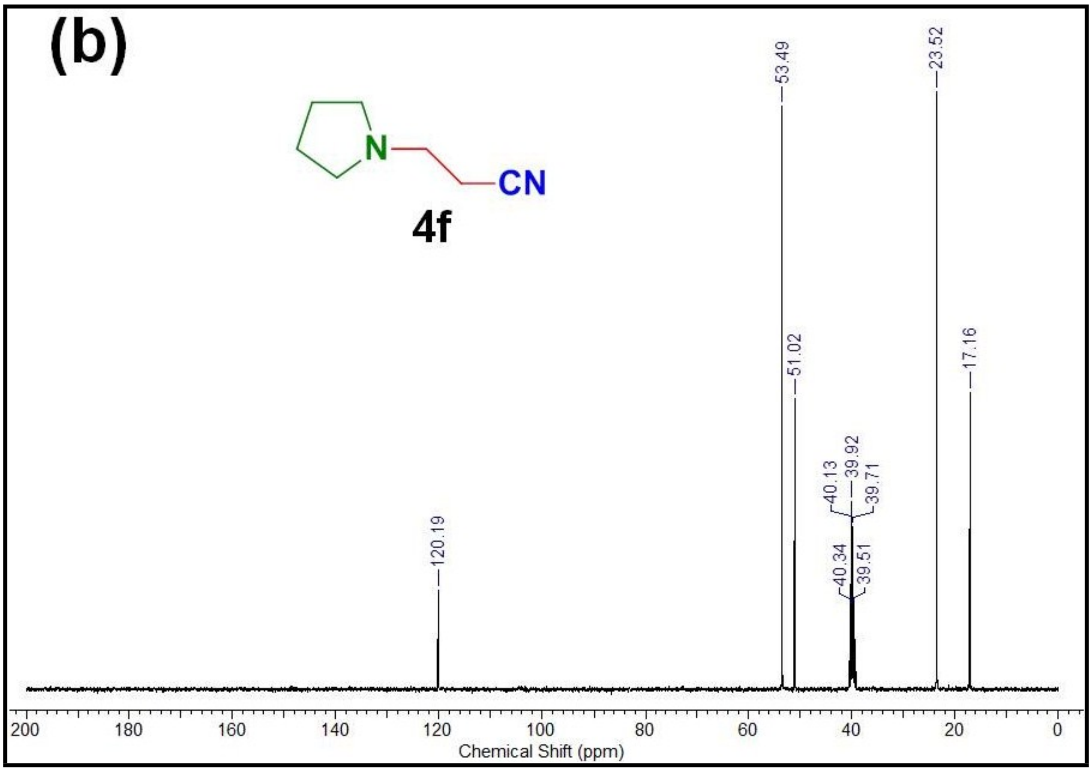

Fig. S40 (a) ^{1}H and (b) ^{13}C NMR spectrum of 3-(pyrrolidin-1-yl)propanenitrile (**4f**).

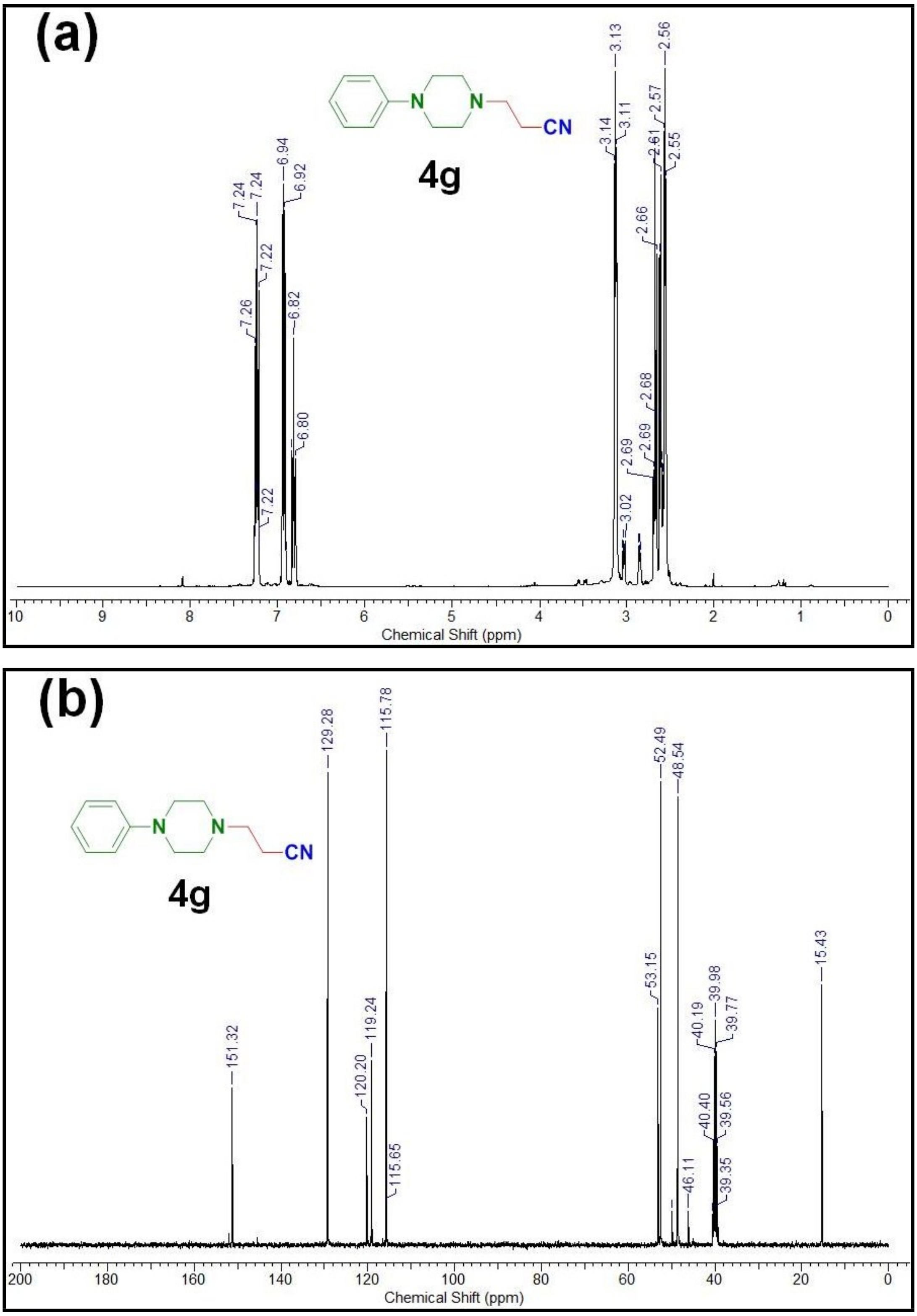

Fig. S41. (a) ^{1}H and (b) ^{13}C NMR spectrum of 3-(4-phenylpiperazin-1-yl) propanenitrile (**4g**).
